# Assessing the Psychometric Properties of the Child Behaviour Checklist in the ABCD Study

**DOI:** 10.1111/desc.70216

**Published:** 2026-05-10

**Authors:** Kane Pavlovich, Toby Constable, Alex Fornito, Jeggan Tiego

**Affiliations:** ^1^ School of Psychological Sciences Turner Institute for Brain and Mental Health, and Monash Biomedical Imaging Victoria Australia

## Abstract

The Child Behaviour Checklist (CBCL) is a widely used measure of psychopathology in developmental research and serves as the primary measure in the landmark Adolescent Brain Cognitive Development study (*N* = 11,861; 8–11 years). However, its psychometric properties in this sample have not been assessed in detail. Analyses using a multi‐method framework (CFA, B‐CFA, FMM, IRT, ESEM) indicate that a commonly used bifactor structure of the CBCL does not fit the data and instead the scale can be adequately described as unidimensional. Moreover, seven of eight subscales demonstrate insufficient construct validity and are not valid indicators of any higher‐order models. All exhibit poor measurement precision below the mean of the latent traits, and item‐level exploratory methods produce unstable factors with weak discriminant validity. These findings suggest caution in using the CBCL for dimensional measurement in this dataset and highlight the need for measures explicitly developed and validated for dimensional psychopathology in population‐scale research.

**Summary:**

- The CBCL is a widely used measure of child and adolescent psychopathology, but its psychometric properties in the ABCD study cohort remain unclear.
- Using a multi‐method approach we show that the CBCL is unidimensional and is not well represented by the commonly used bifactor model.
- CBCL subscales demonstrate insufficient construct validity and demonstrate poor measurement precision across the latent trait continuum.
- In combination, our findings caution against the use of the CBCL for dimensional measurement of developmental psychopathology in the ABCD study cohort.

## 1 Introduction

A major challenge in psychiatric research is the reliable and valid assessment of psychopathological symptoms (Nikolaidis et al. 2022; Tiego, Martin, et al. 2023). A plethora of scales have been developed to assess a broad range of symptoms, each making assumptions about the underlying latent architecture of mental health symptoms (Anvari et al. 2025; Fried 2017). For observer, self‐report, or informant‐report scales to offer useful probes of psychopathology, the measures must be both reliable and valid. A measure is reliable if it is consistent in measurement across items, measurement occasions and raters, and quantifies meaningful individual difference variance with minimal error variance (Xing and Zuo 2018). A measure can be valid with respect to multiple criteria (Strauss and Smith 2009), but here we focus on construct validity, by which a set of indicators (e.g., items) reflect the concept they were designed to measure (Strauss and Smith 2009; Goodwin 1999). Specifically, we focus on convergent validity (one aspect of construct validity), defined as the degree to which indicators show strong coherence and shared variance in converging on the same construct (Cheung et al. 2024).

Assessing psychopathology in children and adolescents presents unique measurement challenges for reliability and validity. Developmental factors significantly impact symptom manifestation, such that behaviours considered normative at one developmental stage (e.g., temper tantrums in preschoolers) may indicate pathology at another (e.g., in school‐aged children) (Speranza et al. 2023; Wakschlag et al. 2018). These developmental complexities are compounded by measurement limitations that include the unreliability of child self‐reports and low concordance rates between multi‐informant assessments of child psychopathology (De Los Reyes et al. 2015).

One of the most widely‐used measures of psychopathology for children and adolescents is the Child Behaviour Checklist (CBCL). The CBCL was initially developed to assess emotional and behavioural problems in children and adolescents, with the primary goal of differentiating clinically referred from non‐referred youth to ensure high clinical utility (Edelbrock and Achenbach 1980; Achenbach 2009). Accordingly, the measure has shown high criterion validity, defined as the degree to which an instrument's scores correlate with a gold standard outcome or clinically relevant criterion (Cronbach and Meehl 1995), across diverse cultural contexts (Ivanova et al. 2007). Initial research using exploratory factor analysis (EFA) demonstrated that the 113 core problem items clustered into eight empirically‐derived syndrome scales; Anxious/Depressed, Withdrawn/Depressed, Somatic Problems, Attention Problems, Thought Problems, Rule‐Breaking Behaviour and Aggressive Behaviour. Five of these scales in turn coalesce around two higher‐order factors: Internalising (comprised of Anxious/Depressed, Withdrawn/Depressed, Somatic Problems) and Externalising (comprised of Rule‐Breaking Behaviour and Aggressive Behaviour) (Achenbach 2009). These two broad constructs are fundamental to many dimensional models of psychopathology in youth and adults, and are often moderately correlated, reflecting high‐comorbidity (Kotov et al. 2017; Caspi et al. 2014).

Subsequent research has used Confirmatory Factor Analysis (CFA) to verify the latent dimensional structure of the CBCL (e.g., Ivanova et al. 2007; Pandolfi et al. 2012). Unlike EFA, CFA places strict constraints on the covariance matrix, such that item cross loadings on their non‐target primary factors, as well as correlations between the eight first‐order factors not clustered into Internalising and Externalising second‐order factors, are constrained to zero (i.e., independent clusters model) (Marsh et al. 2014). As a result, CFA models often fail to provide an exact fit to the observed data and fail tests of model‐data consistency. As a result, these studies have generally relied on arbitrary cutoffs for approximate fit statistics (i.e., the Comparative Fit Index (CFI), Root Mean Square Error of Approximation (RMSEA), or Standardised Root Mean Square Residual (SRMR)), which provide only general guidance regarding model‐data correspondence and do not necessarily reflect adequate model fit (Kline 2023). The comorbidity between Externalising and Internalising problems, as well as the three other syndrome scales, Attention Problems, Thought Problems and Social Problems, can be represented by various CFA models, which converge on a common factor (Brown 2015). The convergence of psychopathology on a common factor is consistent across samples and measures in paediatric and adult samples, and has been labelled the ‘p factor.’ It has been hypothesised, amongst other theories, to represent a common underlying liability to all forms of mental illness. Despite the growing popularity and application of the p factor in developmental and adult psychopathology research, its psychometric ambiguities, ontological status and interpretation have been the subject of intense criticism and debate (Watts et al. 2019, 2024).

More recently, developmental psychopathology researchers have begun to fit the CBCL data to bifactor models (Sripada et al. 2021; Farahdel et al. 2021; Brislin et al. 2022; Hoffmann et al. 2022), which consist of a general factor, often inscribed as the *P*‐factor with loadings from all items or item parcels (i.e., subscales) to explain their common variance, coupled with orthogonal (i.e., uncorrelated) ‘specific factors’ or ‘group factors’, with loadings from subsets of conceptually‐ and empirically‐related indicators that capture theoretically‐consistent residual covariance amongst subsets of indicators not shared with the full set of remaining variables (e.g., Internalising and Externalising; Reise 2012). However, bifactor models have considerable conceptual and empirical problems, including providing superior fit to the data compared to competing models regardless of underlying covariance structure (i.e., high fit propensity) (Watts et al. 2024; Bornovalova et al. 2020). Additionally, bifactor models have high model complexity with many parameters that are more likely to provide a superior fit to the data compared to simpler models (i.e., parametric complexity) (Bornovalova et al. 2020). Thus, researchers have been encouraged to rely on alternative criteria for interpreting and potentially accepting bifactor models, such as substantive meaningfulness and interpretability (Bonifay et al. 2017).

Due to its widespread use, the CBCL has been incorporated as a primary measure of psychopathology in the landmark Adolescent Brain Cognitive Development (ABCD) study, which aims to track biological and behavioural development using genetic testing, neuroimaging and psychopathology assessments in >10,000 children and adolescents recruited from across the United States (Volkow et al. 2018). Accordingly, numerous studies have been published relying on either CBCL summed scale scores (Yu et al. 2021; Dash et al. 2023; Beyer et al. 2024) or latent models using approximate fit indices (Sripada et al. 2021; Brislin et al. 2022; Farahdel et al. 2021) to quantify dimensional variations in psychopathology within ABCD participants.

There are four limitations associated with this usage of the CBCL in the ABCD sample. First, efforts to apply the suggested bifactor structure to the ABCD dataset have demonstrated poor fit according to global fit indices, which quantify the discrepancy between observed data and model‐implied covariance matrices (Farahdel et al. 2021; Brislin et al. 2022), raising questions about the CBCL's suitability for the cohort. Second, the CBCL employs a limited response format (0 = Not true; 1 = Somewhat/Sometimes true; 2 = Very true/Often true), which can reduce its variance and thus attenuate correlations with other (e.g., neuroimaging, sociodemographic) variables (Goodwin and Leech 2006). Third, this response option format reduces psychometric precision, in that it limits sensitivity for measuring individual differences (Simms et al. 2019). Fourth, the CBCL was criterion‐keyed, with items selected that are representative of the presence of a clinically‐significant syndrome and which maximally differentiate clinically referred from non‐referred youth; it was not designed to measure continuous traits across the full population spectrum (Achenbach 2009). These design features can yield zero‐inflated distributions and limited information (i.e., reliability) at the low severity end of the latent trait continuum, similarly reducing associations with biological or other variables and further undermining utility for dimensional measurement (Simms et al. 2019; Greven et al. 2016).

Here, we conducted a multi‐step, multi‐method evaluation of these issues by assessing the psychometric properties of the CBCL in the baseline 8–11‐year‐old ABCD sample (*N* = 11,861) using approaches drawn from Classical Test Theory (CTT) and Item Response Theory (IRT) (Raykov and Marcoulides 2016).

## 2 Methods

### 2.1 Participants

Participants were 11,861 youth (6188 male; 6485 White, 1727 Black, 2150 Hispanic, 1499 other race/ethnicity) aged 8–11 years (*M* = 9.48, *SD* = 0.51) from the United States‐based ABCD study (release 5.1). This study recruited a demographically diverse community sample through probability sampling of US schools across 21 sites in the Northeast (*N* = 2005), Midwest (*N* = 2420), South (*N* = 3356) and West (*N* = 4080) (for more details, see Karcher and Barch 2021), where the majority of households reported; an annual income of at least $50,000 (7629), an education level of some college or higher (7228), and parents that were predominantly married (7983). The ABCD study is longitudinal, with data to date collected at the average ages 9, 11 and 13. Our analysis focused only on data collected at the baseline assessment (i.e., 8–11 years) between September 1, 2016 and November 15, 2018.

### 2.2 Data Analysis

#### 2.2.1 Overview

We used a multi‐stage approach to modelling the latent structure of the CBCL by examining the bi‐factor structure, and a unidimensional alternative in which the eight subscales only fit hierarchically onto a general P‐factor (Figure 2). Specifically, we investigated whether there was robust empirical evidence for (1) a coherent general factor, (i.e., evidence for a coherent Total Problems scale); and (2) the viability of distinct Internalising and Externalising scales once common variance across all eight subscales (i.e., attributable to the P‐factor) had been removed (Rodriguez et al. 2016). This approach follows from general recommendations for using bifactor models to assess scale psychometrics in psychopathology research (Bornovalova et al. 2020). This approach allowed us to determine whether the Internalising and Externalising scales contibute uniquely upon the eight subscales and Total Problems score, as represented by the P‐factor. The unidimensional model represented a more parsimonious model, in which only a P‐factor, reflecting a Total Problems score, faithfully captured the shared variance of the data.

First, we tested both models using CFA, which is the most widely used approach for latent modelling of psychometric data with an established or hypothesised latent structure (Brown 2015). Second, we tested both models with a more flexible Bayesian factor analytic approach (B‐CFA), which can better accommodate minor model misspecifications (Muthén and Asparouhov 2012). Third, to account for the possibility that latent subgroups within the sample may influence the latent architecture of CBCL scores, we fitted each model with Factor Mixture Models (FMMs) (Clark et al. 2013).

**FIGURE 1:**
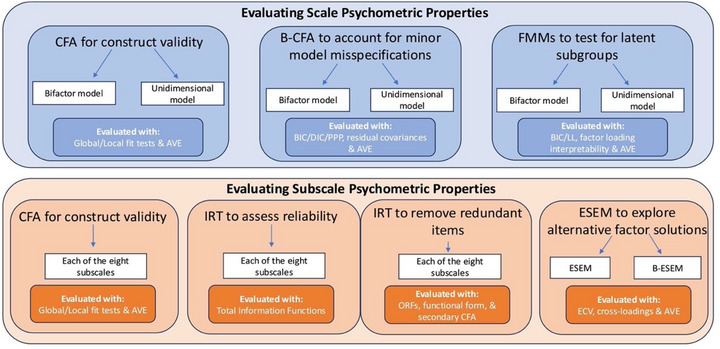
Workflow of each analysis employed, and the metrics used for evaluation. The hierarchical structure was examined using the sum scores from each subscale, while the subscale structure was examined at the item level for each subscale. AVE, average variance extracted, BIC, Bayesian information criterion; DIC, deviance information criterion; ECV, explained common variance; LL, log likelihood; ORFs, option response functions; PPP, posterior predictive *p* value.

We then assessed both the psychometric properties of each CBCL subscale by first applying CFA to each individual subscale to evaluate the (a) construct validity of the assumed unidimensional structure; and (b) the validity of each subscale to serve as a valid indicator (i.e., item parcel) in factor analytic models of the CBCL (Bandalos 2002; Marsh et al. 2013). Second, we used a graded response model (Toland 2014) within a unidimensional IRT framework to assess whether (a) each syndrome subscale measured the full latent trait continuum of each construct reliably; (b) items showed redundancy in their ability to discriminate between different levels of the latent trait (Reise and Waller 2009); and (c) we could refine each item pool to improve the construct's psychometric properties. Finally, Exploratory Structural Equation Models (ESEMs) (Marsh et al. 2014; Asparouhov and Muthén 2009) were conducted to examine whether alternative factor structures provided a better fit to the data in this sample compared to the empirically derived eight‐factor structure (Edelbrock and Achenbach 1980; Achenbach 2009). The full workflow is depicted in Figure 1.

All models were estimated in either R (v 4.3.3) or Mplus (v 8.11) (Muthén and Muthén 1998), and summaries of the results from Mplus were gathered using the *MplusAutomation* package (Hallquist and Wiley 2018). Code to reproduce the analysis found in this study can be found at (https://github.com/kanepav0002/CBCL_ABCD).

#### 2.2.2 Evaluating Scale Psychometric Properties

We first set out to evaluate the hierarchical structure of the CBCL. We did this using three different approaches (CFA, B‐CFA and FFM) as detailed in the following.

##### 2.2.2.1 Confirmatory Factor Analysis

CFA offers a powerful tool for testing whether a pre‐specified factor structure adequately explains patterns in the observed data (Kline 2014). CFA was used to evaluate both the commonly used bi‐factor structure of the CBCL, and a simpler unidimensional model. All CFAs were specified and fitted in R (v4.3.3) using the lavaan package (v0.6‐17; Rosseel 2012).

Model fit was evaluated based on global and local fit indices. Model fit was evaluated with a combination of a global fit test (i.e., exact fit test), global fit indices (i.e., approximate fit indices) and local fit. Specifically, model fit was first quantified by the Chi‐square (*χ* ^2^) test statistic, where *p* < 0.05 indicates poor fit (Kline 2014). However, since the *χ* ^2^ test statistic is over‐powered to identify minor and ignorable model misspecification in very large samples, such as the current one (Kline 2023), we also examined local fit in terms of the matrix of correlation residuals, which indicate how well the model reproduces the bivariate relationships between variables. Correlation residuals below <0.10 indicate an acceptable fit (Kline 2023; Goodboy and Kline 2017). Model comparisons can be made with respect to approximate fit indices: the Root Mean Square Error of Approximation (RMSEA) and its 90% confidence interval, the Comparative Fit Index (CFI) and the Standardised Root Mean Square Residual (SRMR), with smaller values of the RMESEA, and SRMR, and larger values of the CFI, indicating better fitting models (Kline 2023). Additionally, latent variables were required to have an Average Variance Extracted (AVE) of at least 0.5 (i.e., explaining an average of 50% of the variance across indicators) to confirm that sufficient variance was accounted for to justify retention (Cheung et al. 2024; Fornell and Larcker 1981). Reliability was evaluated using omega coefficients derived from the models (Flora 2020). Omega hierarchical (ω*_H_*) was used to estimate the proportion of variance in total scores attributable to the *P‐*factor, indexing the reliability of the total score as a measure of the common latent construct after accounting for specific factors. Omega hierarchical subscale (ω*_HS_*) was used to estimate the proportion of variance in subscale scores attributable uniquely to their corresponding specific factors, reflecting the reliability of subscale scores once general‐factor variance has been removed, where a criterion of ω*_HS_ >* 0.60 indicates acceptable reliability (Reise 2012; Rodriguez et al. 2016). For further information on model fit indices evaluated and estimators used, refer to Supplementary Methods 1.1.

##### 2.2.2.2 Bayesian Confirmatory Factor Analysis

CFA depends on stringent criteria to validate the underlying factor structure, assuming an independent clusters model in which items load on only one factor and indicators are also assumed to be locally independent with no residual associations after loading on their respective factors (Marsh et al. 2014). However, error covariances are conceptually and empirically reasonable in many cases due to contextual and conceptual overlap of variance (Brown 2015). Omission of true error covariances can result in distorted and unreliable model parameters (Kline 2023). B‐CFA offers greater flexibility to accommodate minor model parameters and relies on measures of model fit that are less sensitive than those employed in frequentist CFA (Muthén and Asparouhov 2012).

All B‐CFAs were implemented in Mplus using Markov Chain Monte Carlo (MCMC) estimation with the default Gibbs sampler (PX1). Models were executed with a minimum of 100,000 iterations and a maximum of 1,000,000 iterations, with convergence assessed using the Potential Scale Reduction (PSR) criterion set to 0.05 (equivalent to a PSR < 1.1), which is the default in Mplus. Competing models were evaluated with varying informative and uninformative prior means for thresholds (0.7, 0.5, 0.3), paired with a range of variances (1, 0.5, 0.1) using a normal prior distribution. Each combination of prior means and variances was further tested with a series of residual covariance priors (0.7, 0.5, 0.3, 0) using an improper inverse‐Wishart prior distribution (Muthén and Asparouhov 2012).

Model selection was guided by multiple fit indices, including the posterior predictive *p* value (PPP), Bayesian Information Criterion (BIC) and Deviance Information Criterion (DIC), which is a generalisation of BIC that accounts for model complexity by penalising based on the effective number of parameters (𝑝𝐷) (Spiegelhalter et al. 2002; Asparouhov et al. 2015). A PPP value closer to 0.5 was considered indicative of an excellent fit and was referred to first (Muthén and Asparouhov 2012), while lower BIC values signified better model fit (Asparouhov et al. 2015). Model comparison is facilitated by the DIC, where lower values identify the preferred model (Liu et al. 2022). The utility of the best‐fitting model was then assessed by examining the AVE and size and pattern of the residual covariances. Small residual covariances (<0.3; Muthén and Asparouhov 2012) suggest a good model fit, whereas large residual covariances indicated either poor fit or the potential presence of unmodelled factors (Stromeyer et al. 2015).

##### 2.2.2.3 Factor Mixture Models

It is possible that samples reflect heterogenous mixtures of homogenous subsamples (i.e., subtypes), which may directly relate to known characteristics (e.g., sex, ethnicity, age) or latent subgroups (Nylund‐Gibson and Choi. 2018). Traditional variable‐centred factor‐analytic methods often struggle with large, heterogeneous datasets because they assume a single, uniform factor structure across the entire sample. This assumption can obscure meaningful subgroup differences—such as distinct response patterns between sexes, cultural groups, or clinical subtypes—leading to biased or uninterpretable factor solutions (Lubke and Muthén 2005) and is a major consideration for studies of the ABCD sample, which was specifically designed to sample individuals from diverse sociodemographic and cultural backgrounds (Karcher and Barch 2021).

Following best‐practice guidelines (Lubke and Muthén 2005), we performed an initial Latent Class Analysis (LCA) on the full dataset to establish the upper bound of latent classes for subsequent FMM. To determine the optimal number of classes, we evaluated model fit using several criteria: (1) the Lo‐Mendell‐Rubin (LMR) statistic to assess whether a *k*‐class model provided significantly better fit than a (*k*‐1)‐class model; (2) the entropy (*E*) of the class classification, where values above 0.80 indicate clear class separation (Celeux and Soromenho 1996); (3) the Bayesian Information Criterion (BIC) to assess overall fit and model parsimony (Weller et al. 2020); and (4) a minimum class size threshold of 1% of the sample (Jung and Wickrama 2008), indicating that the smallest identified latent class made up more than 1% of the entire sample and was more likely to replicate. The final upper bound of number of classes was selected based on a significant LMR test, high *E* (≥0.8), and the lowest BIC relative to alternative models.

The upper bound was used to fit four different types of FMMs to both bi‐factor and unidimensional models across 1 to 𝑁 classes (See Supplementary Methods 1.2.). Additionally, each FMM was evaluated alongside a zero‐inflated variant (ZI‐FMM), where the loadings and means for the first latent class were constrained to zero. Models were estimated in Mplus with 20,000 initial starting values and 2000 for the final stages of optimisation. Model selection criteria included the lowest BIC, log‐likelihood (LL) values closest to zero, and *E* values nearest to one. The selected model was further evaluated for theoretically justified factor loadings and AVE across classes to ensure an adequate fit to the data.

#### 2.2.3 Evaluating the Psychometric Properties of the Subscales

In addition to testing the hierarchical organisation of the subscale structure assumed in the CBCL, we also examined the item‐level properties of the eight CBCL subscales using three approaches: CFA, IRT and ESEM. (1) CFA was used to test the construct validity of each assumed subscale factor and its suitability as an item parcel/factor indicator for factor analysis of the CBCL; (2) IRT was used to evaluate the reliability of each subscale across the latent trait continuum and assess items for redundancy) and (3) ESEM was used to see if alternative factor structures could represent the items used in these subscales better than the model fitted in the CFA.

##### 2.2.3.1 Confirmatory Factor Analysis

For each of the eight subscales, we conducted separate CFAs to evaluate whether their assumed unidimensional factor structures met established fit criteria for construct validity (Kline 2023). Each CFA was computed using the weighted least squares means and variance adjusted estimator (WLMSV), which is the optimal estimator for ordinal data (Li 2016). Model fit was evaluated using the same criteria and the same fit metrics were reported as the bifactor and unidimensional CFAs (Section 1 2.2.1).

##### 2.2.3.2 Unidimensional IRT Graded Response Models

IRT is a powerful approach for examining item‐level performance in relation to the underlying latent trait that a given measure is designed to capture (Toland 2014). It can be used to evaluate how well individual items perform in this task and to assess the ability of the measure to reliably capture the full spectrum of the latent trait (Thomas 2019). This method evaluates the properties of each item in relation to the trait it measures, allowing researchers to iteratively optimise the scale by retaining items that most accurately measure the underlying latent trait continuum (Toland 2014; Reise and Waller 2009).

IRT models were fitted to each subscale to evaluate their reliability across the latent trait continuum by inspecting their total information curves (TIF) (Toland 2014). A standard metric of marginal reliability or reliability at any point along the latent trait (*r*_xx_) can be calculated from total information as $\;r_{\mathrm{xx}}=1-\left(\frac{1}{I}\right)$, and summarises the precision of a range in each IRT model (Toland 2014; de Ayala 2009). Next, we examined the option response functions (ORFs) derived from the IRT models and considered two parameters: the slope parameter, *α*, measured in logistic metric, with higher values indicating items are more discriminative between different levels of the latent trait (i.e., provide more precise measurement); and the threshold parameter, *β*, measured in standardised units (i.e., *M* = 0, *SD* = 1), and corresponds to the location on the latent trait (θ) where the probability of endorsing the response category is 0.5 (Baker 2001; Reise et al. 2005) (Toland 2014). Graded Response Models (GRMs), which are appropriate for items with more than two response options, were fitted to each subscale using the *mirt* package (v1.42; Chalmers 2012) in R. Since zero‐inflated data can bias IRT models due to the resulting non‐normal distributions (Wall et al. 2015), zero‐inflated GRMs (ZI‐GRMs) were also fitted to each subscale. Additionally, LCA was performed on each subscale using Mplus, where the optimal class was identified using the same criteria as in Section 3.2.3. The zero‐inflated class from the optimal class solution was then manually removed from the data and GRMs were re‐estimated.

Once the GRMs were fitted, items were iteratively removed if they showed low discriminability, as indicated by low slope parameter values (*α* < 1.0) and high overlap with other items based on ORFs, and violations of functional form assumptions with 𝑆−*χ* ^2^ *p* < 0.01 (Toland 2014; Chen and Thissen 1997; Reise and Waller 2009). ORFs represent the probability of endorsing each response option on the latent trait continuum, with ideal items exhibiting ordered, non‐overlapping curves that show strong discrimination (De Ayala 2009). The functional form assumption evaluates whether the empirical response curves align with the expected function given by the GRM, with violations of this assumption suggesting item‐model misfit (Toland 2014; De Ayala 2009).

In each iteration, the poorest fitting item was removed, and the GRM was re‐run. Once items were pruned to a point where they no longer violated the above criteria, TIFs were re‐examined to determine whether there was a substantial change in reliability across the latent trait continuum associated with item removal. CFAs were then re‐estimated on the refined subscales to determine whether the revised scales satisfied criteria for construct validity through adequate global fit tests and indicators of local fit, as well as AVE.

##### 2.2.3.3 Exploratory Structural Equation Models

To evaluate whether alternative latent structures provided a better fit to the data, we conducted exploratory structural equation modelling (ESEM) and bifactor ESEM (B‐ESEM) in Mplus (Asparouhov and Muthén 2009; van Zyl and ten Klooster 2022). ESEM is a hybrid of EFA and CFA, enabling specification of the number of factors and generating fit statistics, but allowing item cross‐loadings (Marsh et al. 2014). For ESEM, we tested 1‐ to 10‐factor solutions using geomin rotation; for B‐ESEM, we applied bi‐geomin rotation with orthogonal factors. To ensure convergence, we set the minimum number of iterations to 10,000 for 1‐ to 5‐factor solutions and 50,000 for 5‐ to 10‐factor solutions, with a convergence criterion of 0.001.

Model fit was assessed using the same indices as in the CFAs (see Section 3.2.1). Unlike traditional bifactor CFA, bifactor ESEM does not permit calculation of the percentage of uncontaminated correlations—a metric used to assess whether the general factor dominates variance independently of group factors (i.e., without substantial confounding influence from cross‐loadings between general and group factors; Rodriguez et al. 2016a, 2016b), so bifactor solutions were chosen over non‐bifactor alternatives if the general factor accounted for >50% of the shared variance, as quantified by the omega hierarchical statistic (***ωH*** > 0.6; Rodriguez et al. 2016a).

## 3 Results

### 3.1 Hierarchical Structure

#### 3.1.1 Confirmatory Factor Analysis

**FIGURE 2:**
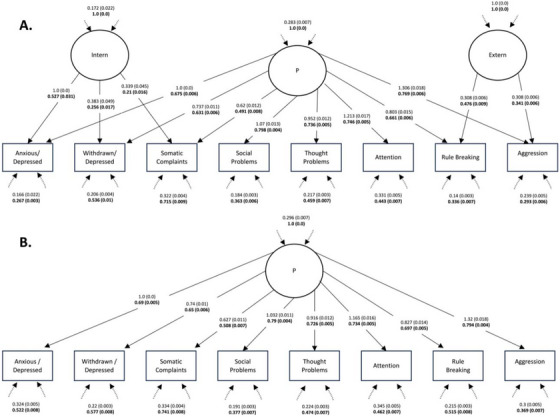
CFAs of the hierarchical structure of the CBCL. Standardised estimates are presented in bold, with standard errors shown in parentheses. Latent variables are depicted as circles, and observed variables are represented by squares. Error variances between the observed variables are shown in Tables S1 and S2. **(A)** CFA with a bifactor structure (*χ* ^2^ (16) = 1041.73, *p* < 0.001, CFI = 0.976, SRMR = 0.024, RMSEA (95% CI) = 0.074 (0.07, 0.077)). **(B)** CFA with a unidimensional factor structure (*χ* ^2^ (20) = 3063.66, *p* < 0.001, CFI = 0.930, SRMR = 0.041, RMSEA (95% CI) = 0.113 (0.11, 0.117)).

We conducted CFAs for both a bifactor and unidimensional structure of the CBCL (Figure 2). The bifactor model failed the exact fit test (*χ* ^2^ (16) = 1041.73, *p* < 0.001, *CFI* = 0.976, *SRMR* = 0.024, *RMSEA* (95% CI)* = *0.074 (0.07–0.077)). However, all 28 correlation residuals were below the threshold of ±0.1 (Table S1), indicating that the model sufficiently reproduced the bivariate relationships between the variables. However, the AVE for the Internalising, Externalising and general *P*‐factors were 0.129, 0.171 and 0.482, respectively, suggesting that each latent factor failed to account for sufficient variance to warrant retention. Furthermore, the reliability (ω*_H_* and ω*_HS_*) of the corresponding scales was 0.857, 0.028 and 0.0195. The explained common variance (0.841) and percentage of uncontaminated correlations (85.7%) indicated that multidimensionality could be ignored in favour of a unidimensional scale (i.e., Total Problems scale) (Rodriguez et al. 2016).

Global fit for the unidimensional model was worse than the bifactor model according to approximate fit indices (*χ* ^2^ (20) = 3063.662, *p* < 0.001, *CFI* = 0.930, *SRMR* = 0.041, *RMSEA* (95% CI)* = *0.113 (0.11–0.117)). However, only 3/28 correlation residuals fell outside the ±0.1 threshold (Table S2), These results highlight that the model showed only minor discrepancies in reproducing the observed variance‐covariance matrix. Additionally, the resulting *P*‐factor was associated with a near‐threshold AVE of 0.495. These results align with those of the bifactor model above, indicating that a unidimensional hierarchical CBCL scoring structure represents the data well. Furthermore, by allowing small error covariances between anxious depressed and social problems (Θδ = 0.112), and rule breaking and aggressive problems (Θδ = 0.117), to be freely estimated (consistent with theory and statistically significant after correction for multiple post hoc comparisons (Benjamini and Hochberg 1995)), a good fitting unidimensional model was obtained (*χ* ^2^ (18) = 1790.167, *p* < 0.001, *CFI* = 0.959, *SRMR* = 0.033, *RMSEA* (95% CI)* = *0.091 (0.088–0.095)), with no correlation residuals outside the ±0.1 threshold, and a sufficient *P‐*factor AVE of 0.503.

We additionally tested both a unidimensional and bifactor CFA from the item level, where subscales were specified from their respective items, and higher order Internalising, Externalising and general *P*‐factors were specified from the latent subscale factors. Unidimensional CFA showed poor global fit (*χ* ^2^ (5347) = 87,939.49, *p* < 0.001, *CFI* = 0.964, *SRMR* = 0.132, *RMSEA* (95% CI)* = *0.036 (0.036–0.036)), a *P‐*factor AVE of 0.71 and a high amount of residual correlations outside the ±0.1 threshold (986/5460), indicating that the model did not reproduce the observed covariance matrix. The bifactor CFA showed poor global fit (*χ* ^2^ (5343) = 78,061.57, *p* < 0.001, *CFI* = 0.968, *SRMR* = 0.129, *RMSEA* (95% CI)* = *0.034 (0.034–0.034)), an AVE of 0.68, 0.17 and 0.31 for the *P*, Internalising and Externalising factors, respectively, and a high amount of residual correlations (822/5460). Standardised loadings and residual correlations for both models can be found at (https://github.com/kanepav0002/CBCL_ABCD/tree/main/item_level_CFA_results)

Taken together, these results suggest that the subscales converge on a common dimension (p factor) that explains between 45% and 70% of their variance within a bifactor or common factor model. However, 85.7% of the reliable variance in Total Problems scores can be attributed to this general factor. Furthermore, Internalising and Externalising scales have almost no reliable variance once variance attributable to this general factor are partitioned out, indicating that they do not have construct validity.

#### 3.1.2 Bayesian Confirmatory Factor Analysis

**FIGURE 3:**
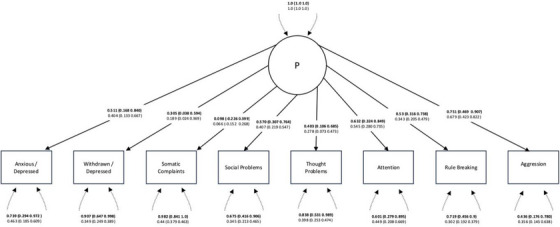
Bayesian CFA of the unidimensional CBCL structure (DIC = 83238.15, BIC = 83654.13, 𝑝𝐷 = 11.395, PPP = 0.501). Standardized estimates are presented in bold, with unstandardized estimates below in plain text. 95% credibility intervals with equal tails appear in brackets. Error covariances are shown in Table S6.

We next used B‐CFA to determine whether either the bifactor or unidimensional models could be fitted to the data under weaker constraints and assumptions than classical CFA. To evaluate the impact of prior distributions on model fit, separate models were tested using combinations of large, medium, and small variance and error priors (see Tables S4 and S5) (Tiego, Martin, et al. 2023). For the bifactor model, no combination of priors produced a positive 𝑝𝐷, indicating that the model specification was incompatible with the observed data structure (Spiegelhalter et al. 2002). For the unidimensional model, the combination of priors *N*(0.5,0.1) and IW(0.3,14) yielded a positive 𝑝𝐷, the lowest DIC, and a PPP value closest to 0.5. However, this solution showed no significant factor loadings. We therefore selected the next best‐fitting model, using *N*(0.7,0.1) and IW(0.7,14). This model (Figure 3) identified a general factor that explained a low proportion of variance (AVE = 0.17) and exhibited high residual covariances (Table S6), highlighting a misfit between the observed variance‐covariance matrix and the corresponding model‐estimated matrix (Stromeyer et al. 2015). Trace plots for all parameters in this model can be found at: https://github.com/kanepav0002/CBCL_ABCD/blob/main/traceplots.pdf

Together, these findings indicated that Bayesian CFA did not improve the fit of either the bifactor or unidimensional models.

#### 3.1.3 Factor Mixture Models

To examine whether there were heterogeneous mixtures or subgroups of participants within the ABCD sample, and potentially impacting on CBCL model fit, we performed FMMs on the log transformed summed scale scores.

As a first step, separate LCAs were fitted to solutions with 1–10 classes. The 7‐class solution was chosen as the best fitting model due to a significant LMR, a large reduction in BIC, high entropy (*E*) and a reasonable smallest class size (Table S7).

FMMs were then run across 1–7 classes for both the unidimensional and bifactor models (Tables S8 and S9). For the bifactor model, the seven class FMM‐1 showed the lowest BIC and LL while also showing high and a reasonable smallest class size (*BIC* = 158438.1, LL = −78989.24, *E* = 0.899, Smallest class = 590/11,862; 4.97%). However, this model showed a negative mean variance of the *P*‐factor, indicating an improper solution. Moreover, 51.6% of the factor loadings were statistically non‐significant and negative across classes, which is theoretically non‐sensical in the context of the CBCL. We therefore considered the next best fitting model (the 3‐class zero‐inflated FMM‐3; *BIC* = 163157.3, LL = −81344.12, *E* = 0.865, Smallest class *n* = 1083; 9.12%). This model showed negative residual variances of the Internalising factor, indicating that it also provided an improper solution.

For the unidimensional model, the FMM3 with a 5‐class solution revealed the best fit (*BIC* = 142065.2, LL = −7032.41, *E* = 1, Smallest class *n* = 632; 5.33%). However, this solution yielded low AVE across classes (AVE ranged from 0.303 to 0.379). These findings indicate that FMM cannot identify an adequate model of the data, suggesting that there is no clear evidence of subgroups embedded within the ABCD CBCL data for the bifactor and unidimensional CBCL models.

### 3.2 Subscale Psychometric Properties

#### 3.2.1 Confirmatory Factor Analysis

We further investigated the psychometric properties of the CBCL by evaluating the unidimensional structure of each subscale to assess if individual subscales showed evidence of construct validity. No subscale demonstrated adequate global fit, and only the Withdrawn/Depressed subscale exhibited sufficiently low and theoretically sensible correlation residuals, coupled with a sufficiently high AVE (>0.5), to justify retention (Table 1). These findings suggest that, except for the Withdrawn/Depressed subscale, the presumed structure of the CBCL subscales is not empirically supported in this sample.

**TABLE 1:** Fit statistics for each individual subscale of the CBCL.

	*X^2^*	*df*	*p*	*CFI*	*RMSEA* (95% CI)	*SRMR*	Residual correlations ±0.1	*AVE*
***Anxious depressed***	1539.703	65	<0.001	0.982	0.044 (0.042,0.046)	0.063	13/78	0.484
***Withdrawn depressed***	306.630	20	<0.001	0.988	0.035 (0.031,0.038)	0.048	1/28	0.507
***Somatic complaints***	451.913	44	<0.001	0.985	0.028 (0.026,0.03)	0.056	3/55	0.376
***Social problems***	1609.468	44	<0.001	0.962	0.055 (0.052,0.057)	0.078	11/55	0.434
***Thought problems***	1755.723	90	<0.001	0.946	0.04 (0.038,0.041)	0.145	38/105	0.406
***Attention problems***	1446.734	44	<0.001	0.993	0.052 (0.05,0.054)	0.062	6/55	0.551
***Rule breaking***	633.653	135	<0.001	0.989	0.018 (0.016,0.019)	0.325	56/153	0.438
***Aggressive behaviours***	3698.689	135	<0.001	0.986	0.047 (0.046,0.048)	0.064	22/153	0.581

*Note: χ^2^ = *Chi‐squared statistic; *df* = degrees of freedom; RMSEA = root mean square error of approximation; 95% CI = 95% confidence interval; CFI = comparative fit index; SRMR = standardised root mean square residual.

Furthermore, the results demonstrate that the subscales cannot be used as valid indicators for factor analytic models of the CBCL. More specifically, they cannot be used as item parcels (Bandalos 2002; Marsh et al. 2013), in which conceptually‐ and empirically‐related items are combined to form sum scores as raw data for factor analysis to represent high‐order structure and simplify model fit. This is because they do not conform to a unidimensional model, they have low convergent validity and fail to capture sufficient reliable variance to reflect an empirically cohesive construct, and they demonstrate large numbers of error covariances and thus sum scores contain multiple sources of extraneous variance. This combination of factors means that the sum scores fail to capture a sufficient single reliable and valid source of variance, and this could distort the variance‐covariance matrix for subsequent factor analysis.

#### 3.2.2 Item Response Theory

Although none of the subscales met criteria for unidimensionality and local dependence (as indicated by the significant *χ^2^* shown in our CFAs), all subscales met the requirements for essential unidimensionality through examination of their scree plots, indicating that a single common factor explained most of the item covariance, thereby satisfying the requirements for unidimensional IRT (Reise and Waller 2009) (Supplementary Methods 2.1; Figure S3).

We fitted GRMs and assessed the ability of each subscale to measure its target trait along the entire latent continuum. Examination of the TIFs indicated good measurement reliability (*r_xx_* = 0.856–0.955) at high levels of the latent trait (1.5–3 SDs above the mean), but poor reliability at lower levels, where *r_xx_* > 0.7 is the minimum recommended reliability cutoff for research use (Nunnally 1978). For example, reliability dropped to 0.01 at ∼1 SD below the mean (Figure 5A; Table S11; Figure S4). These results suggest that the subscale items effectively measure the higher end of each latent trait but fail to provide a reliable dimensional measure across the full severity spectrum.

**FIGURE 4:**
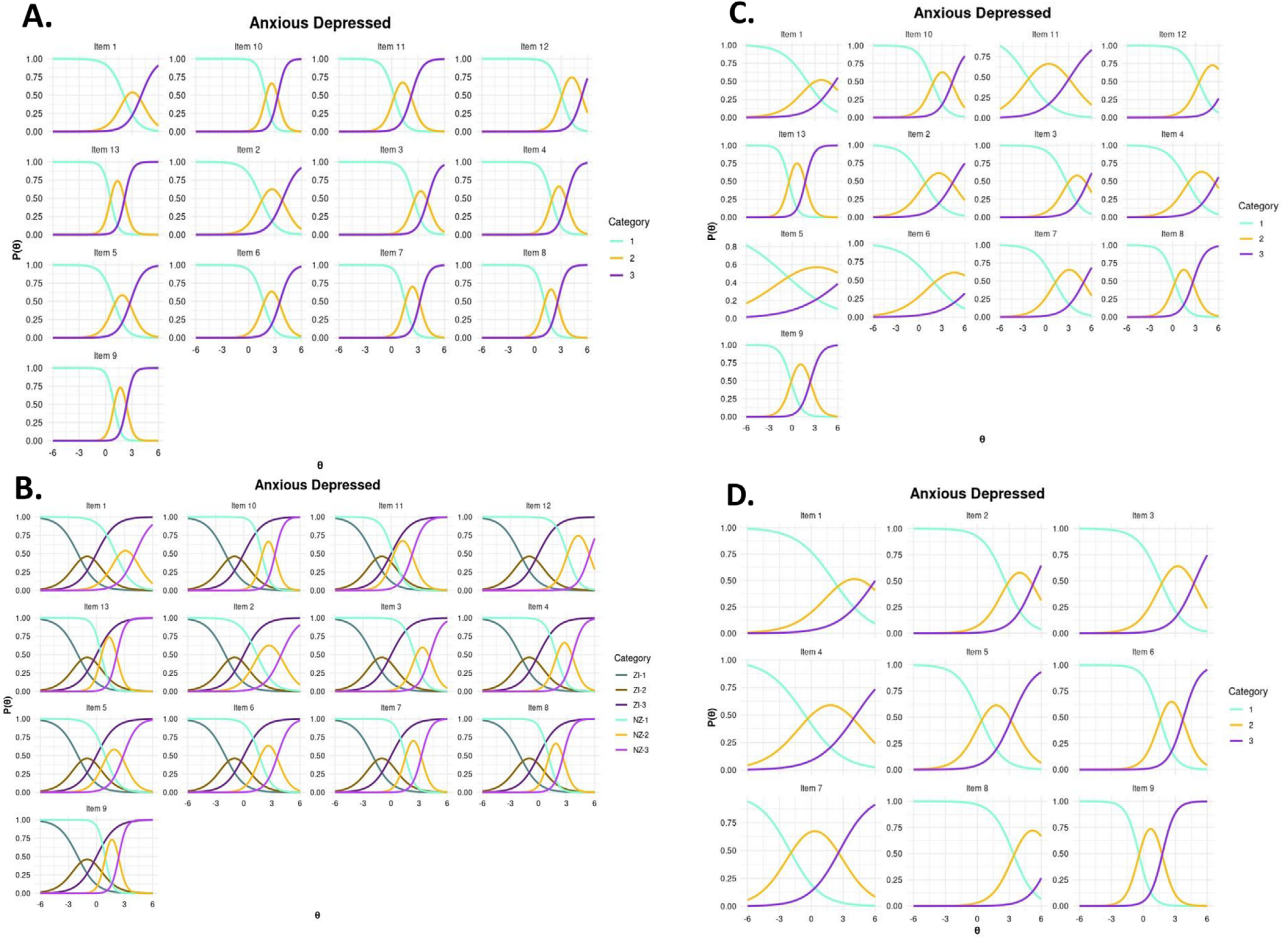
Option response functions for each item in the anxious depressed subscale. (**A)** GRM on the full sample. (**B)** Zero‐inflated GRMs run on the full sample. ZI labels denote the zero‐inflated component, while NZ denotes the non‐inflated component. (**C)** GRMs on a subsample after manual removal of the zero‐inflated component. (**D)** GRMs on the subsample after removal of items based on low discriminability (small slope parameter values), high overlap with other items, and/or violations of the functional form assumption. The level (β) of the latent trait (θ), measured in standardised units, is plotted on the *x*‐axis, while the probability of endorsing the latent trait is shown on the *y*‐axis. For plots of each subscale refer to Figures S6–S21.

Analysis of the ORFs revealed substantial overlap across items, indicating pronounced empirical redundancy. For all items, the slope parameters of individual response categories strongly overlapped, suggesting that the successive response options (i.e., 0 = Not True; 1 = Somewhat or sometimes True; 2 = Very True or Often True) failed to discriminate meaningfully between different levels of the latent trait. For example, across the Anxious Depressed subscale (0–3 *SDs* above the mean), the response categories provided nearly identical information, demonstrating a lack of discriminative capacity across this severity range (Figure 4A; Figures S6:9).

To address the possibility that these results were driven by biases resulting from zero‐inflated distributions in some scales, we fitted zero‐inflated GRMs (ZI‐GRMs) to each subscale (Figure 4B; Figures S10:13), but this analysis yielded similar results. Further inspection revealed that these models underestimated the size of the zero‐inflated class present in the data. To properly account for the zero‐inflation present in the data we conducted LCA on the subscales (Table S10) and manually removed the zero‐inflated component. We then re‐ran the GRMs on each non‐zero inflated subset (i.e., the subset of the data for which participants with very low scores were removed) (Figure 4C; Figure S14:17) and removed items in an iterative procedure if they showed low discriminability (i.e., weak differentiation between levels of the latent trait), high overlap with other items (suggesting redundancy), and/or violations of the functional form assumption (e.g., deviations from expected GRM curve) (Figure 4D; Figure S18:21).

**FIGURE 5:**
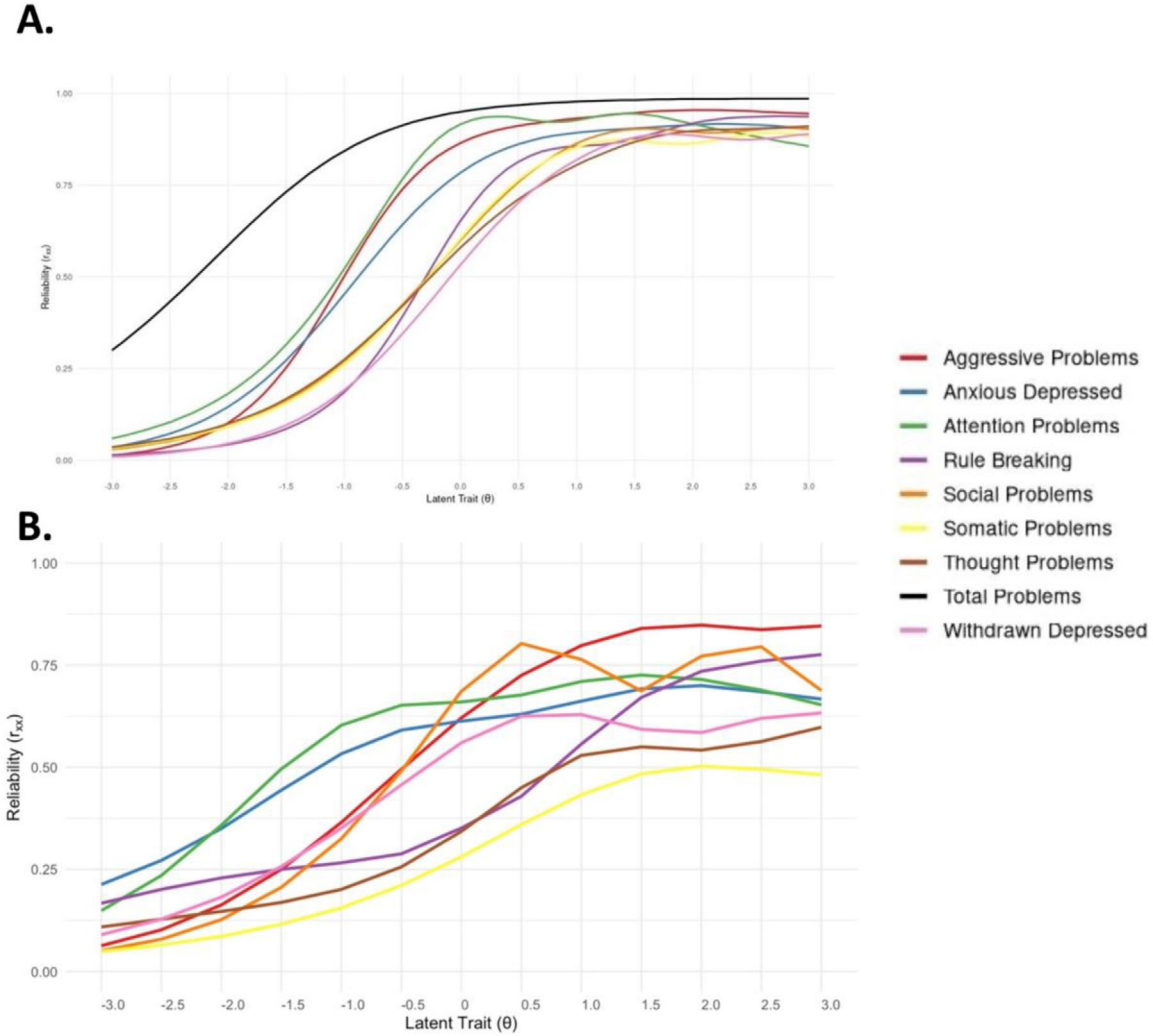
Reliability (*r*_xx_) across the latent trait (θ) continuum for: (**A**) each full subscale with all items, and the total problems scale (all items). (**B**) each subscale after item removal due to redundancies between items and violation of functional form.

Once the retained items no longer met any of the predefined exclusion criteria, TIFs were re‐estimated on the reduced item set with the zero‐inflated populations removed. These subsets showed higher estimates of reliability at the lower end of the latent trait (ranging from 0.049 to 0.213 at three standard deviations below the mean), but lower estimates of reliability at the higher end of the latent trait (i.e., 0.482–0.846 at three standard deviations above the mean) (Figure 5B; Figure S5). The adjusted Thought and Somatic complaints subscales did not meet criteria for acceptable reliability at any point along the latent trait continuum (Figure 5B; Table S12).

We additionally conducted CFAs on the reduced item set within the non‐zero‐inflated sample (Table 2) to assess if requirements for construct validity could be satisfied in our reduced item set measured in a subsample of participants with some non‐zero expression of each latent trait. This analysis showed that the reduced Thought Problems and Somatic Complaints subscales demonstrated acceptable global fit. Many other scales showed a low number of residual correlations, indicating acceptable local fit (Withdrawn/Depressed, Social Problems and Attention Problems). However, all subscales showed low AVE, indicating that they were not extracting sufficient variance to form coherent factors, rendering them unsuitable for further analyses.

**TABLE 2:** Fit statistics for each individual subscale of the CBCL after item pruning and removal of the zero‐inflated component.

	*X* ^2^	*p*	df	RMSEA (95% CI)	CFI	SRMR	Residual correlations ±0.1	Subjects retained	Items retained	AVE
***Anxious depressed***	352.579	<0.001	27	0.051 (0.047,0.056)	0.929	0.061	6/36	4591	9/13	0.252
***Withdrawn depressed***	130.752	<0.001	5	0.096 (0.082,0.111)	0.887	0.078	3/10	2731	5/8	0.27
***Social problems***	52.849	<0.001	5	0.052 (0.04,0.065)	0.985	0.05	1/10	3598	5/11	0.375
***Somatic complaints***	7.833	0.163	5	0.012 (0,0.028)	0.990	0.024	0/10	3869	5/11	0.188
***Thought problems***	21.821	0.009	9	0.021 (0.01,0.033)	0.963	0.059	3/15	3110	6/15	0.201
***Attention problems***	148.596	<0.001	14	0.036 (0.031,0.042)	0.983	0.037	1/21	7335	7/13	0.311
***Rule breaking***	147.881	<0.001	35	0.033 (0.028, 0.039)	0.921	0.082	14/45	2925	10/18	0.294
***Aggressive behaviours***	230.074	<0.001	27	0.035 (0.031, 0.039)	0.986	0.049	3/36	6073	9/18	0.433

*Note: χ^2^ *= Chi‐squared statistic; *df* = degrees of freedom; *RMSEA *= root mean square error of approximation; 95% CI = 95% confidence interval; CFI = comparative fit index; SRMR = standardised root mean square residual; AVE = average variance extracted.

#### 3.2.3 Exploratory Structural Equation Modelling

We next applied ESEM and B‐ESEM to evaluate whether an alternative factor structure provides a better fit. For both approaches, we tested solutions ranging from 1 to 10 factors. The standard ESEM analyses successfully converged (i.e., reached stable parameter estimates without computational errors) for 1–8 factor solutions, while B‐ESEM produced stable solutions for 1–6 factors plus a general factor (Tables S13 and S14). Solutions beyond these factor numbers failed to converge (did not reach stable estimates) or resulted in segmentation faults.

In the B‐ESEMs, no solution achieved an ***ωH*** exceeding 0.44, indicating that the general factor did not account for sufficient shared variance to justify its retention over traditional ESEM models (Rodriguez et al. 2016). For the ESEM solutions, models comprising 2–6 factors exhibited substantial cross‐loadings, suggesting poor discriminant validity (Marsh et al. 2014). Additionally, solutions with between 2 and 8 factors included at least one factor with low overall loadings, which is indicative of weak or unstable factors (Morin et al. 2016) (Figures S22 and S23).

## 4 Discussion

Our multi‐pronged approach to evaluating some of the psychometric properties of the CBCL in the ABCD cohort has revealed five key findings: (1) the CBCL's hierarchical structure converges on unidimensionality; (2) most subscales demonstrate insufficient construct validity; (3) all demonstrate poor measurement precision below the mean of the latent trait range; (4) IRT‐based item refinement fails to establish adequate construct validity for any subscale; and (5) ESEM fails to identify robust alternative factor structures. Our results indicate that the continued use of CBCL subscale scores and Internalising and Externalising composite scores for dimensional measurement may not yield reliable results and should be interpreted with caution.

### 4.1 The Hierarchical Structure of the CBCL

Our initial analyses evaluated whether the CBCL's bifactor structure modelling approach, commonly applied in ABCD studies (Sripada et al. 2021; Farahdel et al. 2021; Brislin et al. 2022), adequately represents the data in the ABCD sample. The results of CFA and bifactor statistics indicated that the 8 syndrome scales largely converged on a unidimensional model, with 86% of the reliable variance in the CBCL Total Problems scale was attributable to the *P*‐factor. Relatedly, the reliability of the Internalising and Externalising composite scales was just 0.028, and 0.019, respectively, once variance common to all eight syndrome scales was removed. Evidence of a robust *P*‐factor was further obtained from a unidimensionality model, but which failed to provide a good fit to the data. The more flexible B‐CFA failed to identify more adequate model fits. Importantly, FMMs failed to identify the presence of any homogenous subtypes embedded within the sample, and which may better account for hierarchical model fit of the CBCL.

These results should not be interpreted as providing evidence for the validity and utility of the CBCL Total Problems composite scale score in the ABCD baseline wave data for three reasons. First, the CBCL subscales are largely invalid as indicators of the higher‐order *P*‐factor in factor analytic models and likely result in distorted parameter estimates (Marsh et al. 2013). Second, even when modelled as single items, we found that the CBCL failed to coalesce as a coherent general factor capturing most of the item variance. Third, even when combined at item level, the CBCL Total Problems score exhibits poor reliability below the mean of the latent trait in the ABCD study data (Tiego, Martin, et al. 2023).

Numerous studies analysing the ABCD dataset use higher‐order CBCL structures to assess internalising, externalising and general psychopathology (Yu et al. 2021; Dash et al. 2023; Beyer et al. 2024; Sripada et al. 2021; Farahdel et al. 2021; Brislin et al. 2022). These constructs are measured either by summing scores on subscales without first demonstrating construct validity (e.g., by using global or local fit tests in CFA), or by evaluating the fits of factor models with approximate fit indices (e.g., RMSEA, CFI, SRMR) that are known to be inadequate for discriminating adequate from poor model fits (Kline 2023). Our analyses demonstrate the consequences of relying on approximate fit indices: even CFA models with ostensibly “good” fits according to widely applied cutoffs for approximate fit indices (Hu and Bentler 1995) can yield unsatisfactory solutions, in which Internalising and Externalising factors account for merely 10%–20% of indicator variance, which is well below established standards for defining coherent and interpretable latent constructs (Fornell and Larcker 1981), as well as less than 3% reliable scale variance. The resulting Internalising and Externalising factors within this sample are therefore likely to reflect a combination of largely common variance in total problems in combination with some measurement error, rather than meaningful variance in unique psychopathology.

In our approach, we used a combination of global and local fit tests, along with an AVE threshold to determine model fit, rather than relying on commonly used approximate fit indices which are insufficient for determining model‐data consistency in CFA (Kline 2023). Using these metrics, we showed that while bifactor models pass local fit tests, the low ω*_HS_* and AVE of the Internalising and Externalising factors indicate the presence of insubstantial constructs. Among all models evaluated, a unidimensional *P*‐factor CFA demonstrated the closest approximation to adequate fit. This solution showed a small amount of local misfit (only 3/28 significant residual correlations, less than θδ = 0.2) and an AVE of 0.495. This is slightly below the conventional 0.5 threshold, which some have argued is overly stringent (Cheung et al. 2024). The AVE = 0.5 threshold should therefore be viewed as a ‘rule of thumb’ rather than a definitive statistical criterion. Moreover, allowing the two theoretically‐ and empirically‐consistent residual correlations to covary resulted in a reproduction of the variance‐covariance matrix, with a sufficient AVE of 0.503 and no correlation residuals above 0.10. This model was thus deemed an acceptable representation of the data. Acceptance of this model stands in stark contrast to the original conceptual framework of the CBCL, in which Internalising and Externalising are viewed as core dimensions of child psychopathology. Additionally, the model's utility depends entirely on the assumption that subscale indicators are reliably measuring their respective constructs. Our analysis of the CBCL's subscale structure clearly shows that this is not the case in the ABCD sample. One plausible explanation for this is due the low level of symptom endorsement characteristic of this community sample (i.e., zero‐inflation). In zero‐inflated data, the restricted variability in item responses substantially attenuates the covariance matrix from which latent factors are estimated, effectively collapsing meaningful variance toward zero (Christensen et al. 2022). Under these conditions, the models may fail to recover the substantive variance associated with Internalising and Externalising domains, limiting their recoverability in latent variable models despite their conceptual relevance.

### 4.2 Psychometric Properties of the CBCL Subscales

Our second analytic phase evaluated the psychometric properties of all eight CBCL subscales. For the subscales to be reliable and valid measures of their target latent construct, as well as serving as valid indicators for factor analytic modelling, they should meet the criteria for convergent validity; additionally, they should provide reliable measurement across the entire latent trait continuum (Reise and Waller 2009). Our CFA revealed that only the Withdrawn/Depressed subscale demonstrates adequate convergent validity in this sample. IRT analyses further identified two critical limitations: (1) all subscales show poor reliability below the mean of the latent trait continuum; and (2) all the items of all subscales have a limited ability to discriminate between different levels of the latent trait. It is important to note that scales/subscales can demonstrate high internal consistency reliability (i.e., Cronbach's *α*) in the presence of multidimensionality within a CTT framework even when measurement precision beyond certain points of the latent trait continuum are low (Rodriguez et al. 2016).

Consider the Withdrawn/Depressed subscale of the CBCL, for which 56% of people score at or below the latent trait mean. Our analysis indicates that the reliability of scores for these participants is lower than 0.533 (where 0.7 indicates acceptable reliability) (de Ayala 2009), meaning that the scores for these individuals in large part reflect measurement error rather than accurate measurement of the target construct. Even at the higher end of the latent trait continuum, where reliability is acceptable for all subscales, our analysis indicates that most items are unable to discriminate between different levels of the latent trait, meaning that each subscale is offering a limited ability to distinguish between those who present with different levels of trait severity (e.g., between people with moderate vs. severe levels of the latent trait). Refinement of these subscales through item removal within a non‐zero inflated subsample did improve discriminability, but low reliability at the less severe end of the latent trait continuum remained a problem and the refined subscales did not meet standards for convergent validity highlighted through our secondary CFAs.

These findings should be unsurprising when one considers the original design of the CBCL, which was based on criterion‐keying, meaning that items were chosen to identify children and adolescents with clinically significant emotional and behavioural problems warranting referral for further evaluation (Edelbrock and Achenbach 1980; Achenbach 2009). As such, its primary function was to distinguish between referred and non‐referred youth and not to assess continuous variation in the underlying latent traits. The item‐level ORFs we observe in our analysis support this strong discriminative capacity around a clinical threshold. However, this comes at the cost of reduced precision across the broader latent trait continuum and a limited ability to differentiate among degrees of symptom severity, particularly at the lower end. Our findings thus indicate that researchers should carefully consider using the CBCL in population‐scale research as a dimensional measure of psychopathology in association studies with various psychosocial and/or biological variables.

### 4.3 Alternative Factor Structures

We employed ESEM to investigate whether alternative factor structures might better represent the item‐set in this sample. All ESEM solutions exhibited problematic features, including excessive cross‐loadings and weakly defined factors with low primary loadings (<0.40), reflecting poor discriminant validity and unstable dimensional structure. These results suggest that the CBCL items fail to coalesce into psychometrically distinct dimensions within the ABCD sample.

Prior studies have also sought to find alternative factor structures for the CBCL in the ABCD data. Michelini et al. (2019) applied bass‐ackwards modelling (Forbes 2024), an exploratory data‐driven dimension reduction technique akin to EFA, and proposed a six‐factor structure (Internalising, Externalising, Detachment, Somatoform, Neurodevelopmental and a general *P*‐factor), although subsequent validation showed poor global fit and reliance on approximate fit indices (Stewart et al. 2024), which is insufficient for establishing trustworthy models (Kline 2023). Additionally, this study used a reduced item set which undermines the established content validity of the CBCL. The items were carefully chosen to attain full coverage of the problem domain (Achenbach 2009). Other exploratory factor analyses in the ABCD sample have produced different alternative structures (Vedechkina et al. 2024; Clark et al. 2021), but follow‐up CFAs of the identified structures have all relied on approximate fit indices to justify their solutions.

Although the use of exploratory methods within this sample may help to find a latent structure of psychopathology within the ABCD data, the application of such methods risks yielding sample‐specific solutions with limited generalisability (Swami et al. 2023). It is therefore critical that any such exploratory models in the ABCD data are thoroughly evaluated for both construct validity and latent trait reliability to ensure that they support reliable and valid inferences about dimensional psychopathology, as well as yield unbiased and reproducible effect sizes in brain‐behaviour association studies (Tiego, Martin, et al. 2023).

### 4.4 Limitations

One approach not considered in the current study is the integration of the CBCL with other measures to enhance the assessment of psychopathology. For instance, Jacobs et al. (2024) has successfully combined multiple child‐ and adult‐report measures (including the CBCL) in the ABCD dataset to derive a higher‐order factor structure of psychopathology. Such an approach could improve the psychometric validity and reliability of the CBCL in this sample by supplementing its phenotypic depth. Tiego, Martin, et al. (2023) also demonstrated that combining the CBCL's attention problem scale with effortful control items from the Early Adolescent Temperament Questionnaire improved reliability across the latent trait continuum. Further work should explore whether combining subscales within the CBCL's domains enhances its reliability, and thus validity, in this sample.

In this analysis, we used a combination of AVE, a test of convergent validity and an indicator of local fit to evaluate the construct validity of the higher‐order CBCL constructs, along with ORFs and TIFs to assess the reliability of each CBCL subscale. It is important to note that this represents just one approach to evaluating validity and reliability in psychometric research. Other commonly used methods include multi‐trait multi‐method analysis for assessing construct validity (Campbell and Fiske 1959), or measurement invariance testing to examine construct stability across groups and time (Sterner et al. 2025). The indices selected for the present study were chosen to enable a rigorous and conservative evaluation of the CBCL's psychometric properties cross‐sectionally from one set of informants, and consistent with current best practices in psychometric assessment (Kline 2023). Nevertheless, alternative indices may yield more favourable model fits than those reported here.

We evaluated the CBCL at the baseline time point (the most widely used time point in ABCD research; e.g., Yu et al. 2021; Dash et al. 2023; Beyer et al. 2024), but the ABCD also contains later adolescent assessments. The potential for increasing psychopathological burden over time in this sample may change the psychometric properties of the instrument. Our findings should thus be interpreted as specific to the baseline age range (8–11 years). Future studies should examine the measure's reliability and validity across subsequent time points to determine whether later assessments yield more robust psychometrics. However, our findings in the baseline sample have implications for longitudinal studies.

Our hierarchical‐level analyses were conducted using summed subscale scores rather than item‐level indicators. Although this approach aligns with how the CBCL is most commonly operationalised in the ABCD literature and reflects typical applied scoring practices, it necessarily constrains the modelling of item‐level cross‐loadings and may influence comparisons between higher‐order and bifactor specifications. Although we provide an item‐level sensitivity analysis of our traditional CFA, such analyses were not computationally feasible in the Bayesian CFA and Factor Mixture Models given the substantial convergence time and complexity at this scale. Future work leveraging greater computational resources or alternative estimation strategies should evaluate whether the present conclusions replicate at the full item level.

Finally, it is important to acknowledge that the CBCL, like all questionnaire‐based instruments, provides a structured and simplified measurement representation of phenomena that are inherently non‐linear, dynamic and context‐dependent (Wackers and Schille‐Rognmo 2022). The constructs assessed by the CBCL should therefore be understood as higher‐level abstractions of heterogeneous psychological processes rather than direct reflections of the underlying complexity of psychopathology. To evaluate how well this measurement representation holds, we intentionally used both linear (factor analysis) non‐linear (IRT), and hybrid (FMM) approaches, allowing us to assess the robustness of the CBCL's structure and precision across different assumptions of the most popular models used in the literature. Although the convergence of findings across these methods strengthens the credibility of our conclusions, these approaches necessarily remain simplifications and cannot fully characterise the dynamic and interactive processes that generate psychopathology. Future work incorporating dynamic, longitudinal, or network‐based methods (Briganti et al. 2024) may help model these non‐linear processes more directly and further clarify how CBCL constructs relate to the underlying architecture of psychopathology.

## 5 Conclusions

This comprehensive psychometric evaluation of the CBCL challenges its utility as a dimensional measure of psychopathology in the ABCD cohort within a 9‐ to 10‐year‐old age range. Using a multi‐stage, multi‐method approach we demonstrated that the CBCL's hypothesised structures fail to adequately capture psychopathology in this sample, with poor model fit, low variance extracted and implausible parameter estimates. Additionally, we showed that most subscales lack construct validity and exhibit unreliable measurement precision below the mean of the latent trait continuum, where population‐scale research requires measurement accuracy. Researchers using the ABCD dataset should exercise caution when interpreting CBCL‐derived constructs and consider alternative instruments better suited to the dimensional assessment of psychopathology.

## Funding

**Alex Fornito** was supported by the National Health and Medical Research Council (ID: 1197431) and Australian Research Council (ID: FL220100184). **Jeggan Tiego** was supported by the National Health and Medical Research Council Investigator Grant 2033976. This research was supported by Monash eResearch capabilities, including M3 (Massive).

## Conflicts of Interest

None of the authors have a conflict of interest to disclose.

## Supporting information




**Supporting File 1**: desc70216‐sup‐0001‐SuppMat.docx

## Data Availability

The data used in this study were obtained from the ABCD Study, release 5.1, under the terms of a data use agreement. Raw data cannot be shared directly by the authors but are accessible through application on the ABCD website: https://abcdstudy.org/
