# Supplementary material for: Assessing the Psychometric Properties of the Child Behaviour Checklist in the ABCD Study
Source: Dev Sci. 2026 May 10;29:e70216. doi: 10.1111/desc.70216 (PMC13158641; doi:10.1111/desc.70216)
Supplement: Supplementary file 1 — Supporting File 1: desc70216‐sup‐0001‐SuppMat.docx [file DESC-29-e70216-s001.docx]

***Assessing the Psychometric Properties of the Child Behavior Checklist in the ABCD Study* – Supplementary Materials**

**Supplementary Methods**

- 1. ***Modelling Hierachical Structure***
  2. *CFA*

Prior to model estimation, the summed scores for each of the eight scales of the CBCL underwent a logarithmic transformation to address positive skew present in the data (*Supplementary Figure 1*). Summed scores were used for the hierarchical models, as this approach aligns with the most widely applied bifactor modelling practices in the current literature (e.g., Sripada et al., 2021; Brislin et al., 2021; Farahdel et al., 2021). Item-level hierarchical models were not considered due to the risk of unstable estimates and convergence issues associated with excessive indicators per factor (Kline, 2023; Little & Card, 2024).

CFA models were estimated using the robust maximum likelihood estimator, and lavaan was set to mimic default settings from Mplus (*mimic=Mplus*). ~~In this analysis, we did not freely estimate error covariances in these models, as each subscale reflects distinct constructs without theoretical justification for correlated errors (Kline, 2023). Our subsequent evaluation of B-CFAs was partly conducted to account for the possibility of minor covariances between subscales (see below).~~

CFAs were reported with the RMESEA (with 95% confidence intervals), the CFI, and the SRMR, with lower values of the RMSEA and SRMR, and higher values of the CFI, indicating better model fit. To facilitate replication efforts, we also report the number of iterations required for convergence, chosen based on the nonlinear minimisation subject to box constraints (NLMINB) optimisation method (Gay, 1990). Convergence criteria were set at the default lavaan value of 1e^-9^ (Appelbaum et al., 2018).

- 1. *Factor Mixture Models*

FMMs provide a framework for identifying latent subgroups (or mixtures) within a factor model by combining elements of Latent Class Analysis (LCA) (to identify subgroups) with common factor modelling to account for heterogeneity in responses that can occur in large datasets (Lubke & Muthén, 2005). The fit of FMMs is tested incrementally. The first step begins with the simplest model, which allows only the latent factor means to vary across classes (FMM-1); the second step relaxes model constraints to permit between-class differences in the factor covariance matrix (i.e. the variances of factors, and the covariances between then) (FMM-2); steps 3 and 4 then allow variability in item thresholds (i.e., the observed response thresholds, reflecting differences in item endorsement probabilities across classes) (FMM-3) and factor loadings (FMM-4), respectively (Clark et al., 2013).

Successive relaxation of parameter constraints has implications for model class comparisons and model interpretation (Lubke & Muthén, 2005). For example, models with relaxed item thresholds (FMM-3) cannot be tested for equivalence of means between classes, as differences between classes arise due to differential item response patterns, rather than differences in factor means. Similarly, relaxed factor loadings (FMM-4) indicate that substantively different constructs are being measured between classes; i.e., that factors between classes are composed of distinct constructs (Clark et al., 2013). FMMs are particularly effective at distinguishing between individuals that comprise the zero-inflated and other components of a distribution, which is a common problem in psychometric assessments of non-clinical samples (Magnus & Thissen, 2017).

- 1. ***Modelling Hierachical Structure***

*2.1. IRT Graded response models*

Prior to fitting an IRT model, subscales must meet the assumption of essential unidimensionality, as each model is designed to measure a single latent factor (Cattell, 1966). These assumptions are commonly evaluated using CFA, in which the criteria for unidimensionality and local independence can be met from a model that passes global or local fit tests. Essential unidimensionality and essential independence of items are weaker assumptions than unidimensionality and local independence, but still allow for consistent estimation within a unidimensional IRT model (Strout, 1990). Essential unidimensionality can be assessed using an EFA framework. Specifically, if the eigenvalue of the first factor is substantially larger than the eigenvalues of the successive factors, the covariance of the items is largely explained by a single common factor. We conducted a principal components analysis (PCA) on all items from each subscale, and calculated the variance explained by each eigenvalue of the covariance matrix. We then examined the scree plot to determine whether there was a deflection point following extraction of the first factor, indicating that the first eigenvalue explained substantially more variance than subsequent values (Reise et al., 2000).

**Supplementary Results**

**
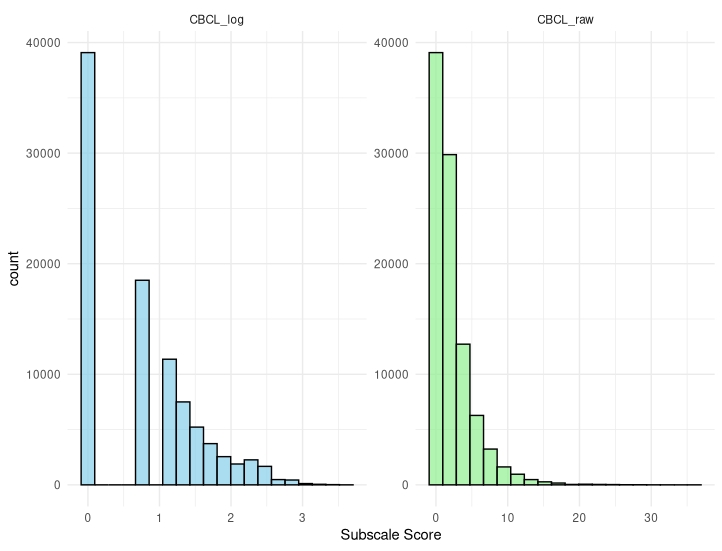
**

*Supplementary Figure 1.* Individual participant scores across each of the eight CBCL subscales. The green distribution shows original scores, while the blue distribution shows the log transformed scores used to address the positive skew.

1. ***CFAs***

*Supplementary Table 1.* Matrix of correlation residuals from the bi-factor CFA (Made up of Internalising, Externalising and General P-Factor) presented in section 1.1 of the main text.

|  | **Anxious Depressed** | **Withdrawn Depressed** | **Social Problems** | **Somatic Complaints** | **Thought Problems** | **Attention Problems** | **Rule Breaking** | **Aggression** |
| --- | --- | --- | --- | --- | --- | --- | --- | --- |
| **Anxious Depressed** |  |  |  |  |  |  |  |  |
| **Withdrawn Depressed** | <.001 |  |  |  |  |  |  |  |
| **Social Problems** | 0.031 | 0.039 |  |  |  |  |  |  |
| **Somatic Complaints** | <.001 | <.001 | 0.003 |  |  |  |  |  |
| **Thought Problems** | 0.044 | 0.005 | -0.035 | 0.054 |  |  |  |  |
| **Attention Problems** | -0.054 | -0.039 | -0.008 | -0.045 | 0.028 |  |  |  |
| **Rule Breaking** | -0.07 | -0.012 | 0.011 | -0.023 | -0.012 | 0.047 |  |  |
| **Aggression** | <.001 | -0.012 | <.001 | -0.001 | -0.02 | 0.025 | <.001 |  |

*Supplementary Table 2.* Matrix of correlation residuals from the unidimensional CFA (only including a general P factor) presented in section 1.1 of the main text.

|  | **Anxious Depressed** | **Withdrawn Depressed** | **Social Problems** | **Somatic Complaints** | **Thought Problems** | **Attention Problems** | **Rule Breaking** | **Aggression** |
| --- | --- | --- | --- | --- | --- | --- | --- | --- |
| **Anxious Depressed** | 0 |  |  |  |  |  |  |  |
| **Withdrawn Depressed** | <.001 | 0 |  |  |  |  |  |  |
| **Social Problems** | 0.112 | 0.03 | 0 |  |  |  |  |  |
| **Somatic Complaints** | 0.024 | 0.033 | -0.007 | 0 |  |  |  |  |
| **Thought Problems** | 0.09 | -0.003 | -0.021 | 0.046 | 0 |  |  |  |
| **Attention Problems** | -0.057 | -0.045 | 0.009 | -0.052 | 0.045 | 0 |  |  |
| **Rule Breaking** | -0.105 | -0.047 | -0.011 | -0.053 | -0.031 | 0.03 | 0 |  |
| **Aggression** | -0.03 | -0.044 | -0.014 | -0.028 | -0.031 | 0.016 | 0.117 | 0 |

*Note.* Residuals over the absolute value of 0.1. are highlighted in the red, indicating areas of local misfit.

*Supplementary Table 3.* Matrix of correlation residuals from a correlated two-factor model (comprising Internalising & Externalising).

|  | **Anxious Depressed** | **Withdrawn Depressed** | **Somatic Complaints** | **Rule Breaking** | **Aggression** |
| --- | --- | --- | --- | --- | --- |
| **Anxious Depressed** | 0 |  |  |  |  |
| **Withdrawn Depressed** | 0.004 | 0 |  |  |  |
| **Somatic Complaints** | 0.018 | -0.032 | 0 |  |  |
| **Rule Breaking** | -0.083 | -0.022 | -0.024 | 0 |  |
| **Aggression** | 0.148 | 0.126 | 0.113 | 0.049 | 0 |

*Note.* Residuals over the absolute value of 0.1. are highlighted in the red, indicating areas of local misfit.

**
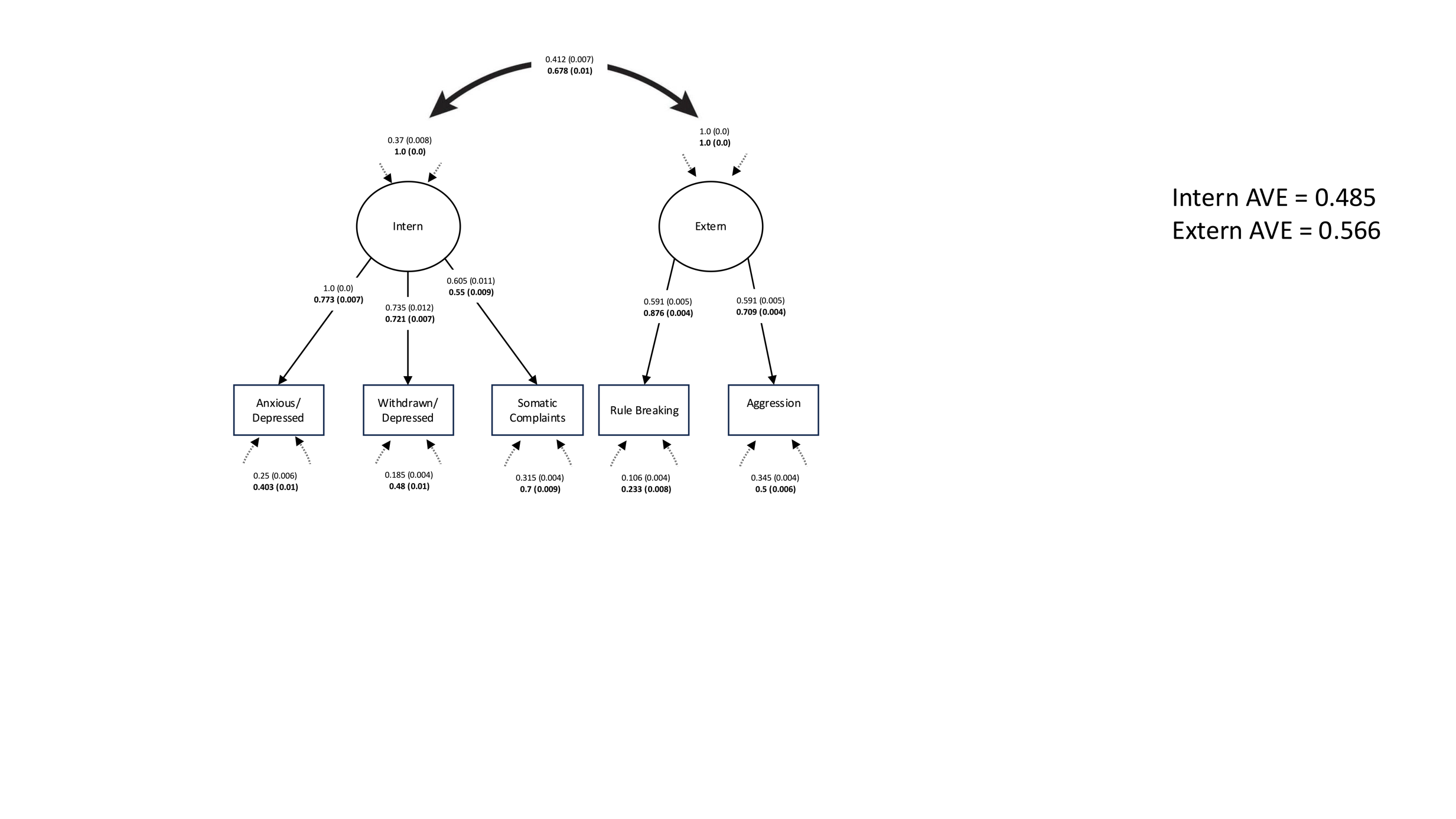
**

*Supplementary Figure 2.* CFA of a correlated two-factor model. Standardized estimates are presented in bold, with standard errors shown in parentheses. Latent variables are depicted as circles, and observed variables are represented by squares. (χ² (5) = 1636.41, *p* < .001, *CFI* = 0.916, *SRMR* = 0.072, *RMSEA(95% CI) =* 0.166 (0.159:0.173))*.*

1. ***Bayesian CFAs***

*Supplementary Table 4.* Bayesian CFA model fit indices for the bi-factor model.

| λ | λ error | Residual Covariance | *q* | DIC | BIC | PPP | PPPP | CFI | 𝑝𝐷 | LB χ*^2^* | UB χ*^2^* |
| --- | --- | --- | --- | --- | --- | --- | --- | --- | --- | --- | --- |
| **0.7** | **1** | **0.7** | 57 | 80237.42 | 84610.77 | 0.499 | n/a | 1 | -2982.85 | -26.159 | 26.865 |
|  |  | **0.5** | 57 | 81250.68 | 85014.86 | 0.5 | n/a | 1 | -1969.56 | -26.084 | 26.041 |
|  |  | **0.3** | 57 | 81082.39 | 85216.01 | 0.494 | n/a | 1 | -2137.9 | -25.786 | 26.46 |
|  |  | **0** | 57 | 82397.25 | 84306.01 | 0.502 | n/a | 1 | -822.885 | -25.903 | 25.943 |
|  | **0.5** | **0.7** | 57 | 82366.12 | 84145.8 | 0.502 | 1 | 1 | -854.065 | -26.007 | 25.94 |
|  |  | **0.5** | 57 | 82144.95 | 84040.43 | 0.498 | 1 | 1 | -1075.3 | -25.975 | 26.091 |
|  |  | **0.3** | 57 | 82348.48 | 84201.71 | 0.496 | 1 | 1 | -871.811 | -25.826 | 26.213 |
|  |  | **0** | 57 | 82478 | 84142.95 | 0.499 | 1 | 1 | -742.112 | -26.118 | 26.13 |
|  | **0.1** | **0.7** | 57 | 83062.07 | 83844.07 | 0.497 | 0.934 | 1 | -158.275 | -25.933 | 26.339 |
|  |  | **0.5** | 57 | 83053.89 | 83876.51 | 0.496 | 0.934 | 1 | -166.491 | -25.765 | 25.969 |
|  |  | **0.3** | 57 | 83013.79 | 83821.55 | 0.496 | 0.934 | 1 | -116.532 | -26.044 | 25.798 |
|  |  | **0** | 57 | 83129.41 | 83785.76 | 0.498 | 0.934 | 1 | -90.833 | -26.251 | 26.519 |
| **0.5** | **1** | **0.7** | 57 | 81236 | 84330.12 | 0.502 | n/a | 1 | -1984.18 | -26.206 | 26.141 |
|  |  | **0.5** | 57 | 81525.55 | 85180.01 | 0.499 | n/a | 1 | -1694.72 | -26.112 | 26.251 |
|  |  | **0.3** | 57 | 81547.5 | 84533.89 | 0.497 | n/a | 1 | -1672.74 | -25.9 | 26.17 |
|  |  | **0** | 57 | 82459.28 | 84182.66 | 0.499 | n/a | 1 | -760.89 | -26.096 | 25.94 |
|  | **0.5** | **0.7** | 57 | 81973.68 | 83955.35 | 0.5 | 1 | 1 | -1246.46 | -26.101 | 25.965 |
|  |  | **0.5** | 57 | 80917.51 | 84322.05 | 0.499 | 1 | 1 | -2302.76 | -26.135 | 26.361 |
|  |  | **0.3** | 57 | 81469.33 | 84297.88 | 0.499 | 1 | 1 | -1750.77 | -26.14 | 26.088 |
|  |  | **0** | 57 | 82472.04 | 84425.69 | 0.499 | 1 | 1 | -748.164 | -26.039 | 26.261 |
|  | **0.1** | **0.7** | 57 | 82772.45 | 84041.79 | 0.496 | 0.996 | 1 | -447.872 | -26.037 | 26.001 |
|  |  | **0.5** | 57 | 82752.86 | 84139.55 | 0.495 | 0.996 | 1 | -467.462 | -25.853 | 26.167 |
|  |  | **0.3** | 57 | 83032.87 | 83836.33 | 0.496 | 0.996 | 1 | -187.385 | -26.077 | 25.714 |
|  |  | **0** | 57 | 83053.15 | 83854.55 | 0.499 | 0.996 | 1 | -166.978 | -26.279 | 26.224 |
| **0.3** | **1** | **0.7** | 57 | 75108.24 | 84760.22 | 0.495 | n/a | 1 | -8111.98 | -26.156 | 26.575 |
|  |  | **0.5** | 57 | 80064.6 | 85374.3 | 0.496 | n/a | 1 | -3155.62 | -26.119 | 26.244 |
|  |  | **0.3** | 57 | 81251.38 | 84306.95 | 0.497 | n/a | 1 | -1968.86 | -25.728 | 26.119 |
|  |  | **0** | 57 | 82441.31 | 840304.97 | 0.5 | n/a | 1 | -778.796 | -26.192 | 26.136 |
|  | **0.5** | **0.7** | 57 | 79258.46 | 86761.02 | 0.501 | 1 | 1 | -3967.81 | -25.974 | 26.606 |
|  |  | **0.5** | 57 | 81654.5 | 84559.12 | 0.5 | 1 | 1 | -1565.73 | -26.033 | 26.355 |
|  |  | **0.3** | 57 | 82020.38 | 84348.88 | 0.498 | 1 | 1 | -1199.87 | -25.935 | 26.178 |
|  |  | **0** | 57 | 82498.37 | 84334 | 0.3499 | 1 | 1 | -721.773 | -26.325 | 25.98 |
|  | **0.1** | **0.7** | 57 | 81201.09 | 84338.76 | 0.497 | 0.999 | 1 | -2019.22 | -25.937 | 26.208 |
|  |  | **0.5** | 57 | 82143.34 | 84145.19 | 0.499 | 0.999 | 1 | -1076.89 | -26.096 | 26.105 |
|  |  | **0.3** | 57 | 82538.77 | 84266.23 | 0.497 | 0.999 | 1 | -681.45 | -26.203 | 25.858 |
|  |  | **0** | 57 | 82762.92 | 84167.19 | 0.503 | 0.99 | 1 | -457.15 | -26.343 | 26.279 |

*Note.* Model priors are represented in the λ, λ error and residual covariance columns. Model fit was evaluated on the; number of free parameters (*q*), Deviance Information Criterion (*DIC*), Bayesian Information Criterion (*BIC*), posterior predictive *p*-value (*PPP*), prior-posterior predictive *p*-value (*PPPP*), Comparative Fit Index (*CFI*), effective number of parameters (𝑝𝐷), and the lower and upper bound of the χ*^2^* test (LB χ*^2^*, UB χ*^2^*).

*Supplementary Table 5.* Bayesian CFA model fit indices for the unidimensional model.

| λ | λ error | Residual Covariance | *q* | DIC | BIC | PPP | PPPP | CFI | 𝑝𝐷 | LB χ*^2^* | UB χ*^2^* |
| --- | --- | --- | --- | --- | --- | --- | --- | --- | --- | --- | --- |
| **0.7** | **1** | **0.7** | 52 | 83017.24 | 83707.67 | .498 | n/a | 1 | -203.01 | -25.96 | 26.48 |
|  |  | **0.5** | 52 | 83166.6 | 8381.66 | 0.501 | n/a | 1 | -53.611 | -26.18 | 26.03 |
|  |  | **0.3** | 52 | 83197.98 | 83660.93 | 0.503 | n/a | 1 | -22.227 | -25.923 | 25.783 |
|  |  | **0** | 52 | 83261.3 | 83632.83 | 0.499 | n/a | 1 | 41.116 | -25.93 | 25.976 |
|  | **0.5** | **0.7** | 52 | 83115.78 | 83668.09 | 0.499 | 1 | 1 | -104.455 | -26.159 | 26.421 |
|  |  | **0.5** | 52 | 83185.8 | 83666.2 | 0.501 | 1 | 1 | -34.4 | -25.999 | 25.883 |
|  |  | **0.3** | 52 | 83204.04 | 83678.9 | 0.496 | 1 | 1 | -16.086 | -26.086 | 25.954 |
|  |  | **0** | 52 | 83261.47 | 83632.61 | 0.499 | 1 | 1 | 41.283 | -25.941 | 25.967 |
|  | **0.1** | **0.7** | 52 | 83238.15 | 83654.13 | 0.501 | 0.983 | 1 | 11.395 | -26.056 | 25.547 |
|  |  | **0.5** | 52 | 83231.27 | 83675.73 | 0.494 | 0.983 | 1 | 11.0.89 | -26.112 | 26.062 |
|  |  | **0.3** | 52 | 83247.19 | 83645.81 | 0.501 | 0.983 | 1 | 27.112 | -25.919 | 25.64 |
|  |  | **0** | 52 | 83262.98 | 83631.84 | 0.495 | 0.983 | 1 | 42.662 | -25.786 | 26.36 |
| **0.5** | **1** | **0.7** | 52 | 82980.91 | 83735.51 | 0.499 | n/a | 1 | -239.335 | -26.203 | 26.181 |
|  |  | **0.5** | 52 | 83176.72 | 83671.91 | 0.502 | n/a | 1 | -43.453 | -26.092 | 25.892 |
|  |  | **0.3** | 52 | 83182.08 | 83675.35 | 0.502 | n/a | 1 | -38.121 | -25.87 | 25.719 |
|  |  | **0** | 52 | 83261.14 | 83632.96 | 0.4999 | n/a | 1 | 40.959 | -25.932 | 26.019 |
|  | **0.5** | **0.7** | 52 | 82999.79 | 83760.26 | 0.501 | 1 | 1 | -220.448 | -26.123 | 26.088 |
|  |  | **0.5** | 52 | 83184.64 | 83669.62 | 0.501 | 1 | 1 | -35.533 | -26.01 | 25.876 |
|  |  | **0.3** | 52 | 83196.8 | 83660.89 | 0.503 | 1 | 1 | -23.4 | -25.92 | 25.78 |
|  |  | **0** | 52 | 83261.18 | 83632.94 | 0.499 | 1 | 1 | 40.995 | -25.94 | 26.016 |
|  | **0.1** | **0.7** | 52 | 83180.02 | 83656.68 | 0.502 | 0.996 | 1 | -40.206 | -26.161 | 26.122 |
|  |  | **0.5** | 52 | 83199.26 | 83666.35 | 0.502 | 0.996 | 1 | -20.945 | -26.059 | 25.898 |
|  |  | **0.3** | 52 | 83226.81 | 83678.85 | 0.494 | 0.996 | 1 | 6.679 | -25.765 | 25.744 |
|  |  | **0** | 52 | 83261.39 | 83632.6 | 0.498 | 0.996 | 1 | 41.21 | -25.929 | 26.039 |
| **0.3** | **1** | **0.7** | 52 | 83017.33 | 83724.16 | 0.5 | n/a | 1 | -202.912 | -26.272 | 25.955 |
|  |  | **0.5** | 52 | 82934.97 | 83730.7 | 0.501 | n/a | 1 | -285.254 | -26.007 | 26.103 |
|  |  | **0.3** | 52 | 83173.04 | 83679.86 | 0.501 | n/a | 1 | -47.157 | -25.88 | 25.629 |
|  |  | **0** | 52 | 83260.95 | 83633.22 | 0.499 | n/a | 1 | -25.953 | -25.953 | 26.045 |
|  | **0.5** | **0.7** | 52 | 82792.74 | 83832.46 | 0.499 | 1 | 1 | -427.493 | -26.27 | 25.961 |
|  |  | **0.5** | 52 | 83026.96 | 83702.88 | 0.5 | 1 | 1 | -193.259 | -25.94 | 26.145 |
|  |  | **0.3** | 52 | 83157.72 | 83686.15 | 0.501 | 1 | 1 | -62.474 | -25.964 | 25.701 |
|  |  | **0** | 52 | 83260.81 | 83633.41 | 0.499 | 1 | 1 | 40.641 | -25.897 | 26.052 |
|  | **0.1** | **0.7** | 52 | 83051.6 | 83679.41 | 0.501 | 0.999 | 1 | -168.58 | -26.033 | 25.993 |
|  |  | **0.5** | 52 | 83130.29 | 83673.15 | 0.5 | 0.999 | 1 | -89.934 | -26.095 | 25.992 |
|  |  | **0.3** | 52 | 83168.68 | 83668.38 | 0.502 | 0.999 | 1 | -51.511 | -26.03 | 25.893 |
|  |  | **0** | 52 | 83259.19 | 83634.95 | 0.502 | 0.999 | 1 | 39.091 | -25.942 | 26.1 |

*Note.* Model priors are represented in the λ, λ error and residual covariance columns. Model fit was evaluated on the; number of free parameters (*q*), Deviance Information Criterion (*DIC*), Bayesian Information Criterion (*BIC*), posterior predictive *p*-value (*PPP*), prior-posterior predictive p-value (*PPPP*), Comparative Fit Index (*CFI*), effective number of parameters (𝑝𝐷), and the lower and upper bound of the x*^2^* test (LB x*^2^*, UB x*^2^*). n/a = not applicable.

*Supplementary Table 6.* Residual covariances in the unidimensional Bayesian CFA presented in the main text.

|  | **Anxious Depressed** | **Withdrawn Depressed** | **Somatic Complaints** | **Social Problems** | **Thought Problems** | **Attention** | **Rule Breaking** |
| --- | --- | --- | --- | --- | --- | --- | --- |
| **Withdrawn Depressed** | **0.493 (0.316 0.628)**  0.194 (0.095 0.268) |  |  |  |  |  |  |
| **Somatic Complaints** | **0.46 (0.250 0.647)**  0.212 (0.075 0.283) | **0.36 (0.247 0.469)**  0.142 (0.086 0.176) |  |  |  |  |  |
| **Social Problems** | **0.412 (0.122 0.576)**  0.162 (0.032 0.291) | **0.476 (0.271 0.607)**  0.165 (0.073 0.233) | **0.408 (0.224 0.629)**  0.159 (0.080 0.238) |  |  |  |  |
| **Thought Problems** | **0.44 (0.130 0.572)**  0.186 (0.033 0.283) | **0.419 (0.253 0.510)**  0.156 (0.072 0.198) | **0.405 (0.246 0.578)**  0.171 (0.086 0.224) | **0.463 (0.223 0.629)**  0.171 (0.062 0.252) |  |  |  |
| **Attention** | **0.208 (-0.106 0.469)**  0.091 (-0.034 0.265) | **0.339 (0.097 0.579)**  0.134 (0.031 0.222) | **0.355 (0.144 0.590)**  0.154 (0.056 0.257) | **0.388 (0.076 0.560)**  0.151 (0.019 0.296) | **0.481 (0.226 0.658)**  0.201 (0.066 0.320) |  |  |
| **Rule Breaking** | **0.16 (-0.102 0.362)**  0.057 (-0.026 0.154) | **0.309 (0.081 0.461)**  0.1 (0.020 0.155) | **0.304 (0.102 0.527)**  0.113 (0.031 0.186) | **0.375 (0.163 0.499)**  0.12 (0.039 0.186) | **0.359 (0.176 0.514)**  0.123 (0.048 0.192) | **0.338 (0.075 0.504)**  0.122 (0.020 0.223) |  |
| **Aggression** | **0.259 (-0.228 0.632)**  0.101 (-0.062 0.285) | **0.388 (0.038 0.750)**  0.138 0.010 0.246 () | **0.454 (0.103 0.773)**  0.181 (0.033 0.319) | **0.381 (0.071 0.654)**  0.132 (0.017 0.265) | **0.426 (-0.001 0.706)**  0.158 (0.000 0.302) | **0.268 (-0.167 0.595)**  0.103 (-0.042 0.312) | **0.517 (0.185 0.709)**  0.166 (0.038 0.300) |

*Note.* Standardised estimates are presented in bold while 95% credibility intervals with equal tails are shown in brackets.

1. ***Factor Mixture Models***

*Supplementary Table 7.* Latent Class Analysis on CBCL summed scale scores.

| **Classes** | *q* | LL | BIC | LMR | LMR *p* | Entropy (*E*) | Smallest Class  *n* (*%*) |
| --- | --- | --- | --- | --- | --- | --- | --- |
| **1** | 16 | -104809.65 | 209769.4 |  |  |  |  |
| **2** | 25 | -88223.68 | 176681.88 | 32783.64 | >.001 | 0.902 | 3920, 33.05% |
| **3** | 34 | -84342.82 | 169004.58 | 7670.87 | >.001 | 0.859 | 1,706, 14.38% |
| **4** | 43 | -83014.84 | 166433.061 | 2624.86 | >.001 | 0.853 | 1565, 13.2% |
| **5** | 52 | -82020.82 | 164529.45 | 1964.769 | 0.016 | 0.852 | 853, 7.19% |
| **6** | 61 | -79472.93 | 159518.09 | 3408.127 | >.001 | 0.932 | 916, 7.72% |
| **7** | 70 | -75332.31 | 151321.29 | 2781.398 | 0.008 | 0.954 | 630, 5.31% |
| **8** | 79 | -72920.81 | 146582.71 | 1956.789 | 0.031 | 0.959 | 281, 2.37% |
| **9** | 88 | -69492.17 | 139809.9 | -2708.454 | 0.5611 | 0.956 | 379, 3.2% |
| **10** | 97 | -65214.55 | 131339 | -2866.906 | 0.4229 | 0.958 | 238, 2.01% |

*Note.* Fit statistics were evaluated on; the number of free parameters (*q*), log likelihood (*LL*), Lo-Mendell-Rubin adjusted LRT test (LMR), entropy and the size of the smallest class.

| # C | Model | *Q* | LL | BIC | AIC | LMR | LMR *p* | *E* | Smallest Class  (*n, %*) |
| --- | --- | --- | --- | --- | --- | --- | --- | --- | --- |
| 1 |  | 28 | -83660.94 | 167584.5 | 167377.9 |  |  |  |  |
| 2 | FMM1 | 29 | -88223.68 | 176719.4 | 176595.4 | 32310.87 | 0.24 | 0.902 | 3120, 26.30% |
|  | ZI-FMM1 | 26 | -90713 | 181669.9 | 181478 | 25477.43 | 0.001 | 0.908 | 3524, 29.71% |
|  | FMM2 | 35 | -82850.56 | 166029.4 | 165771.1 | 13662.33 | 0.001 | 0.765 | 2573, 21.69% |
|  | ZI-FMM2 | 29 | -83660.94 | 167593.9 | 167379.9 | 0 | 0.5 | 1 | 0, 0% |
|  | FMM3 | 41 | -81977.67 | 164340 | 164037.3 | 3336.898 | 0.24 | 0.829 | 5671, 47.81% |
|  | ZI-FMM3 | 38 | -83283.76 | 166924 | 166643.5 | 745.525 | 0.001 | 0.943 | 615, 5.18% |
|  | FMM4 | 53 | -82580.5 | 165658.2 | 165267 | 2150.915 | 0.208 | 0.876 | 1975, 16.65% |
|  | ZI-FMM4 | 39 | -83283.76 | 166933.4 | 166645.5 | 745.525 | 0.001 | 0.943 | 614, 5.18% |
| 3 | FMM1 | 33 | -84445.66 | 169200.9 | 168957.3 | 7359.895 | 0.24 | 0.859 | 1682, 14.18% |
|  | ZI-FMM1 | 30 | -84888.97 | 170059.4 | 169837.9 | 6026.95 | 0.178 | 0.874 | 1358, 11.45% |
|  | FMM2 |  |  |  |  |  |  |  |  |
|  | ZI-FMM2 | 36 | -83453.99 | 167245.7 | 166980 | 405.274 | 0.001 | 0.962 | 0, 0% |
|  | FMM3 |  |  |  |  |  |  |  |  |
|  | ZI-FMM3 | 50 | -81344.12 | 163157.3 | 162788.2 |  |  | 0.865 | 1083, 9.13% |
|  | FMM4 | 76 | -82580.5 | 165874 | 165313 |  |  | 0.922 | 0, 0% |
|  | ZI-FMM4 | 62 | -82066.12 | 164713.8 | 164256.2 |  |  | 0.82 | 2979, 25.11% |
| 4 | FMM1 | 41 | -83317.33 | 166981.8 | 166708.7 | 2198.081 | 0.24 | 0.856 | 1527, 12.87% |
|  | ZI-FMM1 | 34 | -83981.9 | 168282.8 | 168031.8 | 838.174 | 0.24 | 0.841 | 412, 3.47% |
|  | FMM2 | 49 | -83660.94 | 167781.5 | 167419.9 |  |  | 1 | 0, 0% |
|  | ZI-FMM2 | 43 | -83453.99 | 167311.3 | 166994 | 410.27 | 0.001 | 0.97 | 0, 0% |
|  | FMM3 | 65 | -74008.71 | 148627.2 | 148147.4 |  |  | 1 | 0, 0% |
|  | ZI-FMM3 | 65 | -7785.56 | 156232.7 | 155775.1 | 355.165 | 0.027 | 0.98 | 208, 1.75% |
|  | FMM4 | 99 | -82580.5 | 166089.7 | 165359 |  |  | 0.938 | 0, 0% |
|  | ZI-FMM4 | 77 | -82580.5 | 165883.3 | 165315 |  |  | 0.938 | 0, 0% |
| 5 | FMM1 | 41 | -81916.59 | 164217.8 | 163915.2 | 2728.77 | 0.24 | 0.925 | 1563, 13.18% |
|  | ZI-FMM1 | 38 | -83668.93 | 167694.3 | 167413.9 | 1521.03 | 0.24 | 0.861 | 0, 0% |
|  | FMM2 | 56 | -82850.67 | 166226.7 | 165813.3 | 1613.665 | 0.001 | 0.899 | 0, 0% |
|  | ZI-FMM2 | 50 | -83453.99 | 167377 | 167008 | 411.335 | 0.001 | 0.974 | 0, 0% |
|  | FMM3 | 77 | -74008.71 | 148739.8 | 148171.4 | 0 | 0.5 | 1 | 0, 0% |
|  | ZI-FMM3 | 74 | -78005.25 | 156704.7 | 156158.5 | 0 | 0.24 | 0.996 | 0, 0% |
|  | FMM4 | 122 | -83656.59 | 168457.7 | 167557.2 |  |  | 1 | 0, 0% |
|  | ZI-FMM4 | 92 | -82580.5 | 166024 | 165345 |  |  | 0.946 | 0, 0% |
| 6 | FMM1 |  |  |  |  |  |  |  |  |
|  | ZI-FMM1 | 42 | -83570.22 | 167534.4 | 167224.4 | -209.917 | 1 | 0.858 | 0, 0% |
|  | FMM2 |  |  |  |  |  |  |  |  |
|  | ZI-FMM2 | 57 | -83453.99 | 167442.7 | 167002 | 412.224 | 0.24 | 0.976 | 0, 0% |
|  | FMM3 | 89 | -69912.66 | 140660.2 | 140003.3 | 0 | 0.51 | 1 | 0, 0% |
|  | ZI-FMM3 |  |  |  |  |  |  |  |  |
|  | FMM4 |  |  |  |  |  |  |  |  |
|  | ZI-FMM4 |  |  |  |  |  |  |  |  |
| 7 | FMM1 | 49 | -78989.24 | 158438.1 | 158076.5 | 1181.821 | 0.24 | 0.899 | 590, 4.97% |
|  | ZI-FMM1 | 46 | -81394.43 | 163220.4 | 162880.9 | 508.001 | 0.24 | 0.821 | 311, 2.62% |
|  | FMM2 |  |  |  |  |  |  |  |  |

*Supplementary Table 8.* Model fit indices for the Bi-factor mixture models

*Supplementary Table 8 continued.*

*Note. Q =* Number of free parameters, *LL* = Log-likelihood, *BIC* = Bayesian Information Criterion, *AIC =*Akaike Information Criterion, *LMR* = Lo-Mendell-Rubin test. *E* = Entropy. Each model was run with 20,000 starting values. The number of classes is indicated in the #C column, while the type of mixture model is shown in the Model column (ZI denotes zero-inflated models). Models highlighted in blue indicate warnings from Mplus regarding the trustworthiness of model parameter estimates standard errors, while those highlighted in red indicate models that failed to converge. The chosen solutions are highlighted in green.

| # C | Model | *Q* | LL | BIC | AIC | LMR | LMR *p* | *E* | Smallest Class  (*n, %*) |
| --- | --- | --- | --- | --- | --- | --- | --- | --- | --- |
| 7 | ZI-FMM2 | 64 | -83453.99 | 167508.3 | 167036 | 412.582 | 0.001 | 0.978 | 0, 0% |
|  | FMM3 |  |  |  |  |  |  |  |  |
|  | ZI-FMM3 | 98 | -74009.34 | 148938 | 148214.7 | 0 | 0.24 | 1 | 0, 0% |
|  | FMM4 |  |  |  |  |  |  |  |  |
|  | ZI-FMM4 |  |  |  |  |  |  |  |  |

| *# C* | *Model* | *Q* | *LL* | *BIC* | *AIC* | *LMR* | *LMR p* | *Entropy* | *Smallest Class*  *(n,%)* |
| --- | --- | --- | --- | --- | --- | --- | --- | --- | --- |
| 1 |  | 24 | -84671.91 | 169569 | 169391.8 |  |  |  |  |
| 2 | FMM1 | 26 | -83909.72 | 168063.3 | 167871.4 | 1447.239 | 0.001 | 0.742 | 3108, 26.20% |
|  | ZI-FMM1 | 24 | -88223.68 | 176672.5 | 176495.4 | 29976.47 | 0.001 | 0.902 | 3920, 33.05% |
|  | FMM2 | 27 | -83579.59 | 167412.5 | 167213.2 | 2109.674 | 0.001 | 0.548 | 4743, 39.98% |
|  | ZI-FMM2 | 25 | -83782.31 | 167799.1 | 167614.6 | 1607.8 | 0.001 | 0.638 | 4636, 39.08% |
|  | FMM3 | 34 | -82127.67 | 164574.3 | 164323.3 | 5034.8 | 0.001 | 0.823 | 3825, 32.25% |
|  | ZI-FMM3 | 33 | -83393.34 | 167096.3 | 166852.7 | 2527.196 | 0.001 | 0.647 | 4389, 37.00% |
|  | FMM4 | 43 | -81798.91 | 164001.2 | 163683.8 | 5712.159 | 0.001 | 0.817 | 4352, 36.69% |
|  | ZI-FMM4 | 35 | -83393.34 | 167115 | 166856.7 | 2530.157 | 0.001 | 0.647 | 4389, 37.00% |
| 3 | FMM1 | 28 | -83612.59 | 167487.8 | 167281.2 | 564.195 | 0.001 | 0.763 | 1295, 10.92% |
|  | ZI-FMM1 | 26 | -84735.87 | 169715.6 | 169523.7 | 6303.646 | 0.001 | 0.861 | 1576, 13.29% |
|  | FMM2 | 30 | -83196.59 | 166674.6 | 166453.2 | 739.721 | 0.001 | 0.616 | 1639, 13.82% |
|  | ZI-FMM2 | 28 | -83367.36 | 166997.4 | 166790.7 | 383.57 | 0.001 | 0.548 | 2799, 23.60% |
|  | FMM3 | 44 | -78777.49 | 157967.7 | 157643 | 6629.689 | 0.001 | 0.994 | 1662, 14.01% |
|  | ZI-FMM3 |  |  |  |  |  |  |  |  |
|  | FMM4 | 61 | -80564.59 | 161701.4 | 161251.2 | 2454.11 | 0.001 | 0.812 | 3105, 26.18% |
|  | ZI-FMM4 |  |  |  |  |  |  |  |  |
| 4 | FMM1 | 30 | -83470.87 | 167223.2 | 167001.7 | 269.09 | 0.001 | 0.761 | 783, 6.60% |
|  | ZI-FMM1 | 28 | -83573.63 | 167409.9 | 167203.3 | 1690.91 | 0.001 | 0.8 | 983, 8.29% |
|  | FMM2 | 33 | 83192.26 | 166694.1 | 166450.5 | 8.353 | 0.052 | 0.696 | 7, 0.06% |
|  | ZI-FMM2 | 31 | -83196.59 | 166684 | 166455.2 | 0 | 0.5 | 0.695 | 0, 0% |
|  | FMM3 | 54 | -74776.21 | 150059 | 149660.4 | 7918.164 | 0.001 | 0.999 | 1008, 8.50% |
|  | ZI-FMM3 | 53 | -77445.32 | 155387.8 | 154996.6 | 2633.161 | 0.001 | 0.991 | 333, 2.81% |
|  | FMM4 | 79 | -78932.95 | 158607 | 158023.9 |  |  | 0.976 | 680, 5.73% |
|  | ZI-FMM4 | 71 | -78604.37 | 157874.8 | 157350.7 | 4567.741 | 0.001 | 0.985 | 641, 5.40% |
| 5 | FMM1 | 32 | -83362.4 | 167025 | 166788.8 | 205.968 | 0.001 | 0.784 | 374, 3.15% |
|  | ZI-FMM1 | 30 | -83363.38 | 167008.2 | 166786.8 | 350.558 | 0.001 | 0.782 | 344, 2.90% |
|  | FMM2 | 36 | -83192.26 | 166722.5 | 166456.5 | 8.499 | 0.049 | 0.738 | 0, 0% |
|  | ZI-FMM2 | 34 | -83196.59 | 166712.1 | 166461.2 | 0 | 0.5 | 0.738 | 0, 0% |
|  | FMM3 | 64 | -7032.41 | 142065.2 | 141592.8 | 8002.289 | 0.001 | 1 | 632, 5.33% |
|  | ZI-FMM3 | 63 | -71991.74 | 144574.5 | 144109.5 | 5503.742 | 0.001 | 1 | 379, 3.20% |
|  | FMM4 | 97 | -78169.81 | 157249.6 | 156533.6 | 1517.293 | 0.24 | 0.913 | 0, 0% |
|  | ZI-FMM4 | 89 | -75190.42 | 151215.7 | 150558.8 |  |  | 1 | 630, 5.31% |
| 6 | FMM1 | 34 | -83285.67 | 166890.3 | 16639.3 | 154.693 | 0.001 | 0.789 | 238, 2.01% |
|  | ZI-FMM1 | 32 | -83362.87 | 167025.9 | 166789.7 | 0 | 0.5 | 0.803 | 0, 0% |
|  | FMM2 | 39 | -83192.26 | 166750.4 | 166462.5 | 8.549 | 0.049 | 0.765 | 0, 0% |
|  | ZI-FMM2 | 37 | -83158.33 | 166663.8 | 166390.7 | 74.52 | 0.32 | 0.76 | 0, 0% |
|  | FMM3 | 74 | -66405.24 | 133504.7 | 132958.5 | 8563.068 | 0.006 | 1 | 379, 3.20% |
|  | ZI-FMM3 | 73 | -69787.68 | 140260.2 | 139721.4 | 1876.348 | 0.001 | 0.911 | 632, 5.33% |
|  | FMM4 | 115 | -78225.2 | 157529.2 | 156680.4 | 0 | 0.24 | 0.907 | 0, 0% |
|  | ZI-FMM4 | 107 | -78592.98 | 158189.7 | 157400 | 0 | 0.24 | 0.988 | 0, 0% |
| 7 | FMM1 | 36 | -83246.31 | 166830.3 | 166564.6 | 74.726 | 0.359 | 0.777 | 210, 1.77% |
|  | ZI-FMM1 | 34 | -83328.73 | 166976.4 | 166725.5 | 3.004 | 0.029 | 0.726 | 117, 0.99% |
|  | FMM2 | 42 | -83192.6 | 16678.5 | 166468.5 | 0 | 0.5 | 0.784 | 0, 0% |

*Supplementary Table 9.* Model fit indices for the Unidimensional mixture models

*Supplementary Table 9 continued.*

*Note. Q =* Number of free parameters, *LL* = Log-likelihood, *BIC* = Bayesian Information Criterion, *AIC =*Akaike Information Criterion, *LMR* = Lo-Mendell-Rubin test. Each model was run with 20,000 starting values. The number of classes is indicated in the #C column, while the type of mixture model is shown in the Model column (ZI denotes zero-inflated models). Models highlighted in blue indicate warnings from Mplus regarding the trustworthiness of model parameter estimates standard errors, while models highlighted in red indicate non-convergent models. The chosen solution is highlighted in green

| *# C* | *Model* | *Q* | *LL* | *BIC* | *AIC* | *LMR* | *LMR p* | *Entropy* | *Smallest Class*  *(n,%)* |
| --- | --- | --- | --- | --- | --- | --- | --- | --- | --- |
|  | ZI-FMM2 | 40 | -83158.3 | 166691.9 | 166396.7 | 75.5 | 0.316 | 0.779 | 0, 0% |
|  | FMM3 | 84 | -66267.65 | 133323.3 | 132703.3 | 272.275 | 0.001 | 0.98 | 379, 3.2% |
|  | ZI-FMM3 | 83 | -67356.35 | 135491.3 | 134878.7 | -1879.972 | 1 | 0.98 | 379, 3.2% |
|  | FMM4 | 133 | -77831.96 | 156911.6 | 155929.9 | 0 | 0.24 | 0.982 | 0, 0% |
|  | ZI-FMM4 |  |  |  |  |  |  |  |  |

1. ***Item Pruning with Unidimensional Item Response Theory Graded Response Models***

*Supplementary Table 10.* Latent Class analysis on each CBCL subscale

| Subscale | Classes | *Q* | *LL* | BIC | LMR | LMR *p* | Entropy | Smallest class  (*n,%*) |
| --- | --- | --- | --- | --- | --- | --- | --- | --- |
| Anxious Depressed | 1 | 22 | -63214.8 | 126636 |  |  |  |  |
|  | 2 | 45 | -56180.82 | 112783.8 | 14003.07 | <.001 | 0.824 | 3007, 25.35% |
|  | 3 | 68 | -55103.63 | 110845.2 | 2144.442 | <.001 | 0.778 | 719, 6.06% |
|  | 4 | 91 | -54764.57 | 110382.8 | 674.983 | <.001 | 0.753 | 726, 6.12% |
|  | 5 | 114 | -54529.08 | 110127.6 | 468.812 | <.001 | 0.744 | 359, 3.03% |
|  | **6** | **137** | **-54394.98** | **110075.2** | **266.959** | **<.001** | **0.752** | **328, 2.77%** |
|  | 7 | 160 | -54298.22 | 110097.4 | 192.628 | 0.0126 | 0.754 | 236, 1.99% |
|  | 8 | 183 | -54227.69 | 110172.1 | 140.398 | 0.219 | 0.767 | 158, 1.33% |
|  | 9 | 206 | -54168.16 | 110268.8 | 118.525 | 0.455 | 0.755 | 152, 1.28% |
|  | 10 | 229 | -54119.66 | 110387.6 | 96.548 | 0.556 | 0.726 | 104, 0.88% |
| Withdrawn Depressed | 1 | 16 | -36873.82 | 73897.73 |  |  |  |  |
|  | 2 | 33 | -32796.69 | 65902.96 | 8103.442 | <.001 | 0.829 | 2072, 17.47% |
|  | 3 | 50 | -32442.81 | 65354.67 | 703.357 | <.001 | 0.748 | 531, 4.48% |
|  | 4 | 67 | -32347.92 | 65324.37 | 188.591 | 0.0419 | 0.721 | 281, 2.37% |
|  | **5** | **84** | **-32267.95** | **65323.92** | **158.941** | **0.0314** | **0.781** | **342, 2.88%** |
|  | 6 | 101 | -32213.16 | 65373.81 | 108.897 | 0.0827 | 0.753 | 198, 1.67% |
|  | 7 | 118 | -32167.5 | 65441.98 | 90.747 | 0.3873 | 0.8 | 210, 1.77% |
|  | 8 | 135 | -32133.57 | 65533.58 | 67.449 | 0.299 | 0.81 | 29, 0.24% |
|  | 9 | 152 | -32103.81 | 65633.55 | 59.136 | 0.5653 | 0.8 | 21, 0.18% |
|  | 10 | 169 | -32084.6 | 65754.6 | 38.189 | 0.7365 | 0.805 | 22, 0.19% |
|  | 2 | 45 | -50455.22 | 101332.6 | 10808.15 | <.001 | 0.817 | 2593, 21.86% |
|  | 3 | 68 | -49761.68 | 100161.3 | 1380.69 | <.001 | 0.731 | 831, 7.01% |

*Supplementary Table 10 continued.*

| Subscale | Classes | *Q* | *LL* | BIC | LMR | LMR *p* | Entropy | Smallest class  (*n,%*) |
| --- | --- | --- | --- | --- | --- | --- | --- | --- |
| Social Problems | 4 | 91 | -49380.44 | 99614.57 | 758.947 | <.001 | 0.769 | 697, 5.88% |
|  | **5** | **114** | **-49196.21** | **99461.86** | **366.773** | **0.004** | **0.785** | **443, 3.73%** |
|  | 6 | 137 | -49102.9 | 99491.01 | 185.751 | 0.425 | 0.789 | 242, 2.04% |
|  | 7 | 160 | -49024.73 | 99550.44 | 155.615 | 0.2745 | 0.774 | 201, 1.69% |
|  | 8 | 183 | -48957.14 | 99631.02 | 134.567 | 0.2177 | 0.777 | 177, 1.49% |
|  | 9 | 206 | -48904.96 | 99742.43 | 103.867 | 0.349 | 0.779 | 86, 0.73% |
|  | 10 | 229 | -48861.62 | 99871.51 | 86.294 | 0.459 | 0.774 | 86, 0.73% |
| Somatic Complaints | 1 | 22 | -50643.23 | 101492.8 |  |  |  |  |
|  | 2 | 45 | -46252.23 | 92926.61 | 8741.478 | <.001 | 0.797 | 2783, 23.46% |
|  | 3 | 68 | -45836.96 | 92311.83 | 826.708 | <.001 | 0.787 | 480, 4.05% |
|  | 4 | 91 | -45650.26 | 92154.2 | 371.674 | <.001 | 0.702 | 474, 4.00% |
|  | **5** | **114** | **-45489.2** | **92047.84** | **320.631** | **<.001** | **0.727** | **222, 1.87%** |
|  | 6 | 137 | -45426.86 | 92138.92 | 124.113 | 0.0143 | 0.721 | 215, 1.81% |
|  | 7 | 160 | -45383.86 | 92268.67 | 85.609 | 0.3638 | 0.731 | 163, 1.37% |
|  | 8 | 183 | -45347.03 | 92410.97 | 73.306 | 0.7603 | 0.744 | 40, 0.34% |
|  | 9 | 206 | -45310.13 | 92552.75 | 73.466 | 0.8066 | 0.737 | 45, 0.38% |
|  | 10 | 229 | -45281.76 | 92711.77 | 67.787 | 0.79 | 0.71 | 45, 0.38% |
| Thought Problems | 1 | 22 | -37681.3 | 75568.98 |  |  |  |  |
|  | 2 | 45 | -35256.89 | 70935.92 | 4826.455 | <.001 | 0.756 | 2152, 18.14% |
|  | **3** | **68** | **-35032.77** | **70703.45** | **446.167** | **<.001** | **0.656** | **583, 4.91%** |
|  | 4 | 91 | -34925.18 | 70704.05 | 214.172 | <.001 | 0.724 | 116, 0.98% |
|  | 5 | 114 | -34845.24 | 70759.92 | 159.156 | 1 | 0.749 | 55, 0.46% |
|  | 6 | 137 | -34792.98 | 70871.17 | 104.294 | 0.1663 | 0.699 | 72, 0.61% |
|  | 7 | 160 | -34750.57 | 71002.12 | 84.423 | 1 | 0.727 | 59, 0.50% |
|  | 8 |  |  |  |  |  |  |  |
|  | 9 |  |  |  |  |  |  |  |
|  | 10 |  |  |  |  |  |  |  |
| Attention Problems | 1 | 26 | -100039.4 | 200322.7 |  |  |  |  |
|  | 2 | 53 | -85490.17 | 171477.5 | 28923.97 | <.001 | 0.885 | 4048, 34.13% |
|  | 3 | 80 | -82152.87 | 165056.2 | 6648.352 | <.001 | 0.877 | 1304, 10.99% |
|  | 4 | 107 | -81520.56 | 164044.9 | 1259.653 | <.001 | 0.77 | 1039, 8.76% |
|  | 5 | 134 | -81084.43 | 163425.9 | 868.824 | <.001 | 0.75 | 1036, 8.73% |
|  | **6** | **161** | **-80818.37** | **163147.1** | **530.034** | **<.001** | **0.754** | **576, 4.86%** |
|  | 7 | 188 | -80579.85 | 162923.3 | 475.168 | 0.0748 | 0.765 | 310, 2.61% |
|  | 8 | 215 | -80431.92 | 162880.8 | 294.696 | 0.1084 | 0.763 | 227, 1.91% |
|  | 9 | 242 | -80317.81 | 162905.9 | 227.311 | 0.0098 | 0.748 | 463, 3.90% |
|  | 10 | 269 | -80221.16 | 162966 | 192.3540 | 0.6795 | 0.756 | 195, 1.64% |

*Supplementary Table 10 continued.*

| Subscale | Classes | *Q* | *LL* | BIC | LMR | LMR *p* | Entropy | Smallest class  (*n,%*) |
| --- | --- | --- | --- | --- | --- | --- | --- | --- |
| Rule Breaking | 1 | 35 | -41930.52 | 84189.38 |  |  |  |  |
|  | 2 | 71 | -36917.63 | 74501.31 | 9996.186 | <.001 | 0.846 | 2296, 19.36% |
|  | **3** | **107** | **-36120.4** | **73244.59** | **1589.741** | **<.001** | **0.822** | **445, 3.75%** |
|  | 4 | 143 | -35995.78 | 73333.07 | 248.504 | 0.0139 | 0.828 | 319, 2.69% |
|  | 5 | 179 | -35894.89 | 73468.99 | 201.195 | 0.7747 | 0.822 | 114, 0.96% |
|  | 6 | 215 | -35831.38 | 73679.7 | 126.642 | 0.3627 | 0.826 | 144, 1.21% |
|  | 7 | 251 | -35782.61 | 73919.87 | 97.248 | 0.7664 | 0.759 | 181, 1.53% |
|  | 8 |  |  |  |  |  |  |  |
|  | 9 |  |  |  |  |  |  |  |
|  | 10 | 359 | -35660.05 | 74687.91 | 78.341 | 0.7611 | 0.772 | 38, 0.32% |
| Aggressive Behaviours | 1 | 36 | -96629.79 | 193597.3 |  |  |  |  |
|  | 2 | 73 | -80620.84 | 161926.5 | 31925.94 | <.001 | 0.894 | 3107, 26.19% |
|  | 3 | 110 | -76865.82 | 154763.6 | 7488.461 | <.001 | 0.864 | 990, 8.35% |
|  | 4 | 147 | -75948.46 | 153275.9 | 1829.449 | <.001 | 0.827 | 383, 3.23% |
|  | **5** | **184** | **-75506.4** | **152738.9** | **881.574** | **<.001** | **0.823** | **390, 3.29%** |
|  | 6 | 221 | -75181 | 152435.2 | 648.934 | 0.0049 | 0.795 | 286, 2.41% |
|  | 7 | 258 | -74926.91 | 152274.1 | 506.716 | 0.0585 | 0.815 | 256, 2.16% |
|  | 8 | 295 | -74669.47 | 152106.4 | 513.401 | 0.0031 | 0.78 | 235, 1.98% |
|  | 9 | 332 | -74501.22 | 152117 | 335.54 | 0.7753 | 0.785 | 206, 1.74% |
|  | 10 |  |  |  |  |  |  |  |

*Note. Q =* Number of free parameters, *LL* = Log-likelihood, BIC = Bayesian Information Criterion, LMR = Lo-Mendell-Rubin test. Best fitting class solutions for each subscale are highlighted in bold, while those highlighted red indicate models that did not terminate normally.

*
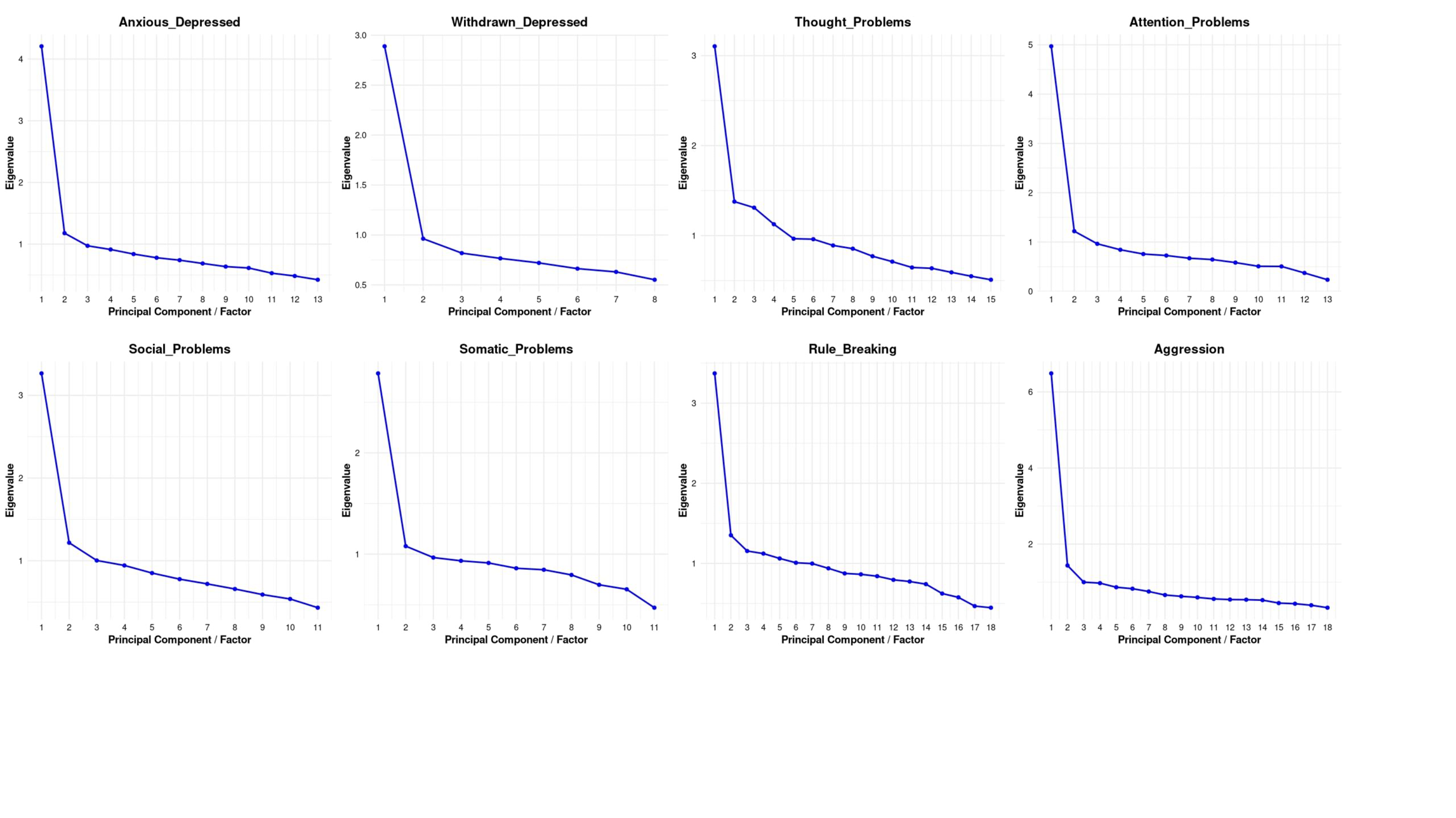

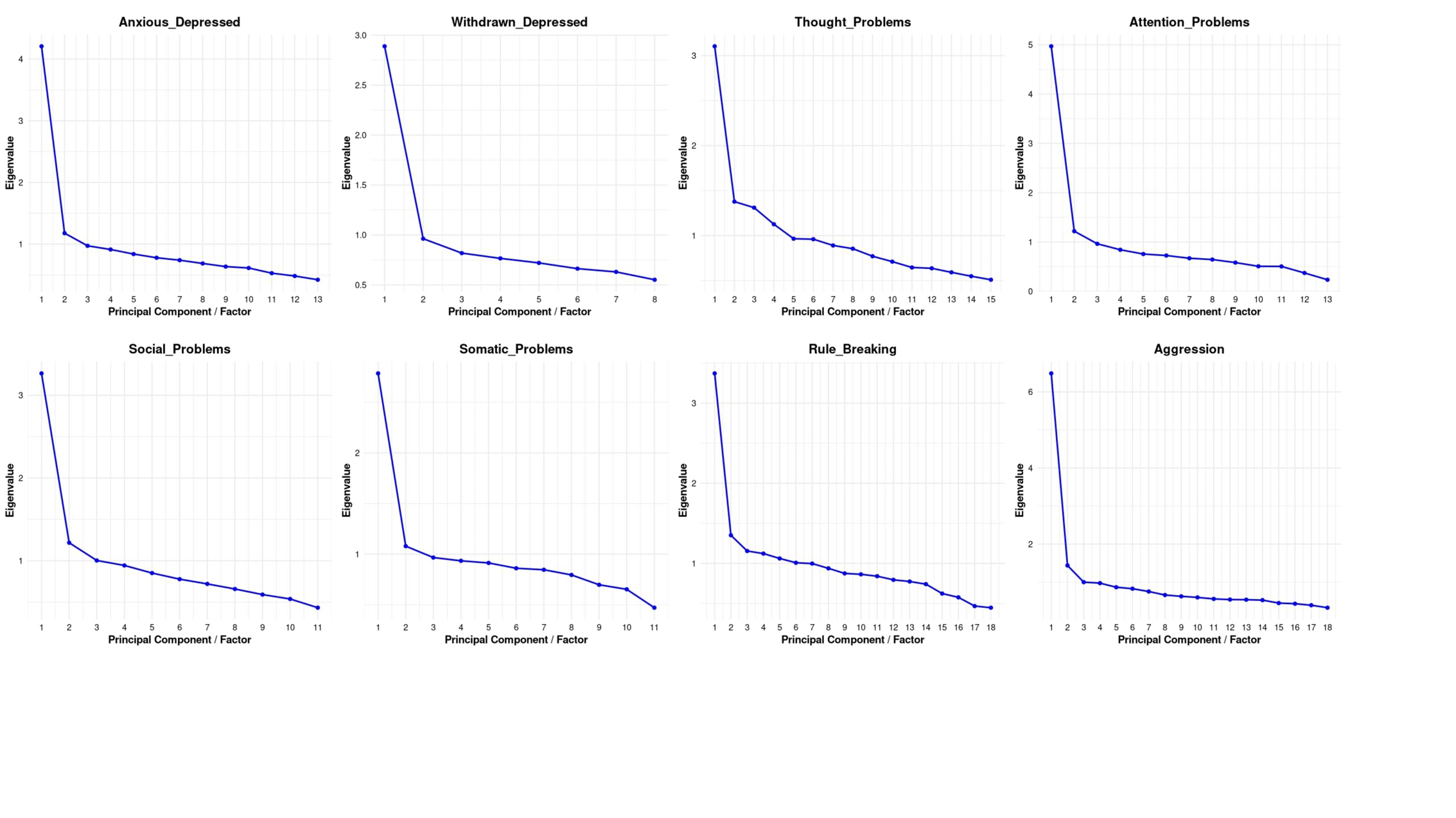
*

*Supplementary Figure 3.* Scree plots for each subscale. Across subscales the first eigenvalue explains substantially more variance than subsequent values.

*
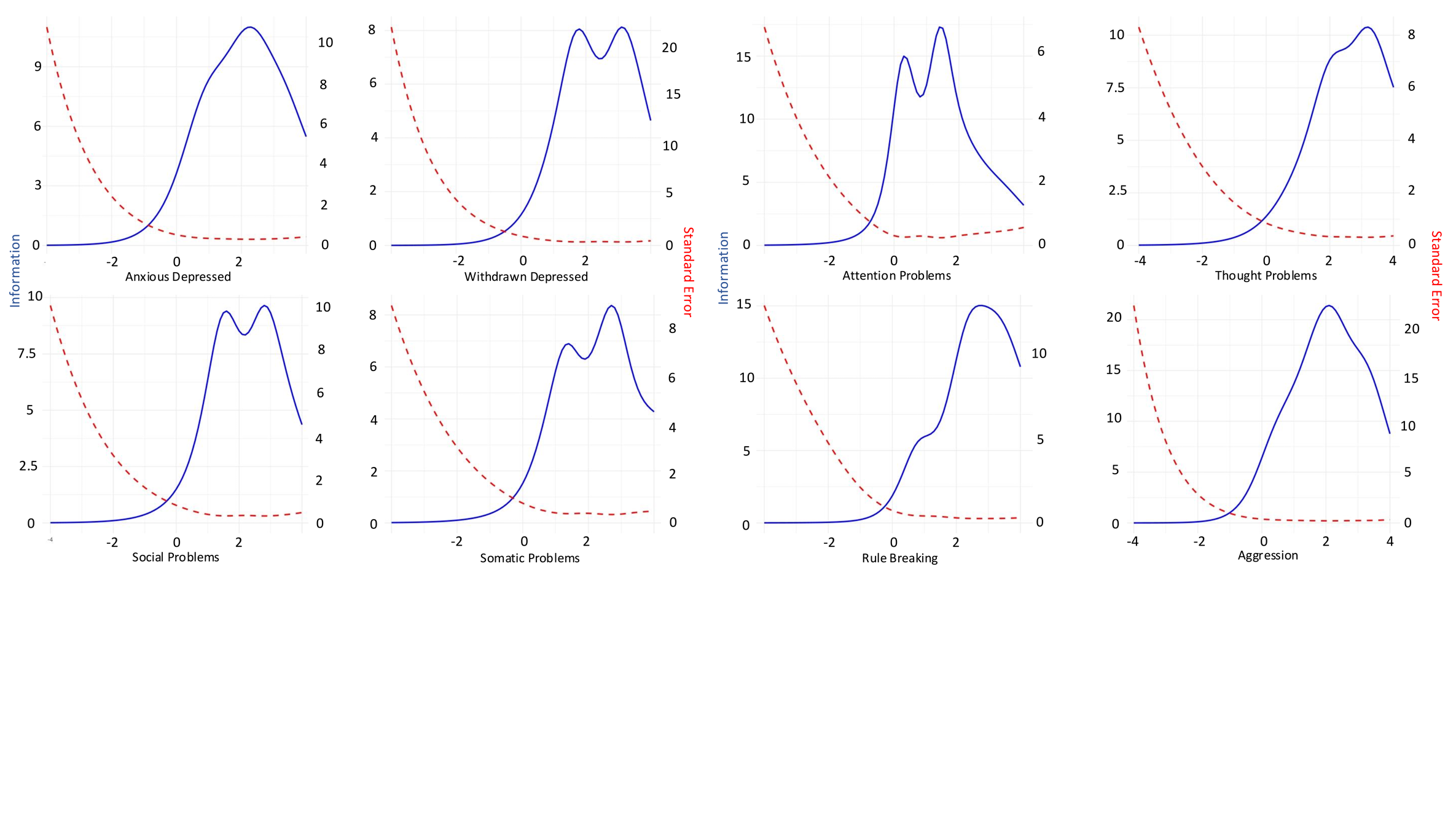

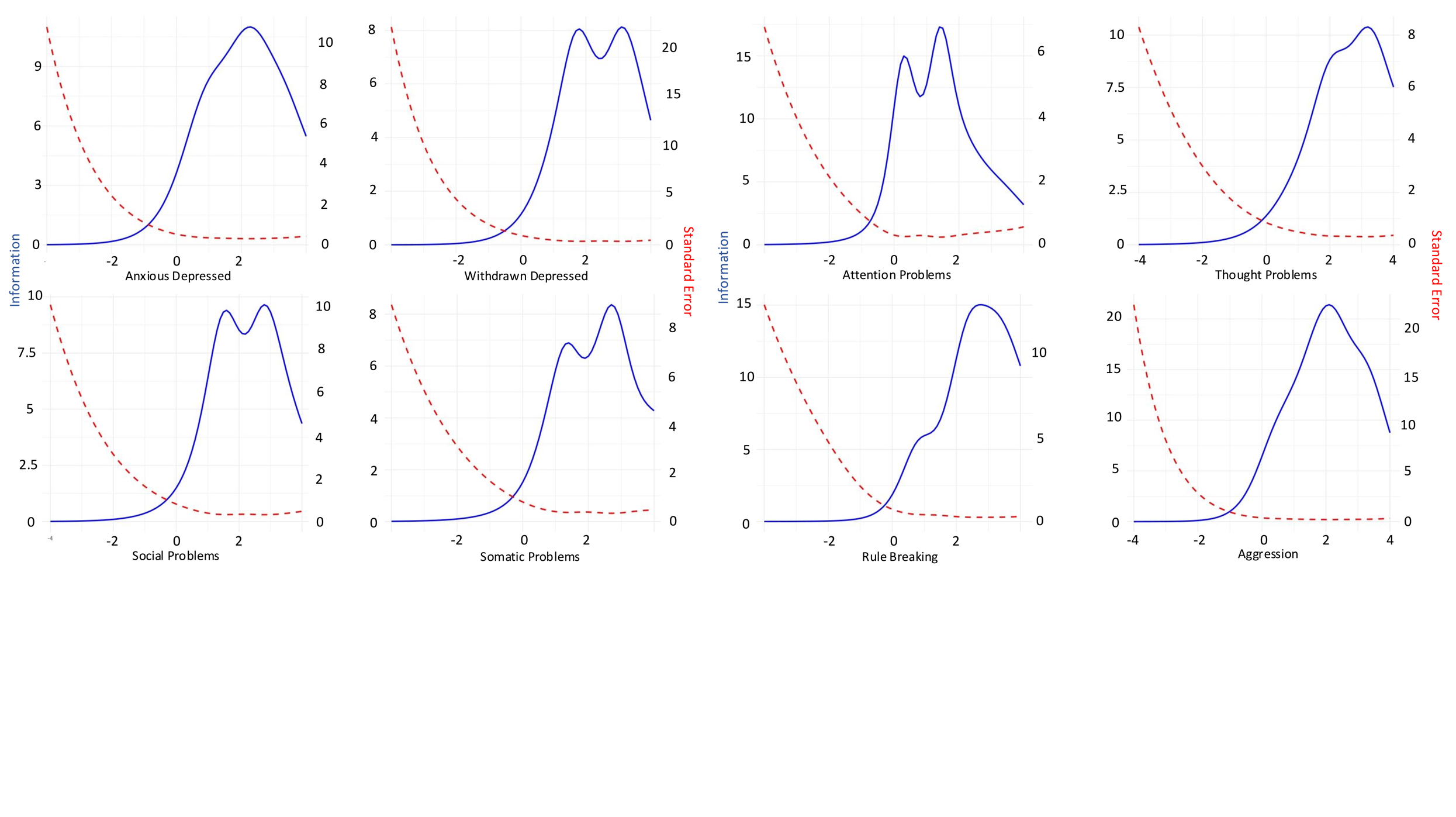
*

*Supplementary Figure 4.* Total Information Function / Curve for each subscale after graded response models were fit on all items in the entire sample. Blue solid lines indicate information (plotted on the left *y*-axis), and thus reliability of the subscale along the latent trait continuum (*r_xx_* = 1- $\frac{1}{I}$) while red dashed lines indicate standard error (*SE* = $\frac{1}{\sqrt{I}}$) (plotted on the right *y*-axis).

*Supplementary Table 11.* Reliability of each subscale (*r_xx_ =* 1 – 1/*I*) along the latent trait continuum after inspection of the total information function (TIF) for all items and all participants on each subscale.

| **Scale** | **Reliability (*r_xx_*) across the latent Trait Continuum (**θ**)** | | | | | | | | | | | | |
| --- | --- | --- | --- | --- | --- | --- | --- | --- | --- | --- | --- | --- | --- |
|  | **-3.0 *SD*** | **-2.5 *SD*** | **-2.0 *SD*** | **-1.5 *SD*** | **-1.0**  ***SD*** | **-0.5 *SD*** | ***M*** | **0.5 SD** | **1.0 *SD*** | **1.5 *SD*** | **2.0 *SD*** | **2.5 *SD*** | **3.0 *SD*** |
| **Anxious Depressed** | 0.036  (1.04) | 0.073  (1.08) | 0.146  (1.17) | 0.273  (1.38) | 0.453 (1.83) | 0.642  (2.79) | 0.784 (4.64) | 0.862 (7.25) | 0.893 (9.37) | 0.905 (10.53) | 0.915 (11.73) | 0.915  (11.78) | 0.904 (10.42) |
| **Withdrawn Depressed** | 0.01 (1.01) | 0.021 (1.02) | 0.046 (1.05) | 0.096 (1.11) | 0.192 (1.24) | 0.345 (1.53) | 0.533 (2.14) | 0.703 (3.36) | 0.819 (5.54) | 0.880 (8.36) | 0.886 (8.75) | 0.874 (7.95) | 0.889 (9.02) |
| **Social Problems** | 0.03 (1.03) | 0.052 (1.06) | 0.093 (1.1) | 0.162 (1.19) | 0.271 (1.37) | 0.424 (1.73) | 0.6 (2.5) | 0.759 (4.14) | 0.864 (7.34) | 0.903 (10.32) | 0.895 (9.52) | 0.9 (10) | 0.903 (10.33) |
| **Somatic Problems** | 0.034 (1.03) | 0.055 (1.06) | 0.093 (1.1) | 0.158 (1.19) | 0.263 (1.36) | 0.417 (1.72) | 0.604 (2.52) | 0.764 (4.24) | 0.854 (6.85) | 0.872 (7.8) | 0.864 (7.38) | 0.889 (8.99) | 0.883 (8.56) |
| **Attention Problems** | 0.06 (1.06) | 0.104 (1.12) | 0.182 (1.22) | 0.316 (1.46) | 0.522 (2.09) | 0.766 (4.27) | 0.916 (11.9) | 0.933 (14.98) | 0.927 (13.7) | 0.945 (18.2) | 0.917 (12.03) | 0.885 (8.68) | 0.856 (6.93) |
| **Thought Problems** | 0.036 (1.04) | 0.059 (1.06) | 0.1 (1.11) | 0.168 (1.2) | 0.275 (1.38) | 0.421 (1.73) | 0.58 (2.38) | 0.712 (3.47) | 0.805 (5.12) | 0.868 (7.57) | 0.898 (9.83) | 0.903 (10.31) | 0.911 (11.23) |
| **Rule Breaking** | 0.015 (1.01) | 0.024 (1.02) | 0.043 (1.05) | 0.086 (1.09) | 0.186 (1.23) | 0.392 (1.64) | 0.653 (2.88) | 0.814 (5.39) | 0.856 (6.96) | 0.876 (8.08) | 0.919 (12.31) | 0.936 (15.73) | 0.937 (15.9) |
| **Aggressive Problems** | 0.014 (1.01) | 0.038 (1.04) | 0.102 (1.11) | 0.253 (1.34) | 0.502 (2.01) | 0.739 (3.83) | 0.865 (7.43) | 0.912 (11.39) | 0.932 (14.69) | 0.947 (18.97) | 0.955 (22.3) | 0.952 (20.78) | 0.945 (18.08) |
| **Total Problems** | 0.3  (1.43) | 0.433  (1.76) | 0.586  (2.14) | 0.732  (3.73) | 0.843  (6.39) | 0.913  (11.5) | 0.950  (20.1) | 0.968  (31.7) | 0.978  (44.55) | 0.982  (56.91) | 0.985  (64.64) | 0.986  (69.8) | 0.986  (70.18) |

*Note.* Reliability is shown as the first value, and the corresponding information value in brackets underneath. Red highlights indicate points where reliability is low.


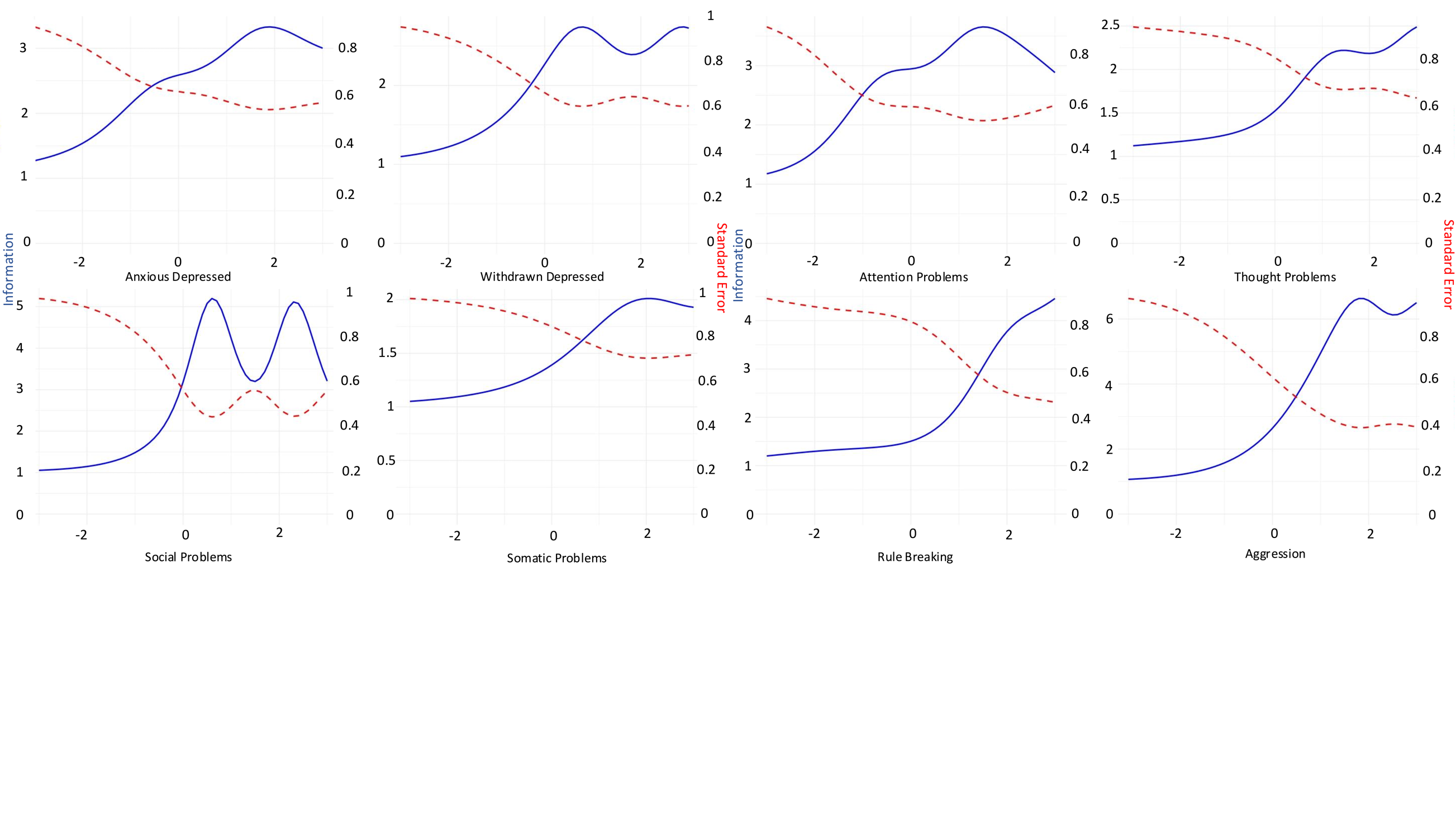

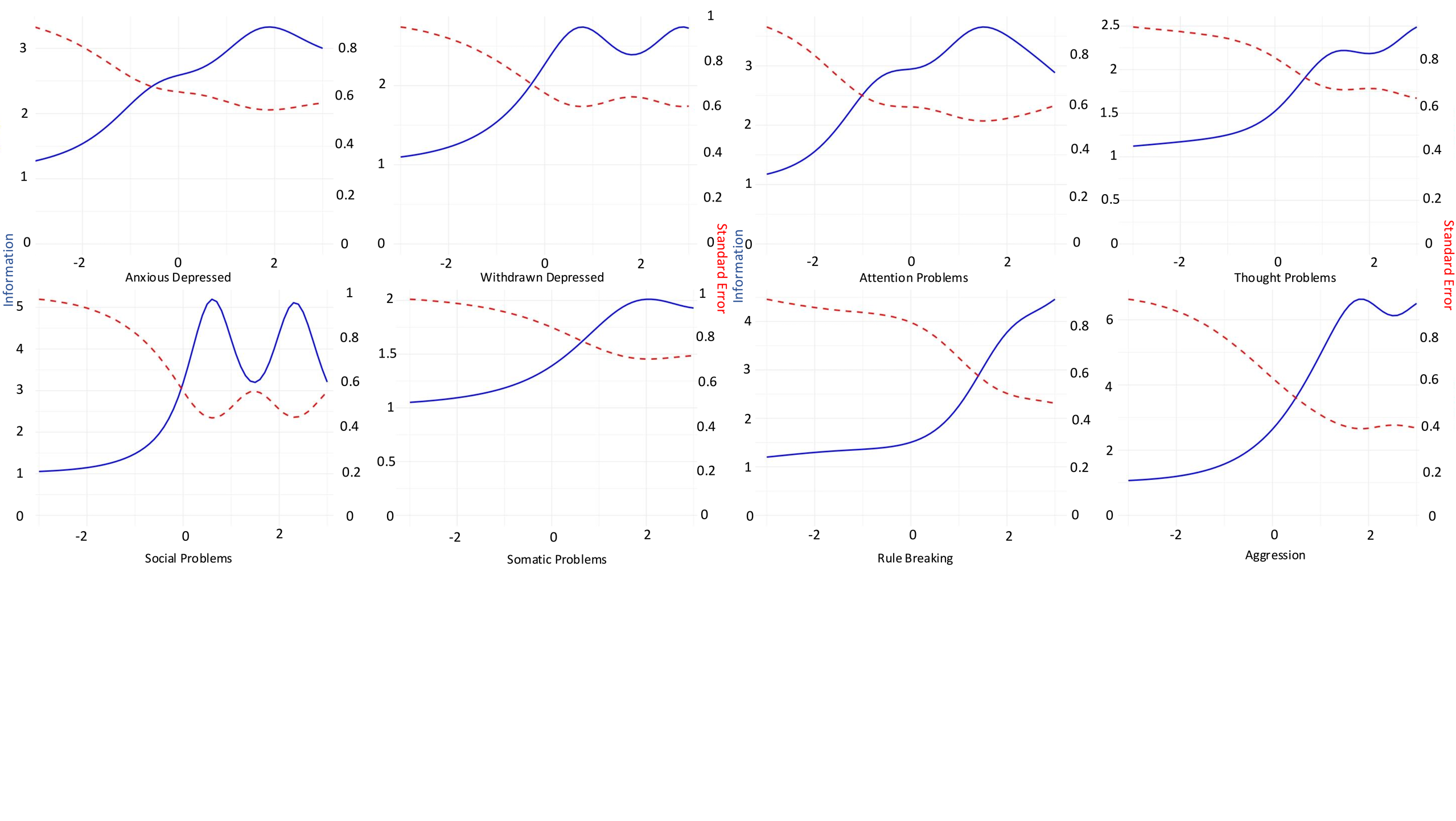
*Supplementary Figure 5.* Total Information Function / Curve for each subscales after graded response models were fit on the reduced item set with zero-inflated participants removed. Blue solid lines indicate information (plotted on the left y-axis) while red dashed lines indicate standard error (*SE* = $\frac{1}{\sqrt{I}}$) (plotted on the right y-axis).

*Supplementary Table 12.* Reliability of each subscale (*r_xx_* = 1 - $\frac{1}{I}$) along the latent trait continuum after inspection of the total information function (TIF) after removal of items and ZI participants for each subscale.

| **Scale** | **Reliability (r_xx_) across the latent Trait Continuum (**θ**)** | | | | | | | | | | | | |
| --- | --- | --- | --- | --- | --- | --- | --- | --- | --- | --- | --- | --- | --- |
|  | **-3.0 *SD*** | **-2.5 *SD*** | **-2.0 *SD*** | **-1.5 *SD*** | **-1.0**  ***SD*** | **-0.5 *SD*** | ***M*** | **0.5 SD** | **1.0 *SD*** | **1.5 *SD*** | **2.0 *SD*** | **2.5 *SD*** | **3.0 *SD*** |
| **Anxious Depressed** | 0.213 (1.27) | 0.272 (1.37) | 0.35 (1.54) | 0.444 (1.78) | 0.533 (2.14) | 0.591 (2.44) | 0.613 (2.59) | 0.630 (2.71) | 0.662 (2.96) | 0.692 (3.24) | 0.7 (3.32) | 0.685 (3.17) | 0.667 (3.0) |
| **Withdrawn Depressed** | 0.09 (1.1) | 0.128 (1.15) | 0.182 (1.22) | 0.257 (1.35) | 0.351 (1.54) | 0.457 (1.84) | 0.56 (2.27) | 0.625 (2.66) | 0.629 (2.7) | 0.593 (2.46) | 0.585 (2.41) | 0.62 (2.63) | 0.633 (2.72) |
| **Social Problems** | 0.051 (1.05) | 0.079 (1.09) | 0.127 (1.15) | 0.206 (1.26) | 0.325 (1.48) | 0.491 (1.96) | 0.686 (3.18) | 0.803 (5.07) | 0.764 (4.23) | 0.687 (3.12) | 0.772 (4.34) | 0.795 (4.89) | 0.688 (3.2) |
| **Somatic Problems** | 0.049 (1.05) | 0.065 (1.07) | 0.086 (1.09) | 0.116 (1.13) | 0.156 (1.19) | 0.211 (1.27) | 0.281 (1.39) | 0.36 (1.56) | 0.433 (1.76) | 0.484 (1.94) | 0.503 (2.01) | 0.495 (1.98) | 0.482 (1.93) |
| **Attention Problems** | 0.149 (1.17) | 0.235 (1.31) | 0.358 (1.56) | 0.496 (1.98) | 0.603 (2.52) | 0.652 (2.87) | 0.66 (2.94) | 0.677 (3.01) | 0.71 (3.45) | 0.726 (3.65) | 0.715 (3.51) | 0.689 (3.21) | 0.653 (2.88) |
| **Thought Problems** | 0.109 (1.12) | 0.128 (1.15) | 0.147 (1.17) | 0.169 (1.2) | 0.201 (1.25) | 0.256 (1.34) | 0.343 (1.52) | 0.45 (1.81) | 0.529 (2.12) | 0.550 (2.22) | 0.542 (2.18) | 0.563 (2.29) | 0.598 (2.49) |
| **Rule Breaking** | 0.167 (1.2) | 0.201 (1.25) | 0.229 (1.3) | 0.25 (1.33) | 0.266 (1.36) | 0.288 (1.4) | 0.351 (1.53) | 0.429 (1.75) | 0.557 (2.26) | 0.671 (3.04) | 0.735 (3.77) | 0.76 (4.16) | 0.776 (4.46) |
| **Aggressive Problems** | 0.063 (1.07) | 0.102 (1.11) | 0.163 (1.19) | 0.25 (1.33) | 0.365 (1.57) | 0.495 (1.98) | 0.621 (2.64) | 0.725 (3.64) | 0.798 (4.95) | 0.84 (6.26) | 0.848 (6.57) | 0.837 (6.13) | 0.846 (6.5) |

*Note.* Reliability is shown as the first value, and the corresponding information value in brackets underneath. Red highlights indicate points where reliability is low.


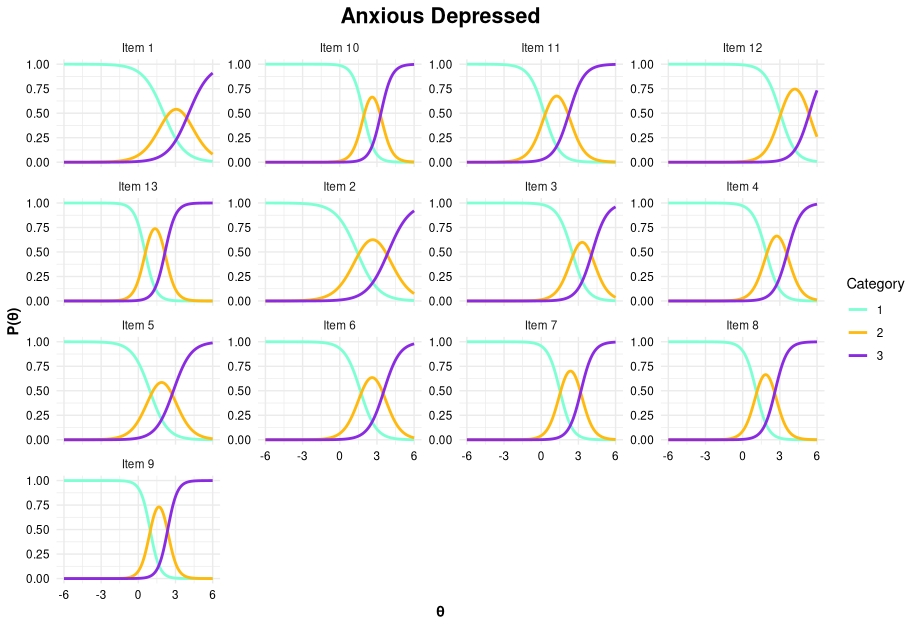


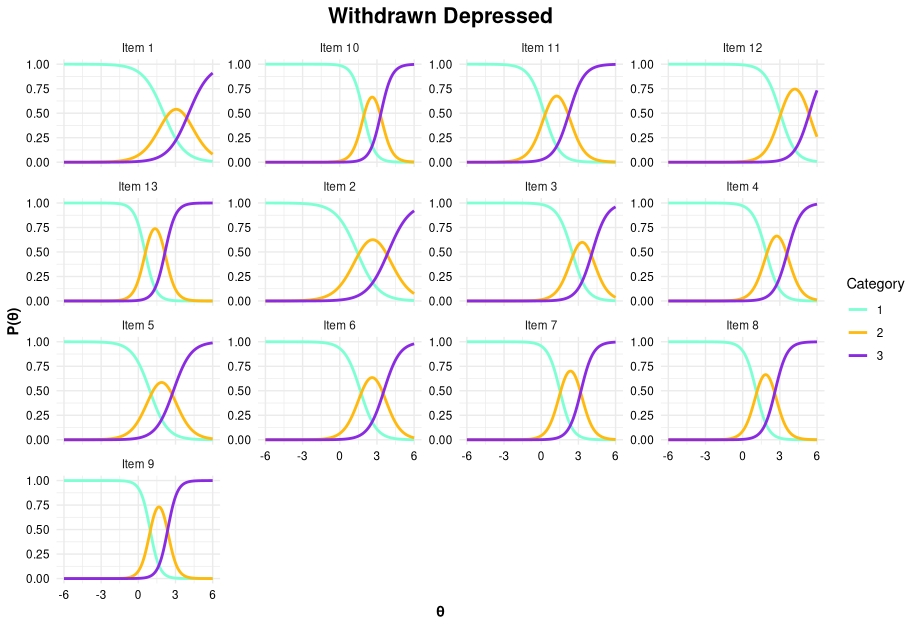


*Supplementary Figure 6.* Option Response Functions for each item under the Anxious Depressed & Withdrawn Depressed subscales. Graded response models were fit on the whole sample. The level (β) of the latent trait (θ), measured in standardised units, is plotted on the *x*-axis, while the probability of endorsing the latent trait is shown on the y-axis.


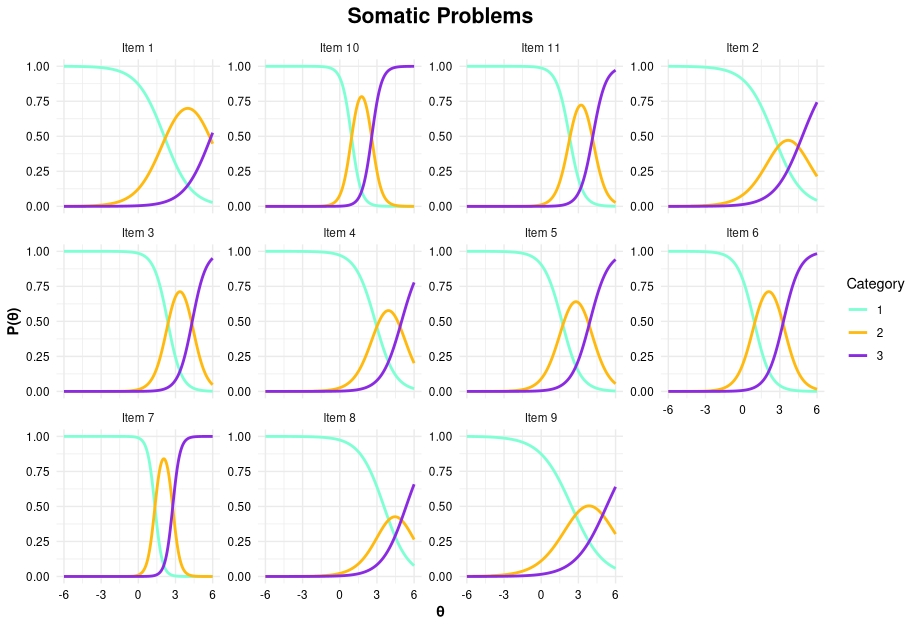


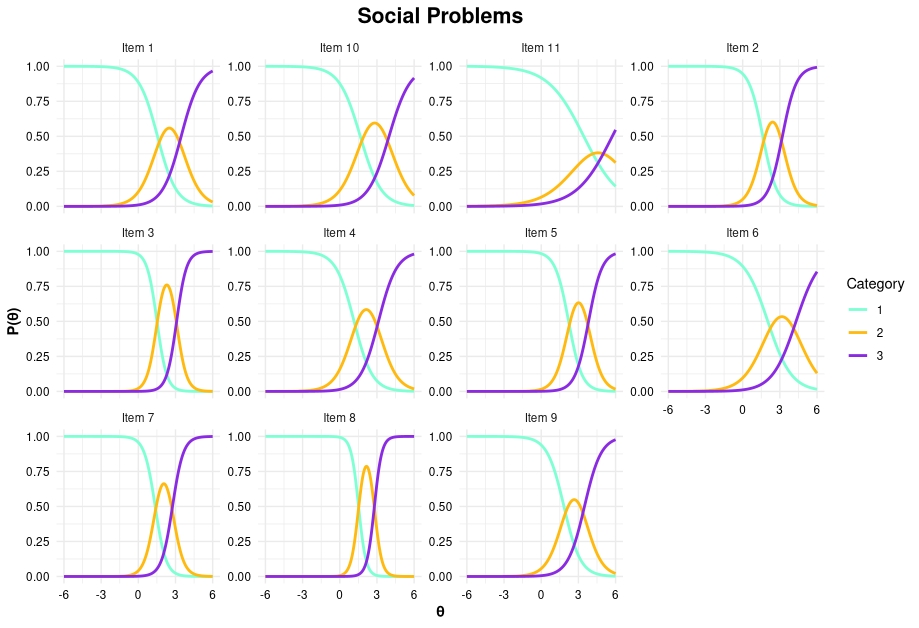


*Supplementary Figure 7.* Option Response Functions for each item under the Somatic Problems & Social Problems subscales. Graded response models were fit on the whole sample. The level (β) of the latent trait (θ), measured in standardised units, is plotted on the *x*-axis, while the probability of endorsing the latent trait is shown on the y-axis.


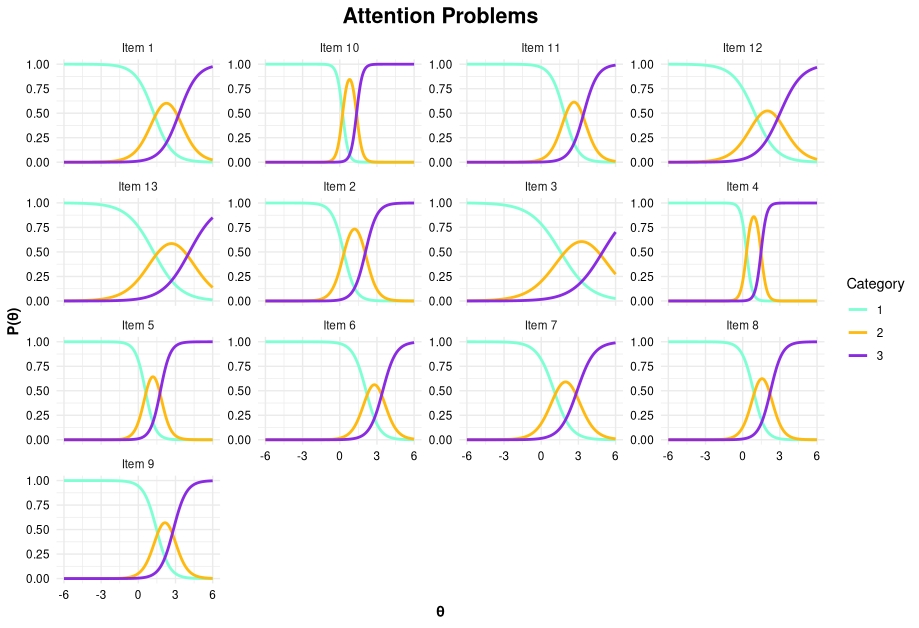


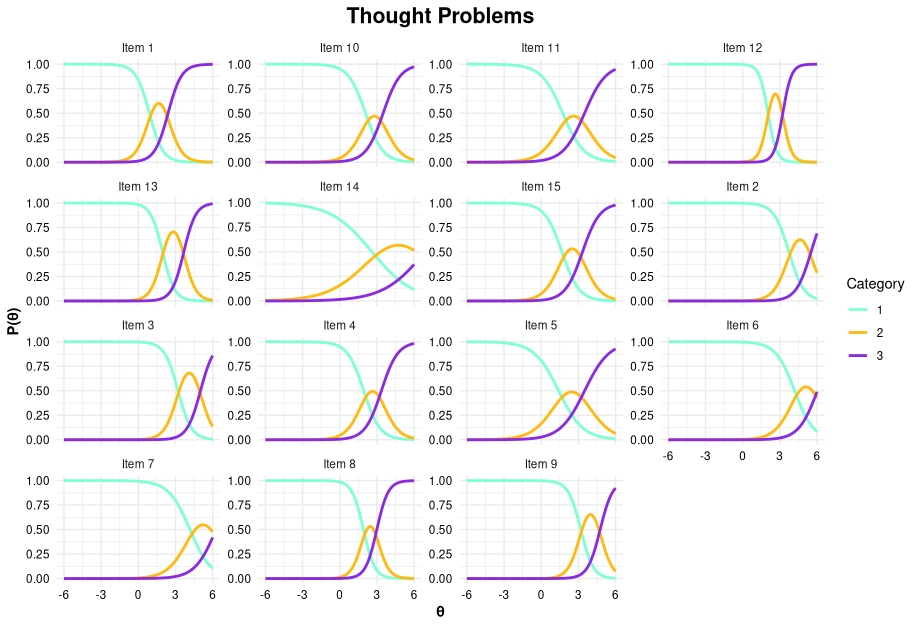


*Supplementary Figure 8.* Option Response Functions for each item under the Attention Problems & Thought problems subscales. Graded response models were fit on the whole sample. The level (β) of the latent trait (θ), measured in standardised units, is plotted on the x-axis, while the probability of endorsing the latent trait is shown on the y-axis.


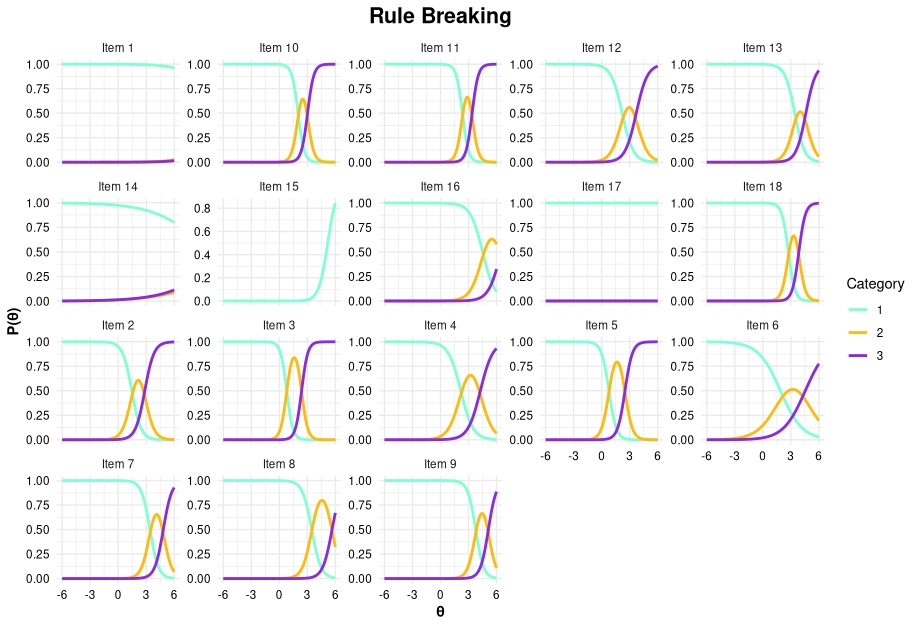


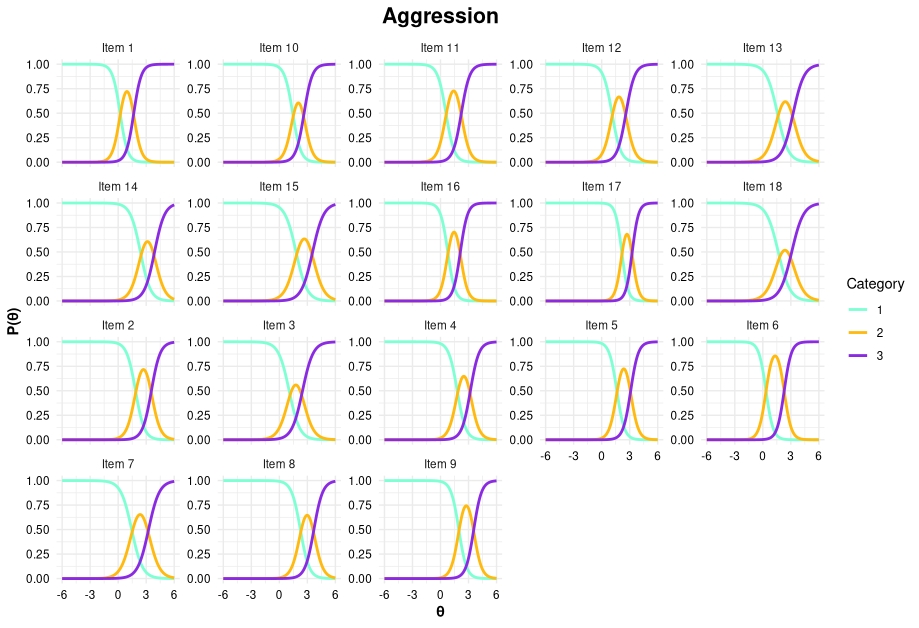


*Supplementary Figure 9.* Option Response Functions for each item under the Rule Breaking & Aggression subscales. Graded response models were fit on the whole sample. The level (β) of the latent trait (θ), measured in standardised units, is plotted on the x-axis, while the probability of endorsing the latent trait is shown on the y-axis.

*
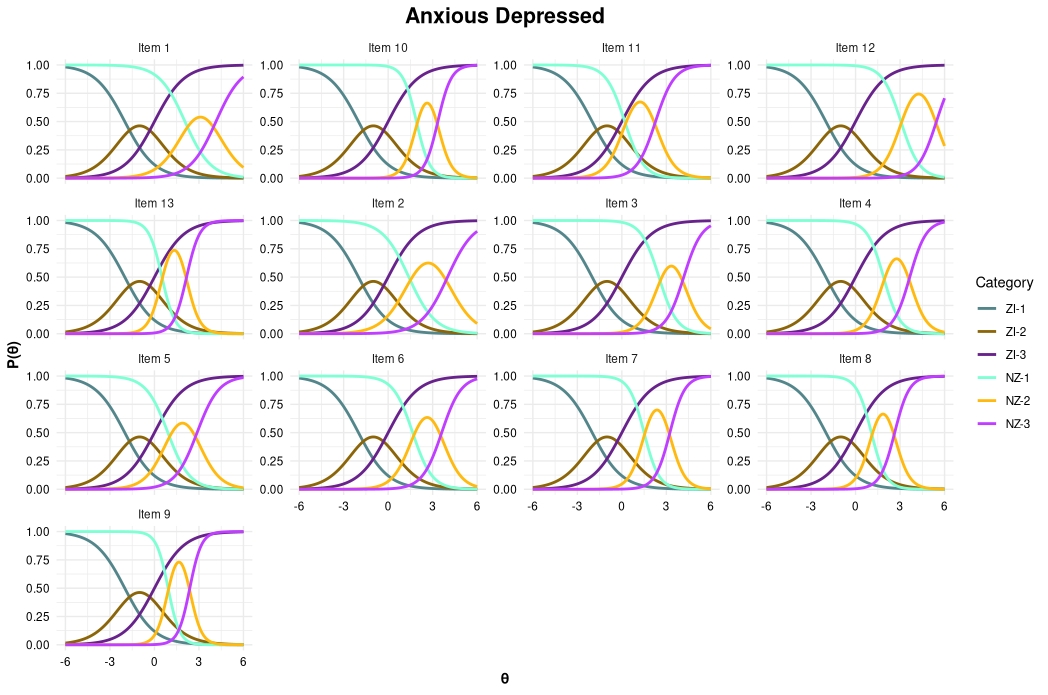
*

*
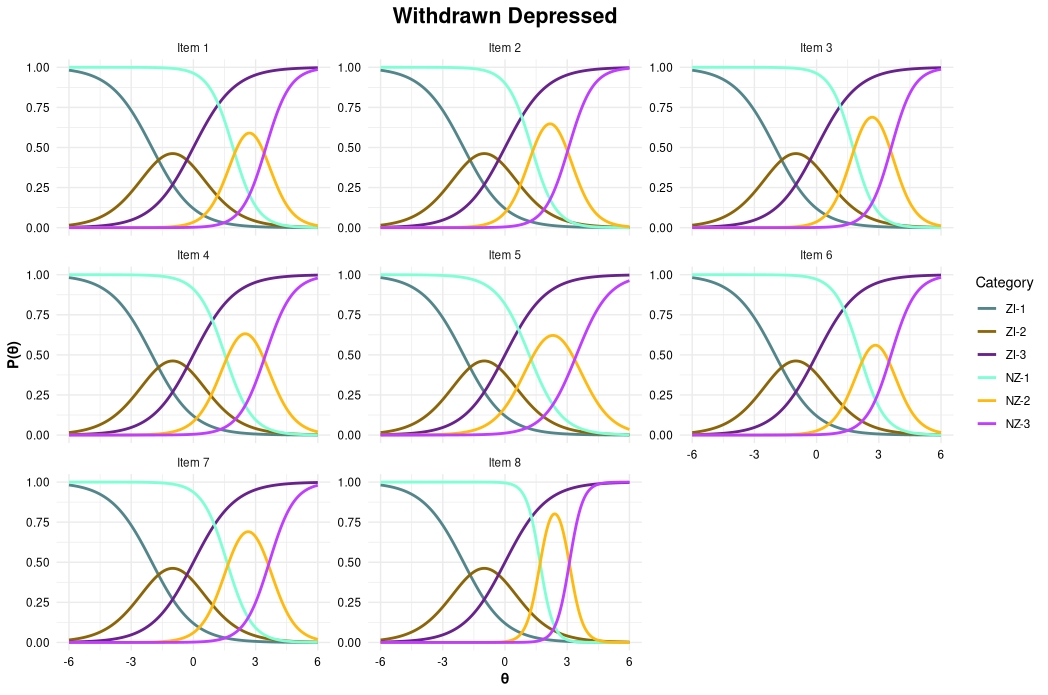
*

*Supplementary Figure 10.* Option Response Functions for each item under the Anxious Depressed & Withdrawn Depressed subscales fit with a zero-inflated graded response model. The zero-inflated class is notated with ZI in the legend, while NZ refers to the non-zero-inflated class. The level (β) of the latent trait (θ), measured in standardised units, is plotted on the *x*-axis, while the probability of endorsing the latent trait is shown on the *y*-axis.


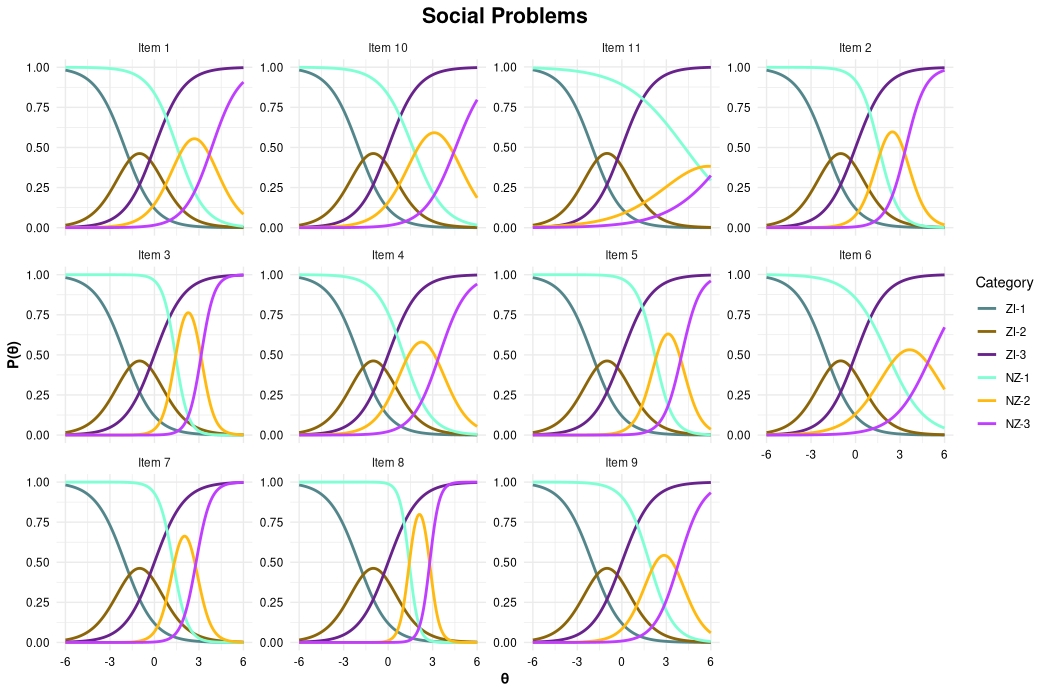


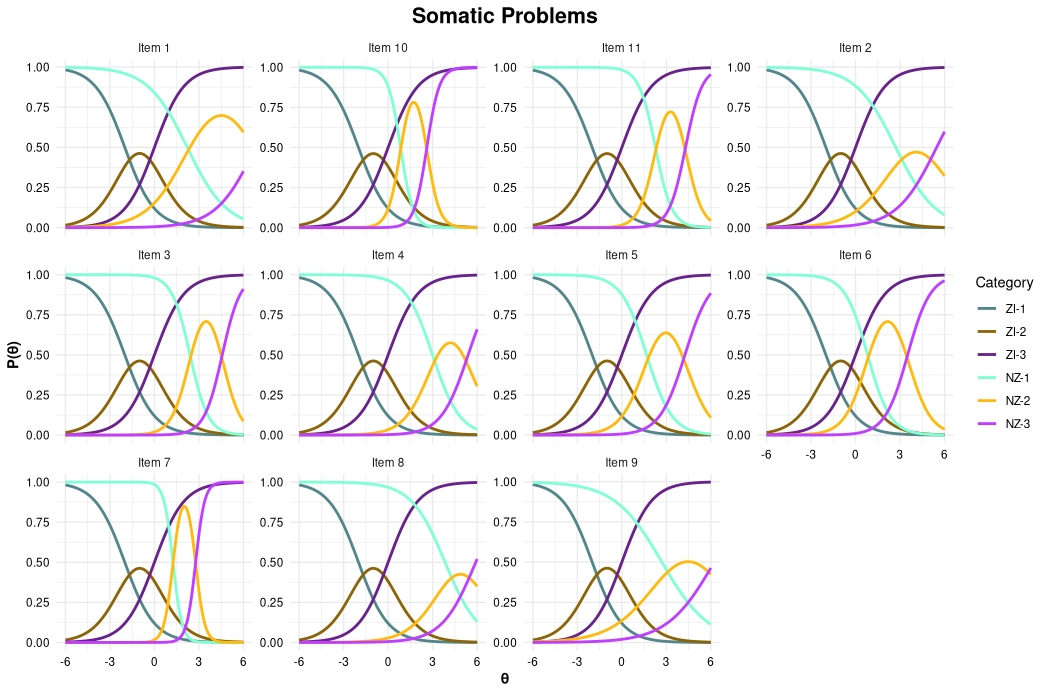


*Supplementary Figure 11.* Option Response Functions for each item under the Social Problems & Somatic Problems subscales fit with a zero-inflated graded response model. The zero-inflated class is notated with ZI in the legend, while NZ refers to the non-zero-inflated class. The level (β) of the latent trait (θ), measured in standardised units, is plotted on the *x*-axis, while the probability of endorsing the latent trait is shown on the *y*-axis.


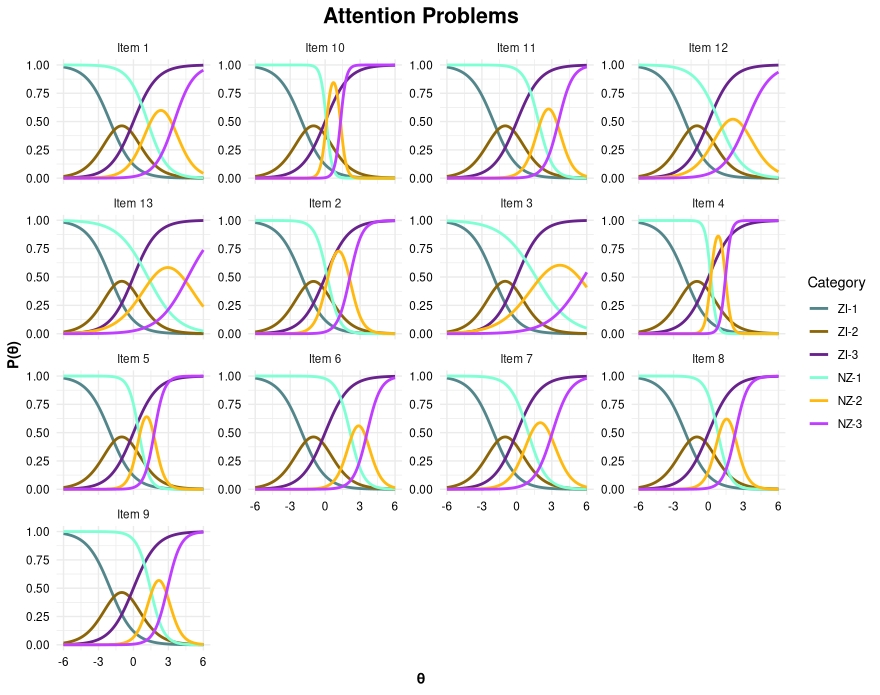


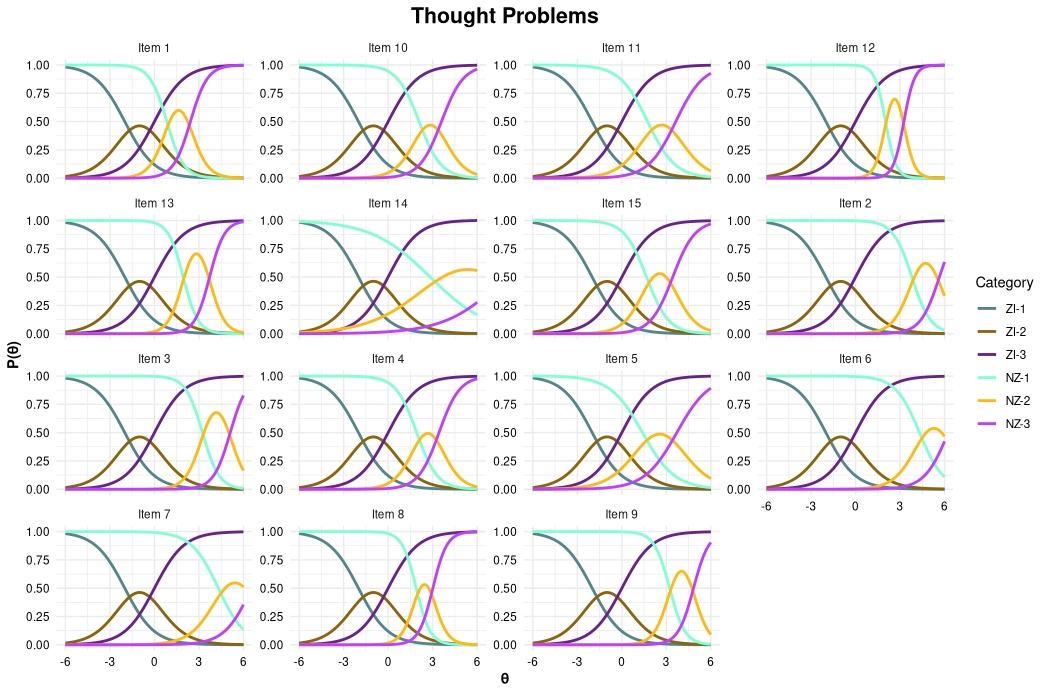


*Supplementary Figure 12.* Option Response Functions for each item under the Attention Problems & Thought Problems subscales fit with a zero-inflated graded response model. The zero-inflated class is notated with ZI in the legend, while NZ refers to the non-zero-inflated class. The level (β) of the latent trait (θ), measured in standardised units, is plotted on the *x*-axis, while the probability of endorsing the latent trait is shown on the *y*-axis.

*
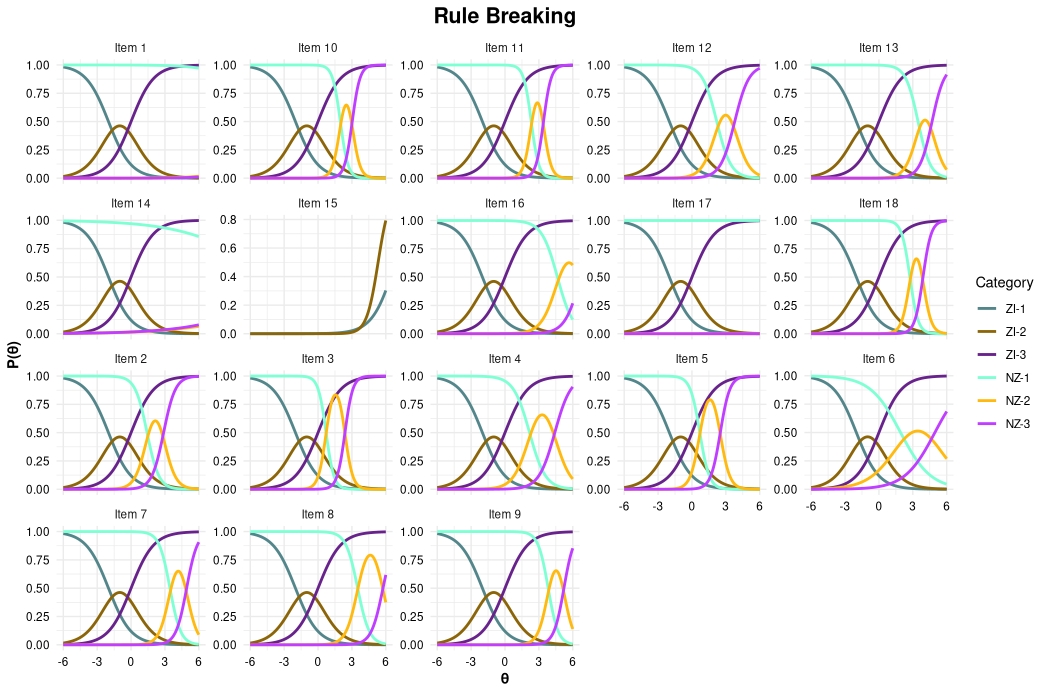
*

*
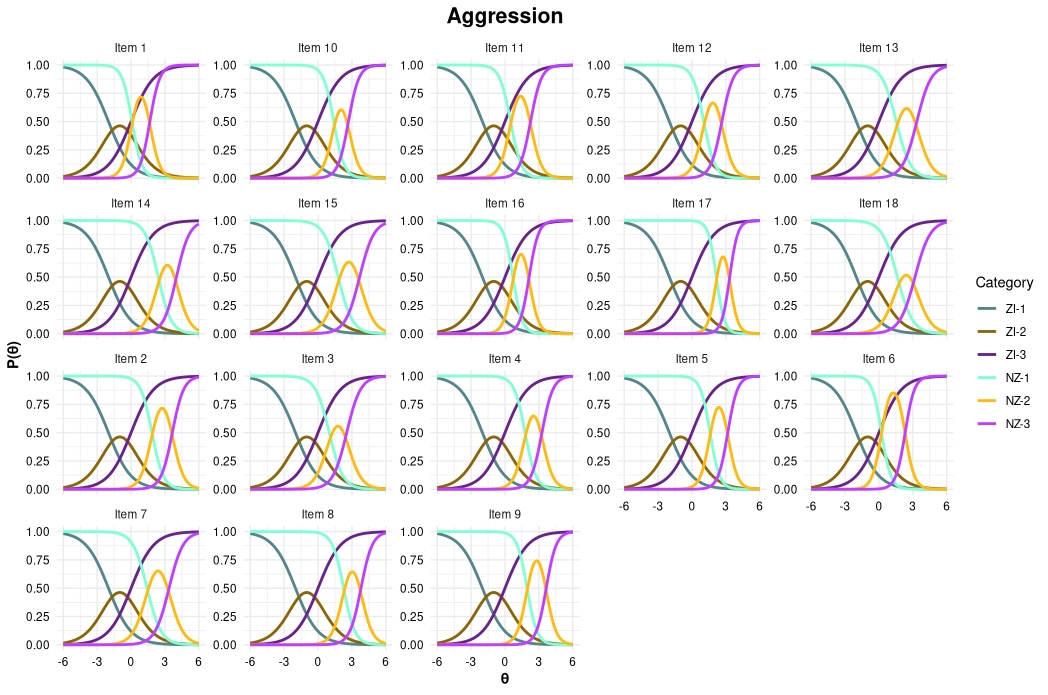
*

*Supplementary Figure 13.* Option Response Functions for each item under the Rule Breaking & Aggression subscales fit with a zero-inflated graded response model. The zero-inflated class is notated with ZI in the legend, while NZ refers to the non-zero-inflated class. The level (β) of the latent trait (θ), measured in standardised units, is plotted on the *x*-axis, while the probability of endorsing the latent trait is shown on the *y*-axis.


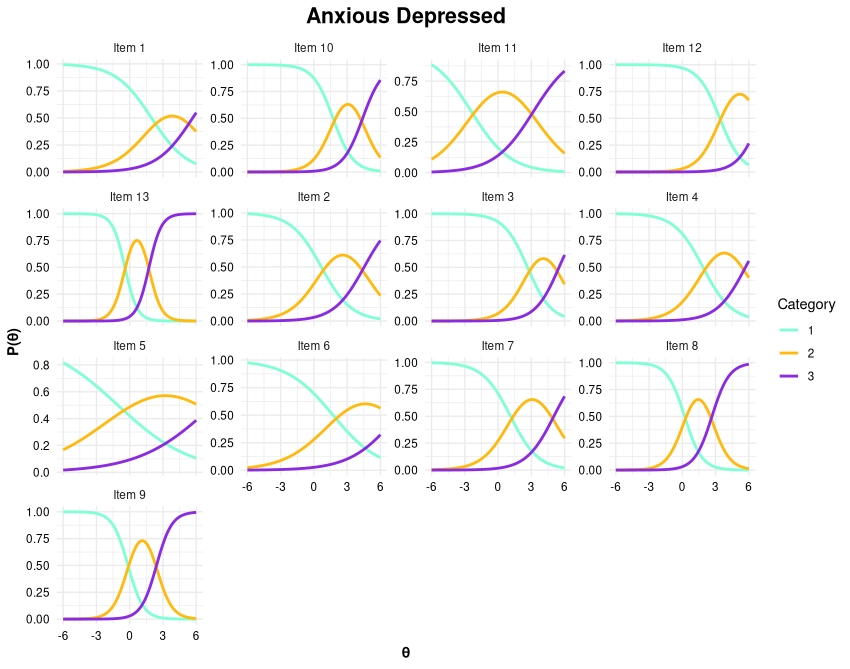


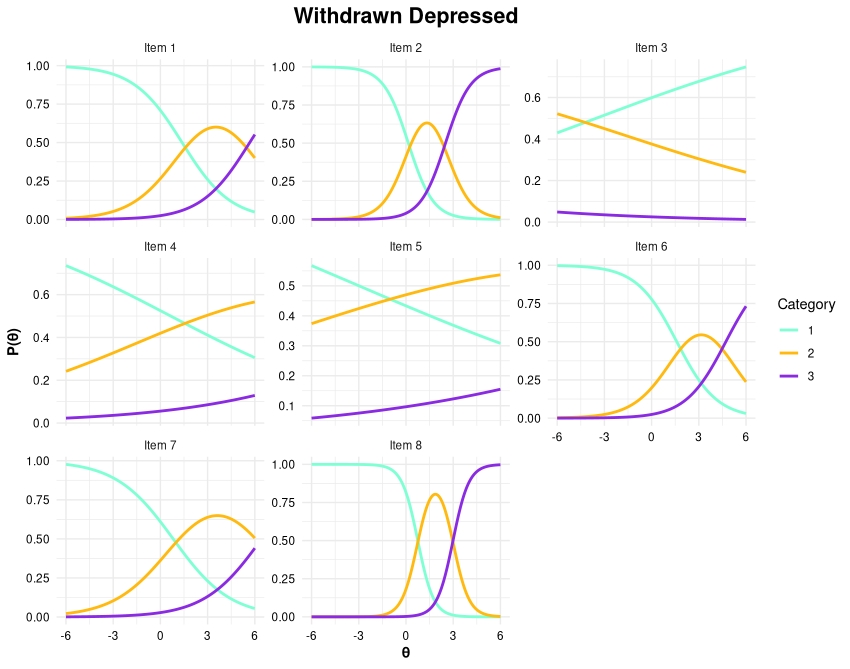


*Supplementary Figure 14.* Option Response Functions for each item under the Anxious Depressed & Withdrawn Depressed subscales after the zero inflated component has been manually removed. The level (β) of the latent trait (θ), measured in standardised units, is plotted on the *x*-axis, while the probability of endorsing the latent trait is shown on the *y*-axis.

*
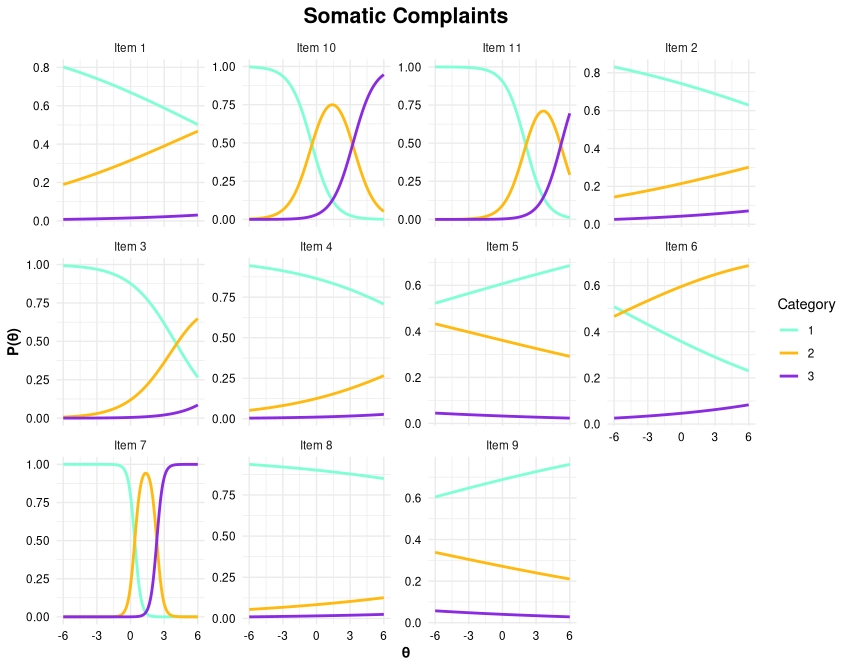
*

*
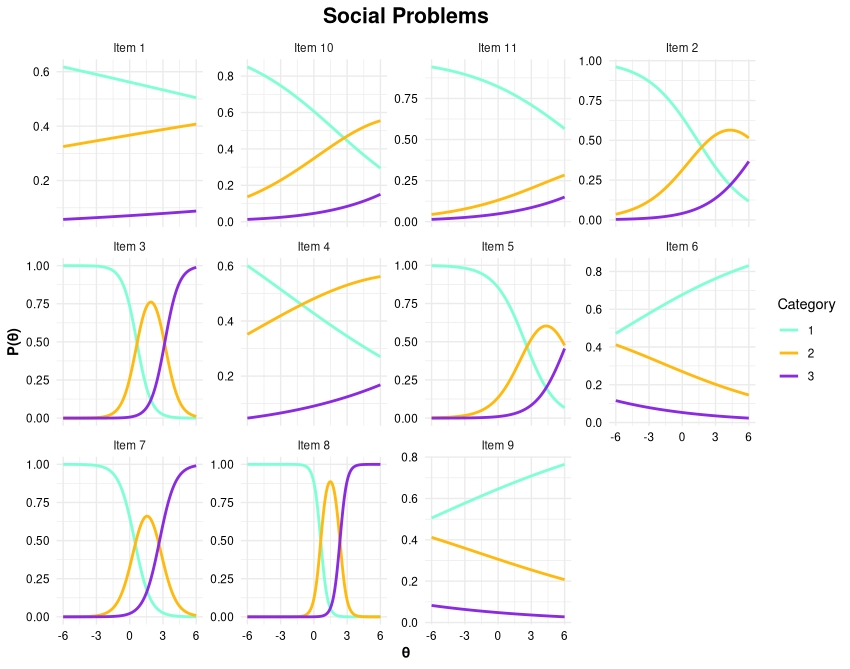
*

*Supplementary Figure 15.* Option Response Functions for each item under the Somatic Problems & Social Problems subscales after the zero inflated component has been manually removed. The level (β) of the latent trait (θ), measured in standardised units, is plotted on the *x*-axis, while the probability of endorsing the latent trait is shown on the *y*-axis.

*
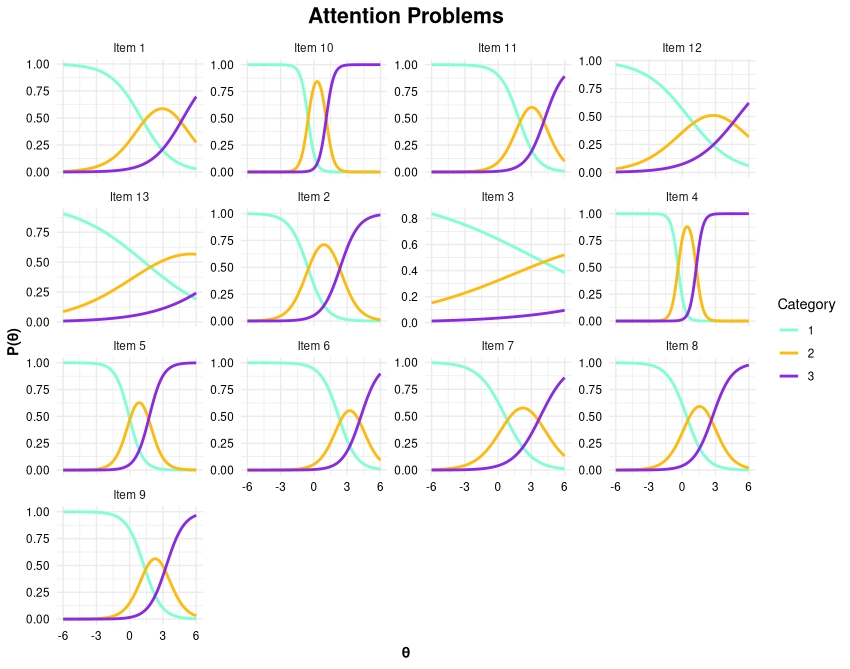
*

*
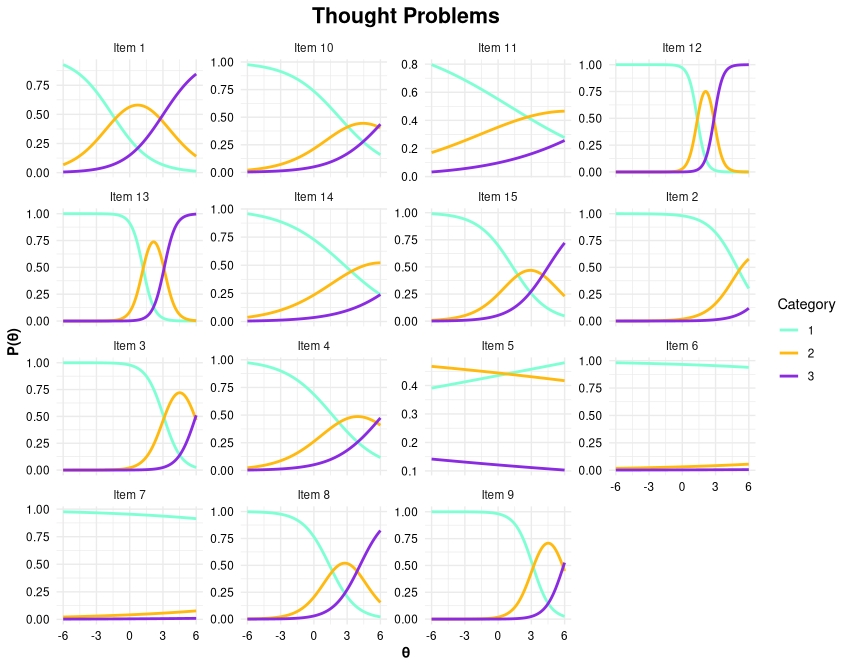
*

*Supplementary Figure 16.* Option Response Functions for each item under the Attention Problems & Thought Problems subscales after the zero inflated component has been manually removed. The level (β) of the latent trait (θ), measured in standardised units, is plotted on the *x*-axis, while the probability of endorsing the latent trait is shown on the *y*-axis.

*
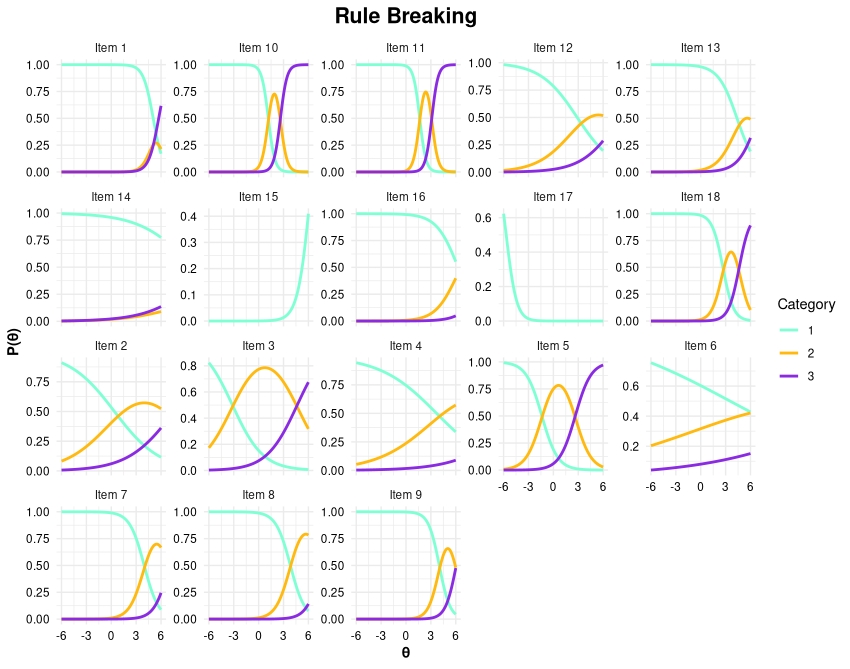
*

*
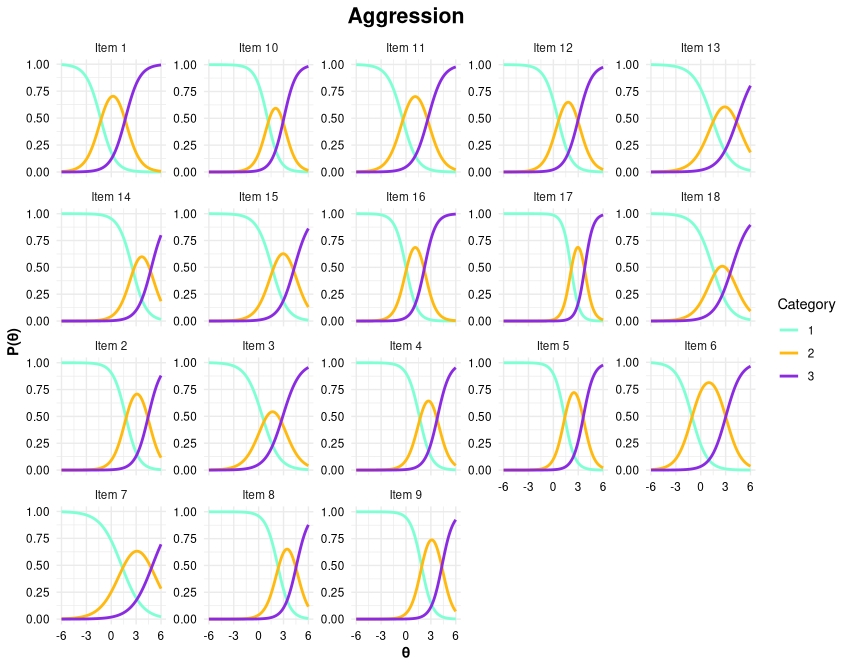
*

*Supplementary Figure 17.* Option Response Functions for each item under the Rule Breaking & Aggression subscales after the zero inflated component has been manually removed. The level (β) of the latent trait (θ), measured in standardised units, is plotted on the *x*-axis, while the probability of endorsing the latent trait is shown on the *y*-axis.


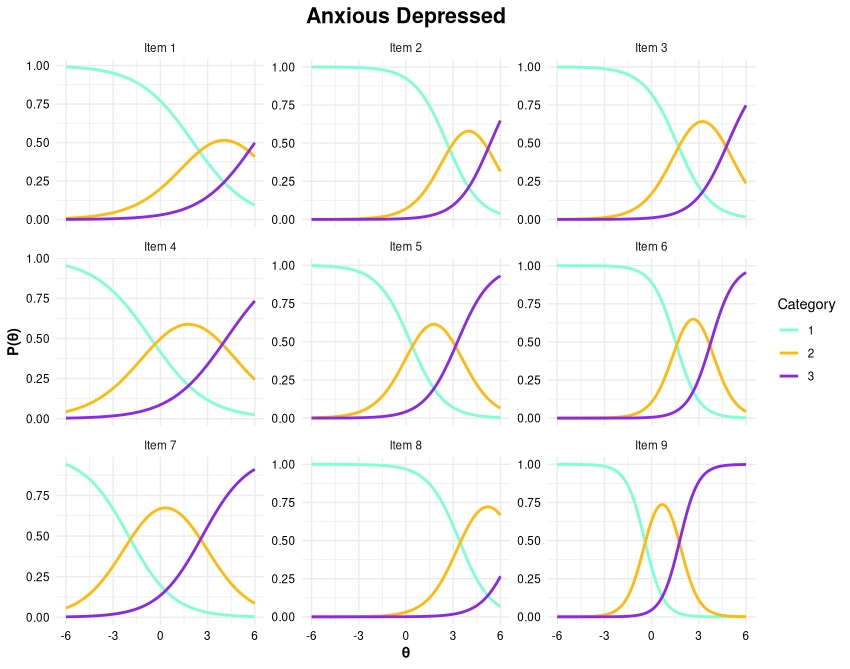


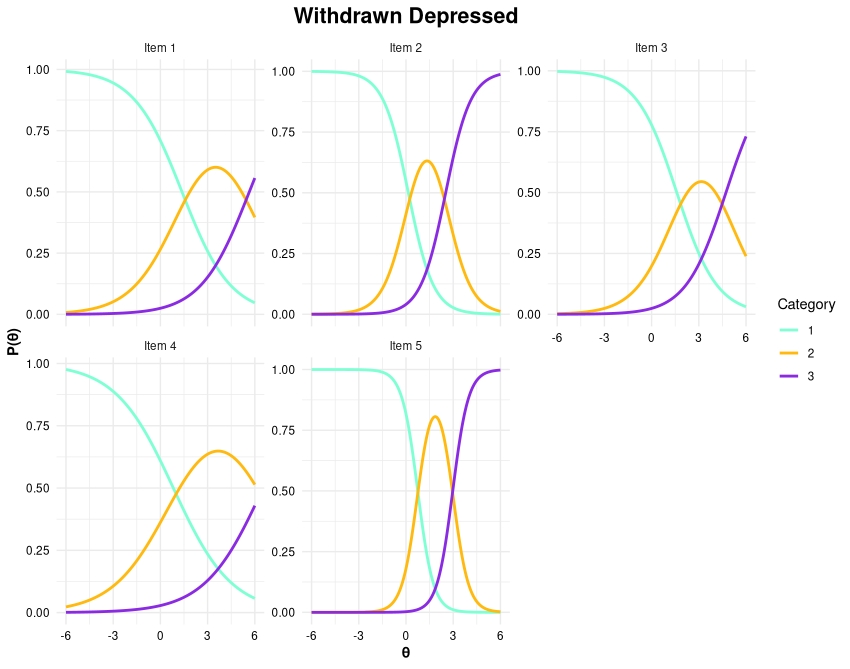


*Supplementary Figure 18.* Option Response Functions for each item under the Anxious Depressed & Withdrawn Depressed subscales after the zero inflated component had been manually removed and items were removed if they showed low discriminability (in terms of low slope parameters (α), high overlap with other items, and/or violations of the functional form assumption (𝑆− χ²>0.01). The level (β) of the latent trait (θ), measured in standardised units, is plotted on the *x*-axis, while the probability of endorsing the latent trait is shown on the *y*-axis.

*
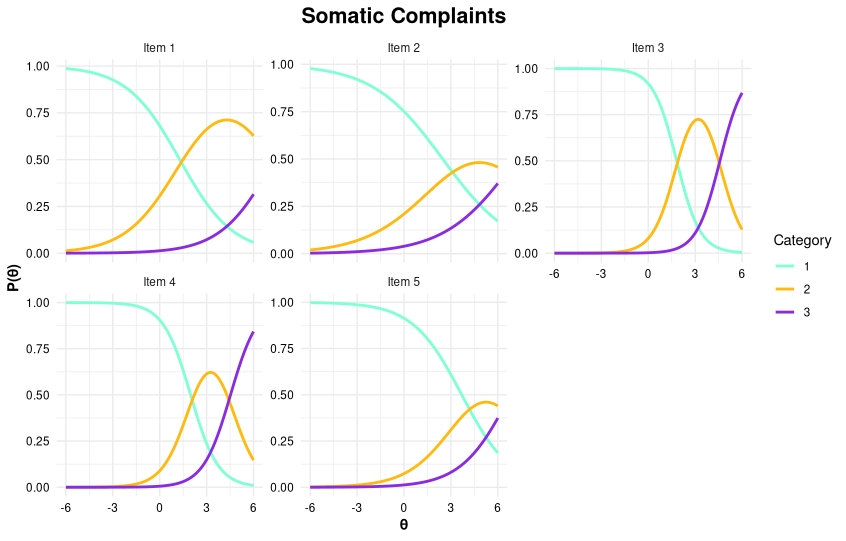
*

*
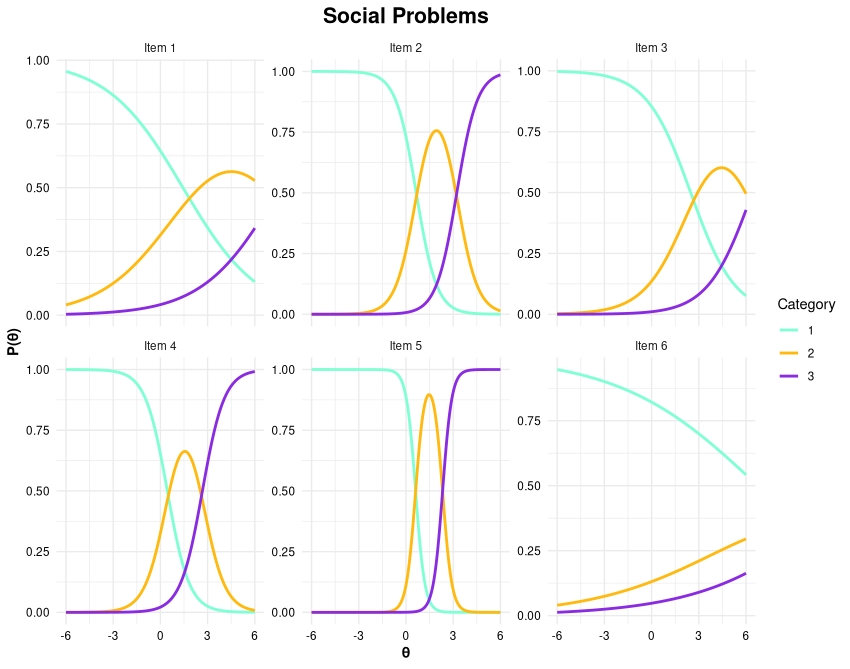
*

*Supplementary Figure 19.* Option Response Functions for each item under the Somatic Problems & Social Problems subscales after the zero inflated component had been manually removed and items were removed if they showed low discriminability (in terms of low slope parameters (α), high overlap with other items, and/or violations of the functional form assumption (𝑆− χ²>0.01). The level (β) of the latent trait (θ), measured in standardised units, is plotted on the *x*-axis, while the probability of endorsing the latent trait is shown on the *y*-axis.

*
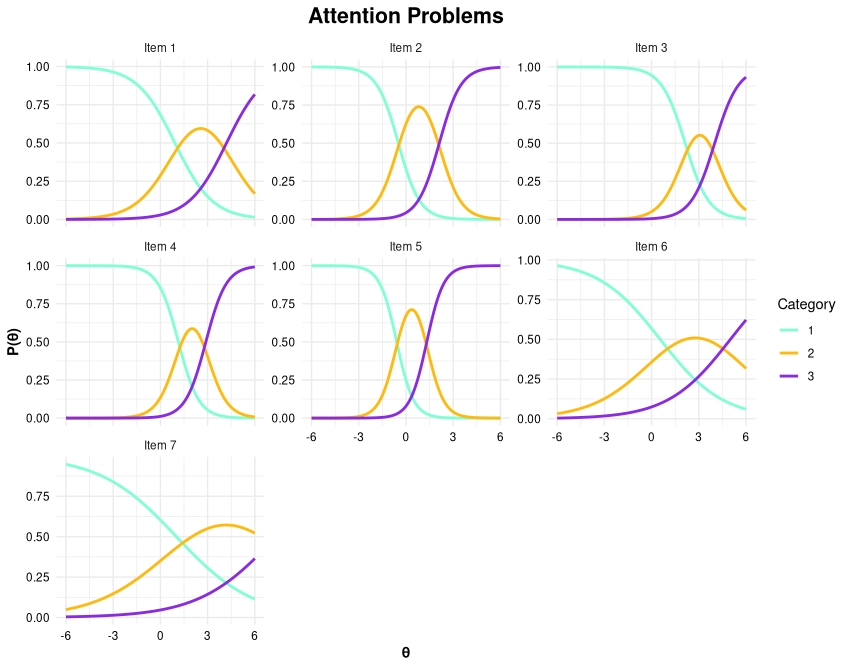
*

*
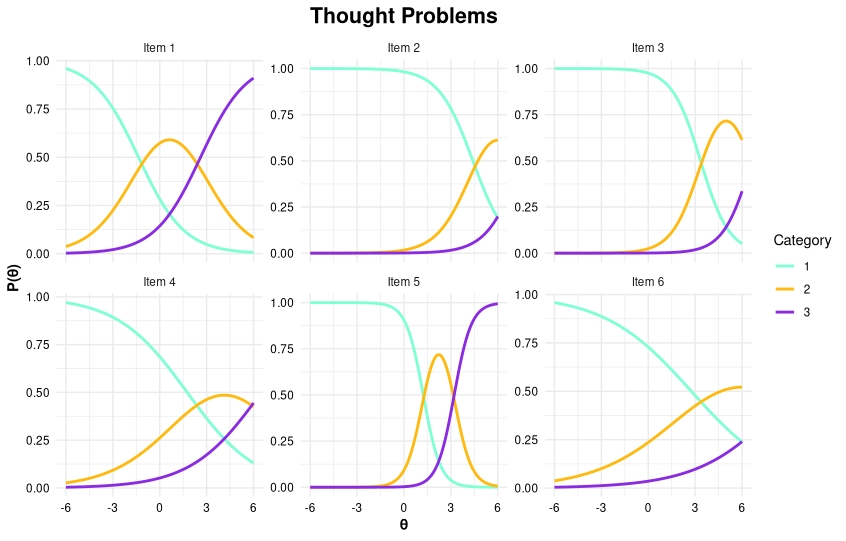
*

*Supplementary Figure 20.* Option Response Functions for each item under the Attention Problems & Thought Problems subscales after the zero inflated component had been manually removed and items were removed if they showed low discriminability (in terms of low slope parameters (α), high overlap with other items, and/or violations of the functional form assumption (𝑆− χ² *p* >0.01). The level (β) of the latent trait (θ), measured in standardised units, is plotted on the *x*-axis, while the probability of endorsing the latent trait is shown on the *y*-axis.

*
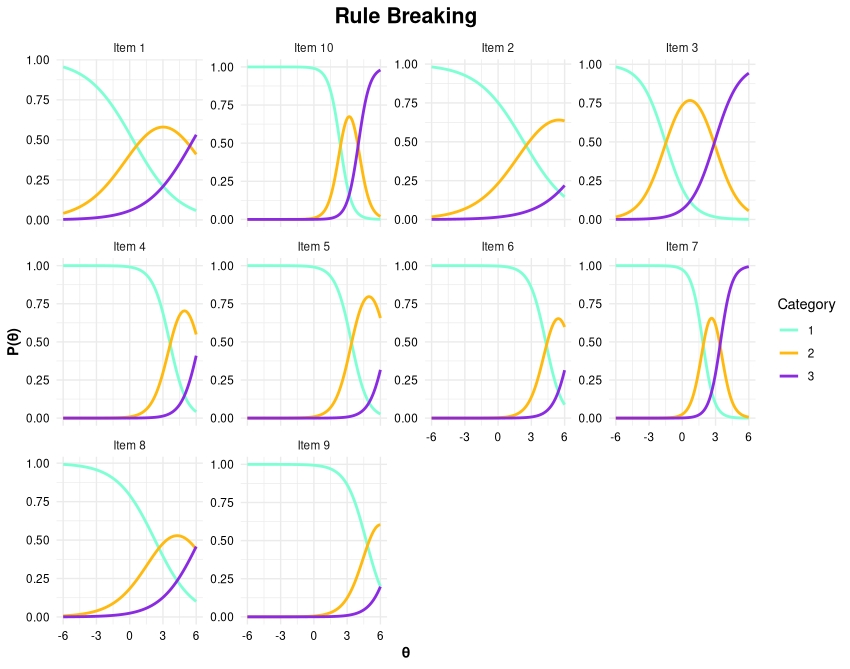
*

*
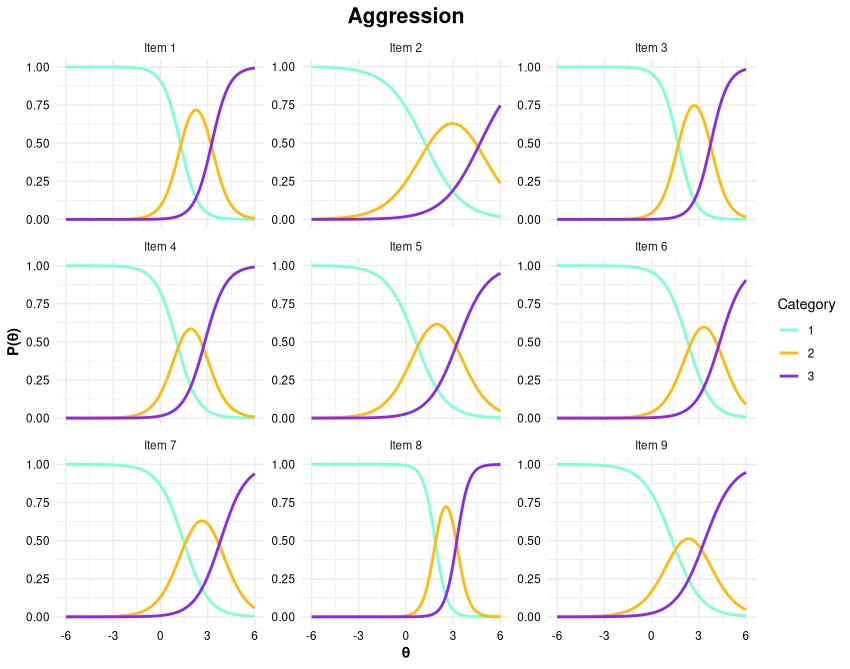
*

*Supplementary Figure 21.* Option Response Functions for each item under the Rule Breaking & Aggression subscales after the zero inflated component had been manually removed and items were removed if they showed low discriminability (in terms of low slope parameters (α), high overlap with other items, and/or violations of the functional form assumption (𝑆− χ² *p* >0.01). The level (β) of the latent trait (θ), measured in standardised units, is plotted on the *x*-axis, while the probability of endorsing the latent trait is shown on the *y*-axis.

1. ***Exploratory Structural Equation Models.***

*Supplementary Table 13.* Fit indices from Exploratory Structural Equation Models (ESEM) fit on 107 items from all subscales.

| **Model** | **χ*^2^*** | ***df*** | ***p*** | ***RMSEA***  **(95% *CI*)** | ***SRMR*** | ***CFI*** |
| --- | --- | --- | --- | --- | --- | --- |
| **1-Factor** | 138,717.7 | 5,564 | <.001 | 0.045 (0.045, 0.045) | 0.215 | 0.755 |
| **2-Factor** | 72,857.24 | 5,458 | <.001 | 0.032 (0.032, 0.032) | 0.195 | 0.876 |
| **3-Factor** | 48,402.67 | 5,353 | <.001 | 0.026 (0.026, 0.026) | 0.174 | 0.921 |
| **4-Factor** | 35,213.57 | 5,249 | <.001 | 0.022 (0.022, 0.022) | 0.157 | 0.945 |
| **5-Factor** | 28,674.28 | 5,146 | <.001 | 0.02 (0.02, 0.019) | 0.152 | 0.957 |
| **6-Factor** | 24,530.22 | 5,044 | <.001 | 0.018 (0.018, 0.018) | 0.15 | 0.964 |
| **7-Factor** | 22,369.77 | 4,943 | <.001 | 0.017 (0.017, 0.017) | 0.124 | 0.968 |
| **8-Factor** | 21,663.96 | 4,843 | <.001 | 0.017 (0.017, 0.017) | 0.096 | 0.969 |

*Note.* χ*^2^*= chi-squared statistic, *p* = probability value of the χ*^2^*statistic, *df* = degrees of freedom, *RMSEA* = Root mean square error of approximation, *CFI* = comparative fit index, *SRMR* = Standardised root mean square residual.

*Supplementary Table 14.* Fit indices from Bifactor Exploratory Structural Equation Models (B-ESEM) fit on 107 items from all subscales.

| **Model** | **χ*^2^*** | ***df*** | ***p*** | ***RMSEA***  **(95% *CI*)** | ***SRMR*** | ***CFI*** | ***ECV*** | ***ωh*** |
| --- | --- | --- | --- | --- | --- | --- | --- | --- |
| **P + 1-Factor** | 72,857.24 | 5,458 | <.001 | 0.032 (0.032, 0.032) | 0.195 | 0.876 | 0.58 | 0.35 |
| **P + 2-Factors** | 48,402.67 | 5,353 | <.001 | 0.026 (0.026, 0.026) | 0.174 | 0.921 | 0.51 | 0.32 |
| **P + 3-Factors** | 35,213.57 | 5,249 | <.001 | 0.022 (0.022, 0.022) | 0.157 | 0.945 | 0.59 | 0.41 |
| **P + 4-Factors** | 28,674.06 | 5,146 | <.001 | 0.02 (0.02, 0.019) | 0.152 | 0.957 | 0.57 | 0.41 |
| **P + 5-Factors** | 24,546.13 | 5,044 | <.001 | 0.018 (0.018, 0.018) | 0.15 | 0.964 | 0.58 | 0.44 |
| **P + 6-Factors** | 22,372.39 | 4,943 | <.001 | 0.017 (0.017, 0.017) | 0.125 | 0.968 | 0.55 | 0.43 |

*Note.* χ*^2^*= chi-squared statistic, *p* = probability value of the χ*^2^*statistic, *df* = degrees of freedom, *RMSEA* = Root mean square error of approximation, *CFI* = comparative fit index, *SRMR* = Standardised root mean square residual, *ECV*=Explained Common Variance.


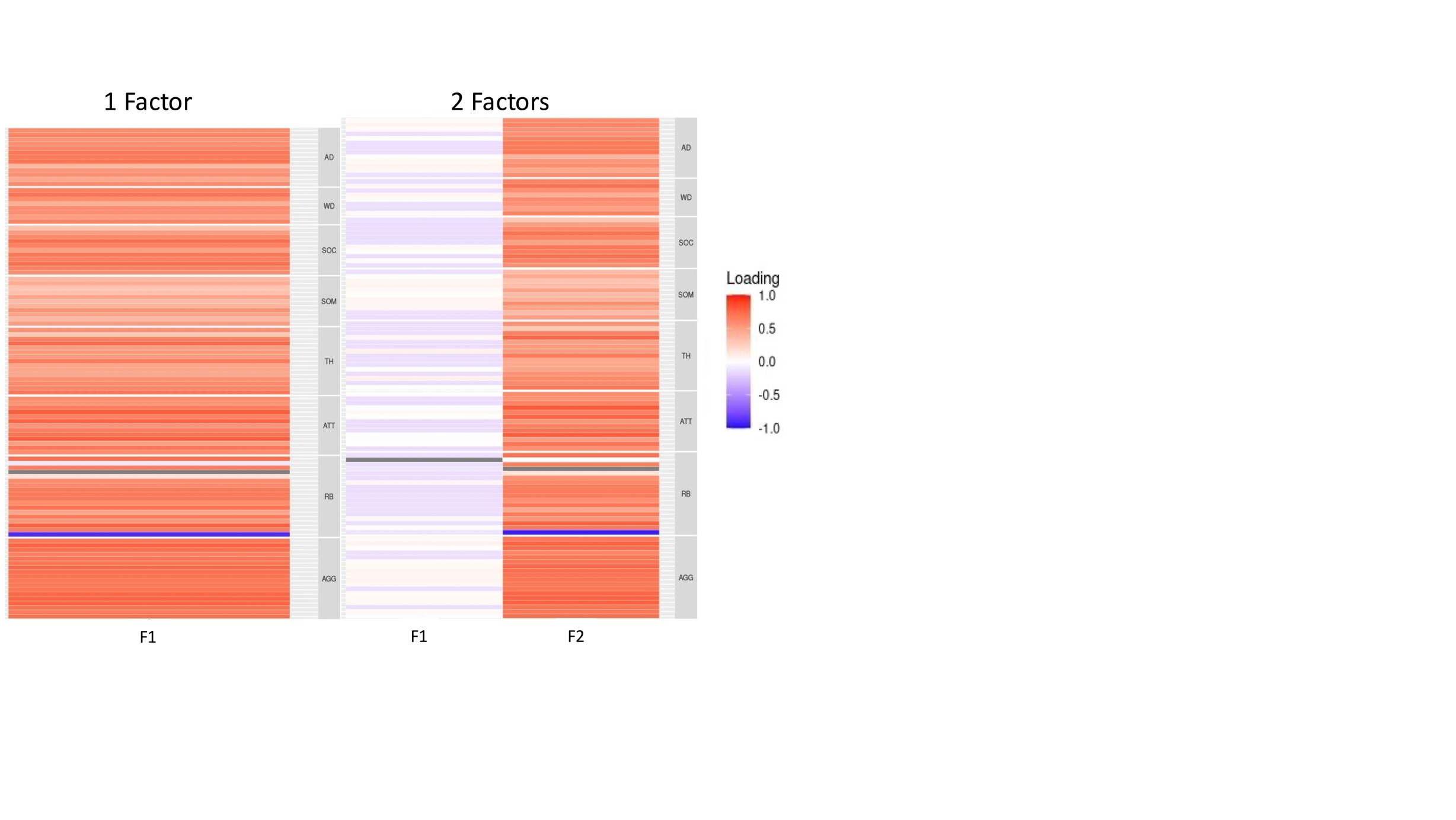

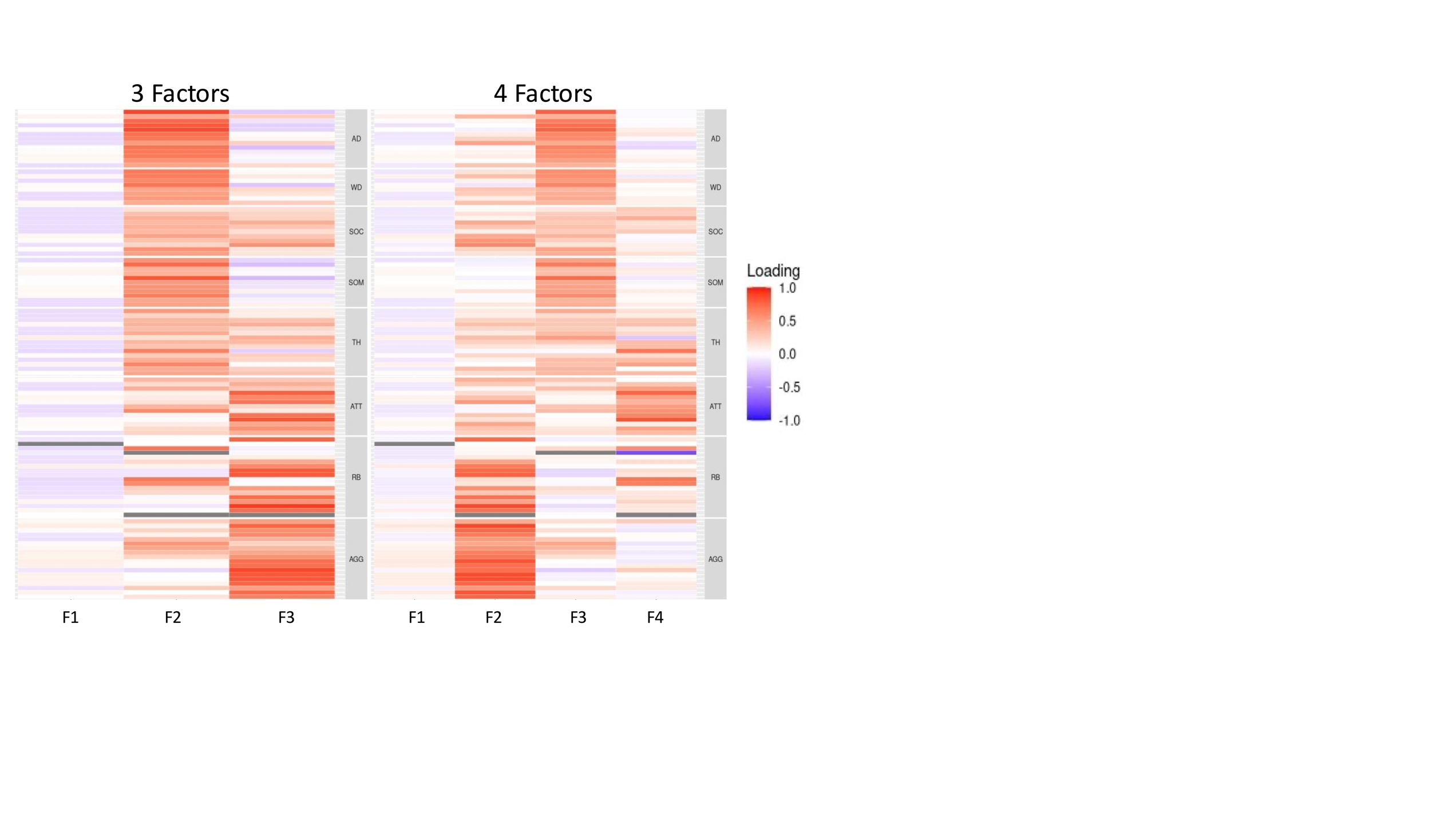
*Supplementary Figure 22.* Standardised loadings across all items in 1-4 ESEM factor solutions. Items are organised into their original subscales (AD = Anxious Depressed, WD = Withdrawn Depressed, SOC = Social Problems, SOM = Somatic Complaints, TH = Thought Problems, ATT = Attention Problems, RB = Rule Breaking, AGG = Aggressive Behaviours). Factors are plotted on the *x*-axis (F1 = Factor 1, F2 = Factor 2 etc.), items are plotted on the *y*-axis


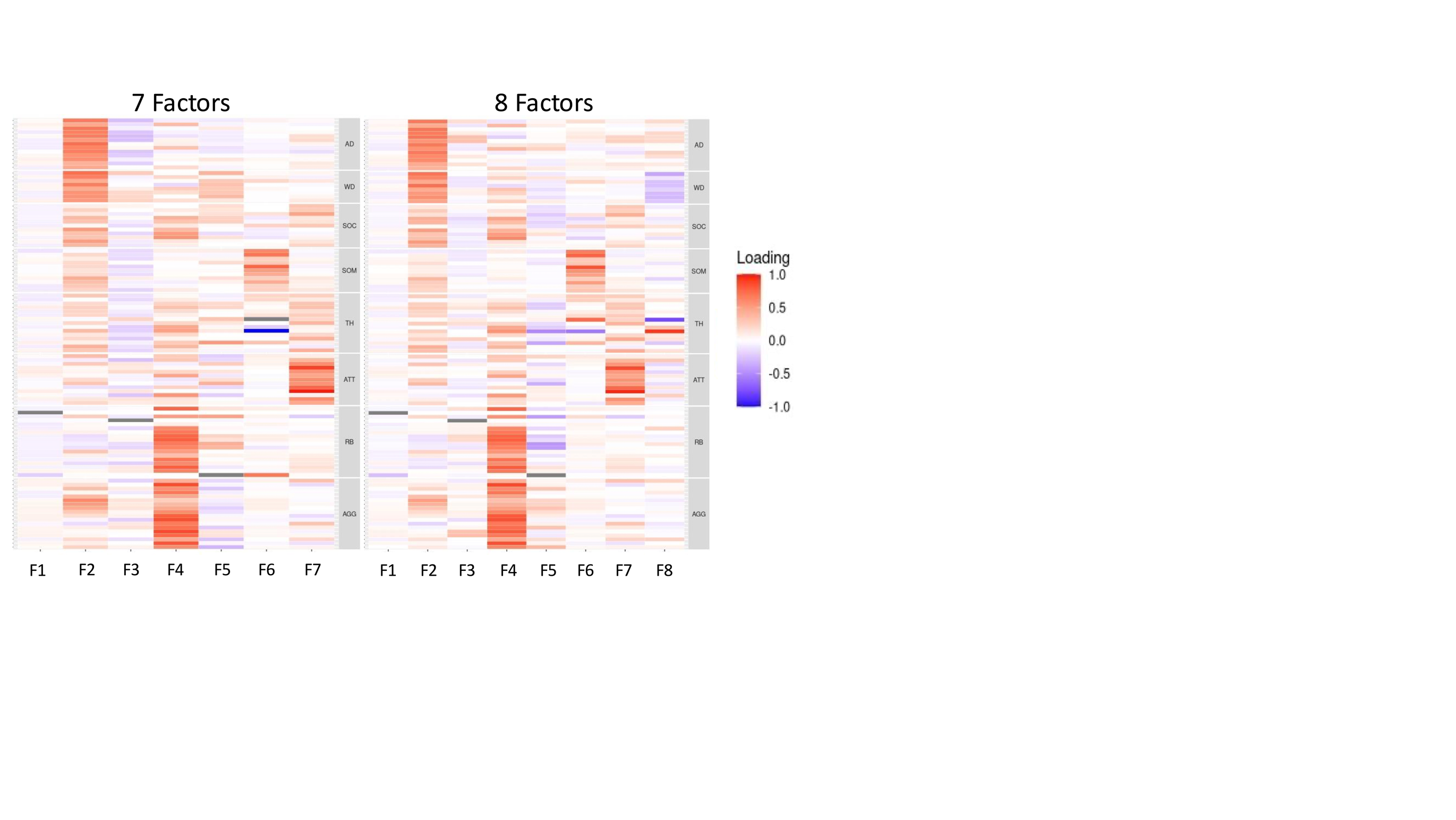

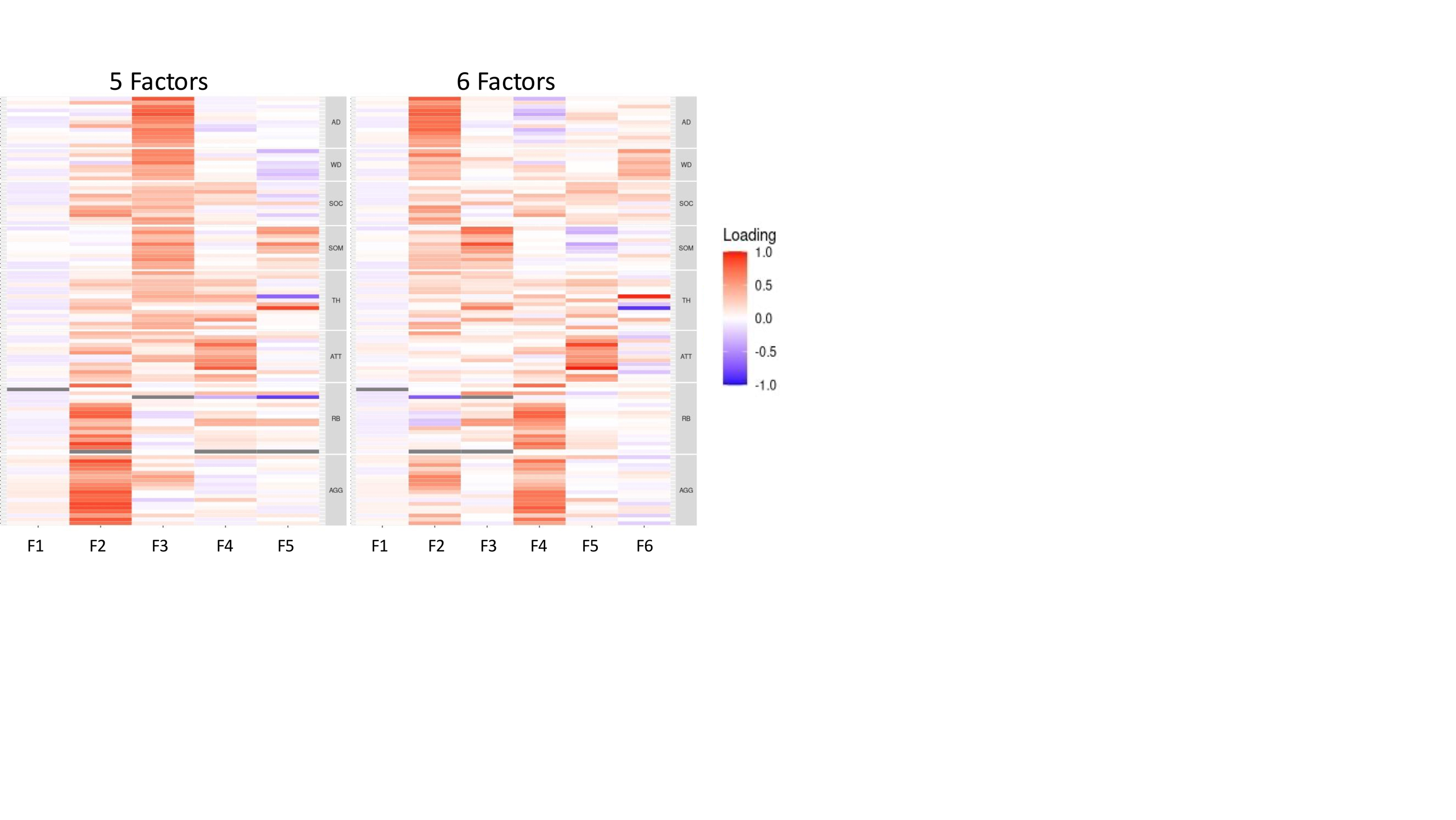


*Supplementary Figure 23.* Standardised loadings across all items in 5-8 ESEM factor solutions. Items are organised into their original subscales (AD = Anxious Depressed, WD = Withdrawn Depressed, SOC = Social Problems, SOM = Somatic Complaints, TH = Thought Problems, ATT = Attention Problems, RB = Rule Breaking, AGG = Aggressive Behaviours). Factors are plotted on the x-axis (F1 = Factor 1, F2 = Factor 2 etc.), items are plotted on the y-axis.
